# Operational definition of complementary, alternative, and integrative medicine derived from a systematic search

**DOI:** 10.1186/s12906-022-03556-7

**Published:** 2022-04-12

**Authors:** Jeremy Y. Ng, Tushar Dhawan, Ekaterina Dogadova, Zhala Taghi-Zada, Alexandra Vacca, L. Susan Wieland, David Moher

**Affiliations:** 1 Department of Health Research Methods, Evidence, and Impact, Faculty of Health Sciences, McMaster University, Hamilton, ON Canada; 2 Centre for Journalology, Clinical Epidemiology Program, Ottawa Hospital Research Institute, Ottawa, Canada; 3 Center for Integrative Medicine, University of Maryland School of Medicine, Baltimore, MD USA; 4 School of Epidemiology and Public Health, Faculty of Medicine, University of Ottawa, Ottawa, Canada

**Keywords:** Complementary and alternative medicine, Integrative medicine, Operational definition, Standard of classification

## Abstract

**Background:**

Identifying what therapies constitute complementary, alternative, and/or integrative medicine (CAIM) is complex for a multitude of reasons. An operational definition is dynamic, and changes based on both historical time period and geographical location whereby many jurisdictions may integrate or consider their traditional system(s) of medicine as conventional care. To date, only one operational definition of “complementary and alternative medicine” has been proposed, by Cochrane researchers in 2011. This definition is not only over a decade old but also did not use systematic methods to compile the therapies. Furthermore, it did not capture the concept “integrative medicine”, which is an increasingly popular aspect of the use of complementary therapies in practice. An updated operational definition reflective of CAIM is warranted given the rapidly increasing body of CAIM research literature published each year.

**Methods:**

Four peer-reviewed or otherwise quality-assessed information resource types were used to inform the development of the operational definition: peer-reviewed articles resulting from searches across seven academic databases (MEDLINE, EMBASE, AMED, PsycINFO, CINAHL, Scopus and Web of Science); the “aims and scope” webpages of peer-reviewed CAIM journals; CAIM entries found in online encyclopedias, and highly-ranked websites identified through searches of CAIM-related terms on HONcode. Screening of eligible resources, and data extraction of CAIM therapies across them, were each conducted independently and in duplicate. CAIM therapies across eligible sources were deduplicated.

**Results:**

A total of 101 eligible resources were identified: peer-reviewed articles (*n* = 19), journal “aims and scope” webpages (*n* = 22), encyclopedia entries (*n* = 11), and HONcode-searched websites (*n* = 49). Six hundred four unique CAIM terms were included in this operational definition.

**Conclusions:**

This updated operational definition is the first to be informed by systematic methods, and could support the harmonization of CAIM-related research through the provision of a standard of classification, as well as support improved collaboration between different research groups.

**Supplementary Information:**

The online version contains supplementary material available at 10.1186/s12906-022-03556-7.

## Background

Defining complementary, alternative and integrative medicine (CAIM) has been both complex and dynamic. The US National Center for Complementary and Integrative Health (NCCIH) defines “complementary” medicine as a non-mainstream practice that is used *together with* conventional medicine and “alternative” medicine as a non-mainstream practice used *in place of* it. They define “integrative health” as the bringing together of conventional and complementary approaches in a coordinated way [1]. In all three instances, these terms imply a relationship to conventional medicine, which may limit the categorization of therapies that have an undefined or non-existent relationship to conventional care [2]. Another challenge is that many of these terms are used interchangeably in the medical literature despite having marked differences in their meanings, and there is no universal consensus regarding which term ought to be used or is the most “correct”. Other, less frequently used terms to describe these therapies also include “unconventional”, “unorthodox”, and “non-mainstream”, which are all reflective of therapies that are not typically taught at and/or provided by Western health care systems. Historically, subsets of such therapies have also been referred to as “quackery” and “charlatanism”, among other pejorative terms that are generally not used to describe them in the medical literature today [2].

Despite the difficulties in defining CAIM, it is well-documented that therapies described as complementary, alternative, traditional, or integrative are used with a high prevalence across the world. Many patients use CAIM in combination with, and a minority in lieu of, conventional care. Eighty-eight percent of World Health Organization member states (170 countries) have acknowledged the use of CAIM, having formally developed policies, laws, regulations, programs and offices for CAIM, as examples [3, 4]. The prevalence of CAIM use among many Western countries is highly variable, though in some countries it can be high; for example, among Canadians it is approximately 80% [5]. Across European countries, CAIM use has been found to vary from 0.3% to 86% [6, 7]. The use of CAIM is also known to be highly prevalent among patients living with a wide range of diseases/conditions; in cancer patients, as many as 90% report using some form of CAIM [8–10]. While one reason for these large differences in the prevalence of CAIM use across different jurisdictions may indeed be attributed to cultural norms or true preferences for or against CAIM, another reason includes the fact that there is simply no consensus, and therefore standard, for what is or is not included in an operational definition of CAIM. Even national surveys, themselves, do not contain an identical list of therapies when compared across time within countries.

The popularity and acceptance of different CAIM therapies have also not remained equal, but instead have varied over time, culture, and geographical region. For example, in the 1900s, animal magnetism (also known as mesmerism) was a type of CAIM that had gained some popularity in Europe and the United States, though conventional medical practitioners at the time viewed it with skepticism [11, 12]. Today, however, it is largely unpracticed and could arguably be excluded from an operational definition of CAIM. Other CAIMs have gained increased popularity, as well as greater acceptance from conventional healthcare practitioners in some regions of the world, such as chiropractic [13], naturopathy [14, 15], acupuncture [16, 17], and traditional Chinese medicine [18, 19].

Regardless of how CAIM therapies gain popularity among patients, the reasons that motivate patients to use CAIM are well-studied and some of the most common ones include: symptom relief, improved quality of life, augmentation of conventional therapy, support of one’s philosophical orientations towards health, and achievement in control over one’s care [20–22]. Due to the popularity of these therapies in some populations [23] and even some significant results of efficacy [24], they could arguably be offered in conventional healthcare settings including family physician practices, hospitals and hospices. The very fact that patients actively choose to use CAIM therapies, with many lacking safety and efficacy profiles, justifies the conducting of research in this field. New knowledge gained in turn, can be incorporated into the medical curriculum, and can help inform shared decision-making between healthcare practitioners and patients. Unsurprisingly, it is also known that the quantity of CAIM research being conducted has increased greatly over the past few decades [2, 25].

A theoretical definition of CAIM, however, is arguably not enough to inform certain types of CAIM research. Aside from studies testing specific CAIM therapies, such as those conducted through randomized controlled trials, currently published systematic reviews and bibliometric analyses on CAIM in general address multiple CAIM therapies and lack complete search strategies [26]. This can be attributed to the fact that no standard list of CAIM therapies is agreed upon within the research community, largely due to the lack of an existing comprehensive operational definition. This, in turn, results in a great omission of potentially eligible studies across these research methods, which yield biased or incomplete results. This justifies the development of an operational definition of CAIM, which if comprehensive, can serve as a solution. An operational definition serves a different purpose than a theoretical definition, as it identifies all (if not, as many as possible) therapies that can be categorized as CAIM, yet it is also a far more challenging definition to create. Like CAIM itself, an operational definition of it is dynamic, changing based on both historical time period in light of new evidence generated from medical research, and geographical location whereby many jurisdictions may integrate or consider their traditional system(s) of medicine as conventional care.

### Developing an operational definition of CAIM

To date, only one operational definition of complementary and alternative medicine (CAM, not CAIM) has been published by Wieland et al. in 2011. They began by considering ways in which the 2005 Institute of Medicine theoretical definition of CAM, as therapies “other than those intrinsic to the politically dominant health system of a particular society or culture in a given historical period”, was relevant to the landscape of the early twenty-first century. To obtain a list of specific therapies, they examined sources in the US National Library of Medicine (NLM) PubMed database including the Medical Subject Heading (MeSH) definition of “Complementary Therapies” and the CAM on PubMed subset search strategy. They created an initial list of 70 alphabetical CAM terms or combinations of terms under subtopics according to the 5 categories of CAM therapies set by the National Center for Complementary and Alternative Medicine (NCCAM, the former name of the NCCIH at the time), and then subjected these terms to further qualifications and refinements based on setting, route of administration, and therapy/indication pairings [27]. Although the term “integrative” medicine had been in use as early as 1995 [28, 29], it remained a relatively infrequently used term at the time that Wieland et al.’s study was published [2]; thus, it is understandable why their definition did not include this term. Over the last decade, however, the use of the term “integrative” to refer to such therapies has become increasingly popular by both healthcare practitioners and researchers alike [2, 30, 31]. Considering that the NCCAM was renamed the National Centre for Complementary and Integrative Health (NCCIH) in December 2014, the update to their name is significant and indicates the emergence of patient, clinician, researcher and policy maker interest in an integrative approach in medical treatment plans [32]. It thus follows that a new operational definition that includes “integrative” is created.

Despite the omission of the term “integrative”, Wieland et al.’s study is a valuable starting point for the development of the present study’s operational definition, as they detailed how they constructed their definition, such as considering the historical context of a therapy, whether it is a standard treatment within the dominant medical system, whether it is a standard treatment for a given condition, and the setting in which the therapy is provided [27]. A number of sentiments shared by Wieland et al., with respect to the value of an operational definition, therefore, equally apply to the present study. An operational definition of CAIM would support the harmonization of research, as CAIM-specific research databases can be developed in a more standardized fashion with respect to classifying what constitutes included therapies. This may also allow for more effective collaboration among research groups, as a general consensus can be reached rapidly [27]. Operationalization also enables the precise comparison of different CAIM areas over time and across investigators [27]. Undoubtedly, value has emerged from Wieland et al.’s (2011) work, as a variety of studies have utilized their operational definition to inform their research. Some examples include a literature review of traditional and complementary medicine in the context of mental health services in low- or middle-income countries [33] and a systematic review of the cost-effectiveness of common complementary and integrative therapies [34]. Studies have also used Wieland et al.’s operational definition with some modification [6], or in combination with other approaches to propose an operational definition for clinical pathways [35].

The methods used by Wieland et al. (2011) to construct their operational definition, was not without its weaknesses, however, and the present study aims to update and build on their work in a few ways. Wieland et al. (2011) only reviewed two sources within the US National Library of Medicine’s PubMed database, the MeSH definition of "Complementary Therapies" and the Complementary Medicine subset search strategy, to generate a listing of specific therapies [27]. In light of these shortcomings, the objective of the present study was to create an operational definition of CAIM derived from a systematic search of four peer-reviewed or otherwise quality-assessed information resource types.

## Methods

### Approach

Preliminary searches were conducted to identify studies that have provided an operational definition of CAIM (and CAIM-related terms), of which only Wieland et al.’s (2011) study was found. Following this, searches were conducted during the weeks of August 24 and 31, 2020 across four different types of quality-assessed media, including 1) peer-reviewed articles from seven major databases (MEDLINE, EMBASE, AMED, PsycINFO, CINAHL, Scopus and Web of Science, searched systematically), 2) “Aims and Scope” webpages of peer-reviewed CAIM journals, 3) entries containing CAIM therapies in highly-accessed online encyclopaedias, and 4) highly ranked websites resulting from Health On the Net Code of Conduct (HONcode) searches. These four types of media were specifically selected, as they have been deemed to contain quality-assessed information. Peer-reviewed articles and peer-reviewed CAIM journals are both academic authorities on CAIM research. Highly accessed encyclopedias contain entries written by experts in their corresponding fields; they are fact-checked and frequently are also peer-reviewed. Websites present on HONcode are assessed for reliability and credibility of information, and must meet specific criteria in order to be certifiable and appear in HONcode searches (unlike Google or Yahoo!, for example). Eligible items identified across all four resource types were reviewed for therapies relating to CAIM (and CAIM-related terms, such as “complementary”, “alternative” or “integrative” medicine), which were then data extracted for inclusion in the operational definition of CAIM. A protocol was registered with the (Prospective Register of Systematic Reviews) PROSPERO, registration number CRD42020206301. It should be noted that in our PROSPERO registration, we also describe the development of academic database search strategies informed by this operational definition, which we plan to publish separately. At present, a preprint of this second manuscript is available here: 10.21203/rs.3.rs-1390385/v1.

### Eligibility criteria

Based on the differing nature of these four media sources, we applied specific inclusion criteria to each in order to inform the development of an operational definition of CAIM. Peer-reviewed articles were deemed eligible if they provided a list or group of CAIMs, or CAIM-related terms (e.g. a list of alternative medicines, etc.). We specifically excluded articles that did not provide a list or group of CAIM therapies, as this would have been extremely time-consuming with little return in benefit, as the vast majority of these articles pertained to the study of a single CAIM. Peer-reviewed journals’ “Aims and Scope” webpages were deemed eligible if the title of the journal contained the words “complementary”, “alternative”, and/or “integrative”. Highly-accessed online encyclopaedias were eligible if they were not publicly editable (e.g. Wikipedia entries were excluded) and contained entries relating to complementary, alternative and/or integrative/integrated medicine. As we sought to develop an operational definition, we did not include encyclopaedia entries about specific CAIMs (e.g. chiropractic, acupuncture, etc.). Highly ranking websites which appeared within the first 20 results (or first two search results pages) of each HONcode search were reviewed; websites were deemed eligible if they contained any CAIM therapies (i.e. therapies explicitly described/listed as a CAIM [or synonym thereof, e.g. complementary medicine, alternative medicine, integrative medicine] on the website itself). HONcode search results also yielded peer-reviewed articles, which were treated based on the eligibility criteria of peer-reviewed articles. Any items not published in the English language were excluded across all four media types.

### Searching and screening

MEDLINE, EMBASE, AMED, PsycINFO, CINAHL, Scopus and Web of Science (WoS) were searched from inception of each respective database on August 25th, 2020. The search strategies were developed by JYN and included terms that commonly refer to CAIM [1]. Eligible full-text articles were identified by screening abstracts and titles independently and in duplicate. Citations of these articles were also checked to retrieve new literature to contribute to the operational definition. Eligible “Aims and Scope” webpages were identified using Scimago (https://www.scimagojr.com/ [“Complementary and Alternative Medicine” Category]) and Journal Citation Reports (https://jcr.clarivate.com/ [“Integrative and Complementary Medicine” Category]). Highly-accessed online encyclopaedias were identified via Alexa ranking [36]. The search strategies run on the seven databases can be found in Table 1.

**Table 1:** Academic Database Search Strategies Informing the Development of an Operational Definition of Complementary, Alternative, and Integrative Medicine, Executed August 25, 2020

**Ovid Database (MEDLINE, EMBASE, PsycINFO, and AMED)**
Database: AMED (Allied and Complementary Medicine) < 1985 to August 2020 > , Embase < 1974 to 2020 August 24 > , APA PsycInfo < 1806 to August Week 3 2020 > , Ovid MEDLINE(R) and Epub Ahead of Print, In-Process & Other Non-Indexed Citations, Daily and Versions(R) < 1946 to August 24, 2020 > Search Strategy: –––––––––––––––––––––––––––––––––––––––– 1 ((Alternative or Complementary or Integrat*) adj5 (Therap* or Medicine*)).ti. (39,659) 2 (Character* or Definition* or Delineat* or Expla* or Interpret* or Meaning* or Term*).ti. (2,380,379) 3 1 and 2 (610) 4 limit 3 to english language (527) ***************************
**Scopus Database**
TITLE(((Alternative or Complementary or Integrat*) W/5 (Therap* or Medicine*)) AND (Character* or Definition* or Delineat* or Expla* or Interpret* or Meaning* or Term*)) AND ( LIMIT-TO ( LANGUAGE,"English"))
**Web of Science Database**
TITLE: (((Alternative or Complementary or Integrat*) NEAR/5 (Therap* or Medicine*))) AND TITLE: ((Character* or Definition* or Delineat* or Expla* or Interpret* or Meaning* or Term*)) AND LANGUAGE: (English) Indexes = SCI-EXPANDED, SSCI, A&HCI, CPCI-S, CPCI-SSH, BKCI-S, BKCI-SSH, ESCI, CCR-EXPANDED, IC Timespan = All years

Highly-ranking websites were identified by means of search strategies developed by JYN, relating to CAIM definitions via HONcode searches [37]. Twenty terms were searched on https://www.hon.ch/en/search.html and sorted by “Relevance” and “All” including: “alternative medicine search strategy”; “complementary medicine search strategy”; “integrated medicine search strategy”; “integrative medicine search strategy”; “list of alternative medicine”; “list of complementary medicine”; “list of integrated medicine”; “list of integrative medicine”; “operational definition of alternative medicine”; “operational definition of complementary medicine”; “operational definition of integrated medicine”; “operational definition of integrative medicine”; “types of alternative medicine”; “types of complementary medicine”; “types of integrated medicine”; and “types of integrative medicine”. The first two pages (first 20 results) were reviewed for each search, totalling 320 webpages (16 search terms).

TD and ED screened the titles and abstracts of peer-reviewed articles recovered from MEDLINE, EMBASE, AMED, PsycINFO, CINAHL, Scopus, Web of Science and HONcode independently and in duplicate. TD and ED screened a subset of the CAIM journal “Aims and Scope” webpages independently and in duplicate. ZT and AV screened CAIM entries in highly-accessed online encyclopaedias, HONcode searches, and a subset of the journal “Aims and Scope” webpages, both independently and in duplicate. Duplicates resulting from searches between and across all four media sources were removed. After all the items were screened, TD, ED, ZT, and AV met with JYN to discuss and resolve discrepancies. These results were checked over an additional time by LSW and DM.

### Data extraction and analysis

The full-texts of each eligible item was saved offline, and these versions were used to conduct our data extraction and analysis, as opposed to revisiting the online sources, to ensure that any content did not change over time. ZT and AV data extracted the names of CAIM therapies from eligible HONcode searches, CAIM-related entries in the most-visited online encyclopaedias, and journals’ “Aims and Scopes” webpages, in addition to those from the the Cochrane operational definition of complementary medicine, independently and in duplicate. TD and ED extracted the names of CAIM therapies from eligible peer-reviewed articles, including those resulting from HONCode searches, independently and in duplicate. After all the names of CAIM therapies were data extracted, TD, ED, ZT, and AV met with JYN to discuss and resolve discrepancies. These results were checked over an additional time by LSW and DM.

### Creation of a CAIM operational definition

A list of all names of CAIM therapies yielded from all data extractions across were compiled into a Microsoft Excel spreadsheet, and duplicates were removed. Given the large number of CAIM therapies identified, TD, ED, ZT, and AV reviewed each item an additional time for accuracy. Upon finalizing the list of CAIM therapies included in the operational definition, we grouped similar/identical CAIM therapies (e.g. “St. John’s wort” and “Hypericum perforatum”) together to appear on a single line, then alphabetized all lines. A certain degree of evaluator judgement was exercised in determining 1) whether a therapy was considered CAIM and 2) whether one or more CAIMs were similar/identical to one another. To guide our decisions, we also consulted monographs published by the Natural Medicines Research Collaboration [38] to identify all common and scientific names pertaining to each CAIM therapy; in cases where no professional monograph was available, we consulted the peer-reviewed literature. We note that we did not discriminate between very broad (i.e. “complementary and alternative medicine”) and highly specific (i.e. “St. John’s wort”) CAIMs; therefore, we included any term that we identified that referred to a single or a group of CAIM(s) or this category of therapies altogether. We also note that we included therapies that may serve as CAIM in one context and conventional medicine in another; these were included based on the fact that they were denoted as CAIM in the original source found through our systematic search. Additionally, JYN and LSW have considerable experience studying CAIM, and DM is a highly experienced research methodologist; both assisted in guiding the other authors in making a finalized judgement resulting in the finalized operational definition of CAIM.

## Results

**Fig. 1:**
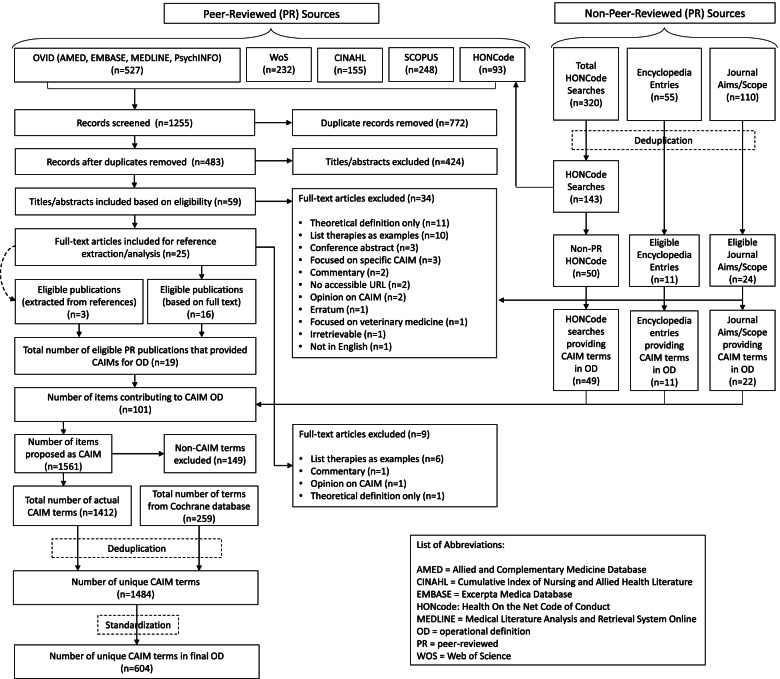
PRISMA Diagram

The search conducted on OVID databases (AMED, EMBASE, MEDLINE, PsycINFO), Web of Science, CINAHL, Scopus and HONCode peer-reviewed sources retrieved a total of 1255 results, of which a total of 483 were unique. From the 483 results, 424 titles/abstracts were excluded, leaving 59 results that were deemed eligible based on their title/abstracts. This was followed by 34 further exclusions, leaving 25 results deemed eligible based on their full text. Of the remaining 25 full-text articles, 9 were excluded, resulting in 16 full-text publications to remain. Three additional publications were retrieved from the citations of the 16 full-texts, totaling 19 full-text publications. Thus, 19 journal articles contributed to providing CAIM terms for the operational definition. In terms of non peer-reviewed article sources, 22 journals from SCIMAGO and JCR, 11 encyclopedias from Alexa rankings, and 49 eligible non-peer reviewed article HONCode search entries were also reviewed for contribution to the CAIM terms for the operational definition. Overall, a total of 101 items were obtained from both peer-reviewed and non-peer reviewed sources; a complete bibliography of these items are provided in Supplementary File 1. Our final operational definition in this study includes a total of 604 unique CAIM terms, based on the 1561 terms CAIM terms extracted from the final 101 items in addition to the 259 terms retrieved from the Cochrane operational definition of complementary medicine. A modified Preferred Reporting Items for Systematic Reviews and Meta-Analyses (PRISMA) diagram depicting all searched, screened, and included items is shown in Fig. 1.

### Operational definition of CAIM

Of the 1561 final terms that underwent further analysis, 149 terms were removed as they were not considered CAIM, resulting in 1412 terms. The 259 terms retrieved from the Cochrane Complementary Medicine operational definition of complementary medicine were screened for CAIM terms that were not already included in the list of final terms, after which 82 were deemed to be unique, and subsequently added to the operational definition on March 18, 2021. After these changes, a total of 1494 terms were standardized as informed by the process of proximity searching; terms were standardized under keywords or phrases that typically appear in approximate sets of words in literature. After standardization, the final list of CAIM therapies included in our study was 604 as they described the fundamental nature of CAIM rather than actual CAIM therapies. The complete operational definition of CAIM is provided in Table 2.

**Table 2:** Operational Definition of Complementary, Alternative and Integrative Medicine

1. 5-Hydroxytryptophan (Scientific name: 2-Amino-3-(5-Hydroxy-1H-Indol-3-yl)-Propanoic Acid, 5-Hydroxytryptophan, L-5 Hydroxytryptophan, 5-hydroxytryptophan, L-5 hydroxytryptophan. Also known as: 5 Hydroxy-Tryptophan, 5 Hydroxy-Tryptophane, 5-Hydroxytryptophan, 5-Hydroxytryptophane, 5-Hydroxy L-Tryptophan, 5-Hydroxy L-Tryptophane, 5-Hydroxy Tryptophan, 5-L-Hydroxytryptophan, L-5 HTP, L-5-Hydroxytryptophan, L-5-Hydroxytryptophane, Oxitriptan.)
2. 714-X
3. Aboriginal Medicine
4. Abscisic Acid
5. Acai (Scientific name: Euterpe oleracea, synonym Euterpe badiocarpa. Also known as: Açaï, Acai Berry, Açaï d'Amazonie, Acai Extract, Acai Fruit, Acai Palm, Amazon Acai, Amazon Acai Berry, Assai, Assai Palm, Baie d'Açaï, Baie de Palmier Pinot, Cabbage Palm, Chou Palmiste, Extrait d'Açaï, Fruit d'Açaï, Palmier d'Açaï.)
6. Active Therapy
7. Acumoxa
8. Acupressure (Also known as: Active Acupressure, Acupresión, Acupression, Acupression Active, Acupression Auriculaire, Acupression Chinoise, Acupression Occidentale,Acupression Passive, Acupression du Pied, Aroma Acupressure, Aromatherapy Acupressure, Auricular Acupressure, Chinese Acupressure, Ear Acupressure, Foot Acupressure, Nei Guan, Neiguan Point Acupressure, P6 Acupressure, Passive Acupressure, San Yin Jiao Acupressure, Self-Acupressure, SP6 Acupressure, Tapas Acupressure Technique, Traditional Chinese Acupressure, Western Acupressure.)
9. Acupuncture (Also known as: Acuponcture, Acuponcture Auriculaire, Acuponcture Chinoise, Acuponcture Coréenne, Acuponcture Japonaise, Acuponcture de la Main, Acuponcture Occidentale, Acuponcture de l'Oreille, Acuponcture du Pied, Acupuntura, Auricular Acupuncture, Chinese Acupuncture, Ear Acupuncture, Electroacupuncture, Foot Acupuncture, Hand Acupuncture, Japanese Acupuncture, Korean Acupuncture, Laser Acupuncture, Needle Moxibustion, Single Point Acupuncture, Trigger Point Acupuncture, Western Acupuncture.)
10. Acustimulation (Also known as: Acupoint Stimulation, Acupuncture Point Stimulation, Acustimulation Wristbands, EA, Electroacupuncture, TAES, TEAS, Transcutaneous Acupoint Electrical Stimulation, Transcutaneous Electrical Acupoint Stimulation, Transcutaneous Electrical Acustimulation.)
11. Acutonics
12. Aerobics (Also known as: Aerobic Exercise.)
13. Agrimony (Scientific name: Agrimonia eupatoria. Also known as: Agrimone, Agrimonia, Aigremoine, Aigremoine Eupatoire, Church Steeples, Churchsteeples, Cockeburr, Cocklebur, Common Agrimony, Da Hua Long Ya Cao, Eupatoire-des-Anciens, Fragrant Agrimony, Francormier, Herba Agrimoniae, Herbe-de-Saint-Guillaume, Herbe de Sainte Madeleine, Philanthropos, Soubeirette, Sticklewort, Thé des Bois, Thé du Nord, Toute-Bonne.)
14. Aikido (Also known as: Gendai Budo, Kata.)
15. Aiyishu
16. Alcoholics Anonymous
17. Alexander Technique (Also known as: Alexander Proprioception, AT, F.M. Alexander Technique, Technique Alexander, Técnica Alexander.)
18. Allicin
19. Aloe (Scientific name: Aloe vera, synonyms Aloe barbadensis, Aloe indica, Aloe africana, Aloe arborescens, synonyms Aloe natalenis, Aloe frutescens, Aloe ucriae, Aloe ferox, synonym Aloe supralaevis, Aloe perryi, Aloe spicata. Also known as: Aloe Capensis, Aloe Gel, Aloe Latex, Aloe Leaf Gel, Aloe Perfoliata, Aloe Vera Barbenoids, Aloe Vera Gel, Aloes, Aloès, Aloès de Curaçao, Aloès des Barbades, Aloès du Cap, Aloès Vrai, Aloès Vulgaire, Barbados Aloe, Burn Plant, Cape Aloe, Chritkumari, Curacao Aloe, Elephant's Gall, Gel de la Feuille d'Aloès, Ghee-Kunwar, Ghi-Kuvar, Ghrita-Kumari, Gvar Patha, Hsiang-Dan, Indian Aloe, Jafarabad Aloe, Kanya, Kidachi Aloe, Kumari, Latex d'Aloès, Lily of the Desert, Lu-Hui, Miracle Plant, Plant of Immortality, Plante de l'Immortalité, Plante de la Peau, Plante de Premiers Secours, Plante Miracle, Plantes des Brûlures, Sábila.)
20. Alpha-Linolenic Acid
a. Alpha-Linolenic Acid (ALA) (Scientific name: Alpha-Linolenic Acid. Also known as: Acide Alpha-Linolénique, Ácido Alfa Linolénico, Acide Gras Essentiel, ALA, Acide Linolénique, Acide Gras N3, Acide Gras Oméga 3, Acide Gras Polyinsaturé Oméga 3, Acide Gras Polyinsaturé N3, Essential Fatty Acid, Linolenic Acid, LNA, N-3 Fatty Acid, N-3 Polyunsaturated Fatty Acid, Omega 3, Omega 3 Fatty Acids, Omega-3, Omega-3 Fatty Acids, Omega-3 Polyunsaturated Fatty Acid.)
b. Flaxseed Oil (Scientific name: Linum usitatissimum, synonyms Linum crepitans, Linum humile. Also known as: Aceite de Linaza, Acide Alpha-Linolénique, Acide Gras N-3, Acide Gras Oméga 3, ALA, Aliviraaii, Alpha-Linolenic Acid, Alasi, Brown Flaxseed Oil, Brown-Seeded Flax Oil, Common Flax Oil, Echter Lein, Flachs, Flachssamen, Flax Oil, Flax Seed Oil, Golden Flax Oil, Graine De Lin, Huile de Lin, Kattan, Keten, Lin, Lin Commun, Lin Oléagineux, Linho, Lino, Lino Comune, Lino Mazzese, Lino Usuale, Linseed Flax Oil, Linseed Oil, Malsag, N-3 Fatty Acid, Oil of Flaxseed, Omega-3 Fatty Acid, Saatlein, Ta Ma, Tisii.)
21. Alpha-Lipoic Acid (Scientific name: 1,2-dithiolane-3-pentanoic acid; 1,2-dithiolane-3-valeric acid; 6,8-thioctic acid; 5-(1,2-dithiolan-3-yl) valeric acid; 6,8-dithiooctanoic acid. Also known as: A-Lipoic Acid, Acetate Replacing Factor, Acide Alpha-Lipoïque, Acide Alpha-Lipoïque R, Acide DL-Alpha- Lipoïque, Acide Lipoïque, Acide Thioctique, Acide 1,2-dithiolane-3-pentanoïque, Acide 1,2-dithiolane-3-valérique, Acide 5 Valérique (1,2-dithiolan-3-yl), Acide 6,8-dithiooctanoïque, Acide 6,8-Thioctique, Acido Alfa Lipoico, ALA, Biletan, DHLA, Dihydrolipoic acid, Extrait d'acide Alpha-Lipoïque, Lipoic Acid, Lipoicin, R-ALA, R-Alpha-Lipoic Acid, (+ -)-1,2-Dithiolane-3-Pentanoic Acid, (R)-Dithiolane-3-Pentanoic Acid, R, S-Alpha Lipoic Acid, (R)-Lipoic Acid, R-Lipoic Acid, RS-Alpha-Lipoic Acid, S- Alpha-Lipoic Acid, S-Lipoic Acid, Sodium-R-Lipoate, Thioctacid, Thioctan, Thioctic Acid.)
22. Alternative Medicine (Also known as: Alternative Therapy, Alternative Approach, Alternative Health.)
23. Amino Acid Therapy (Also known as: CAAT, Controlled Amino Acid Therapy.)
24. Amma Therapy
25. Ammotherapy
26. Ancient Medicine
27. Androstenedione (Scientific name: 4-androstene-3,17-dione, Androst-4-ene-3,17-dione. Also known as: 4-Androstene 3, 17-dione, Andro, Androstene, Androstenediona, Androsténédione.)
28. Animal Assisted Therapy (Also known as: Animal-Assisted Activities (AAA), Animal-Assisted Education, Animal-Assisted Intervention (AAI), Animal-Facilitated Therapy, Animal Companionship, Animals In Human Therapy, Animal Visitation, Canine-Assisted Ambulation, Canine-Assisted Therapy, Canine Therapy, Canine Visitation Therapy (CVT), Companion Animal Therapy, Dog-Assisted Therapy, Dolphin-Assisted Therapy, Equine-Assisted Activity, Equine-Assisted Activities and Therapies (EAAT), Equine-Assisted Therapy, Equine Therapy, Hippotherapy, Horse-Riding Therapy, Pet-Assisted Therapy, Pet-Facilitated Therapy, Pet Therapy, Psychoeducational Horseback Riding, Therapeutic Horseback Riding.)
29. Animal Extract
30. Aniministic Practice
31. Anma
32. Anthocyanin
33. Anthrophysical Medicine
34. Anthroposophic Medicine
35. Antineoplastons (Scientific name: 3-Phenylacetylamino-2,6-piperidinedione, Phenylacetate, Phenylacetylglutamine, Phenylacetylisoglutamine. Also known as: Antineoplaston A, Antineoplaston A1, Antineoplaston A10, Antineoplaston A10-1, Antineoplaston A2, Antineoplaston A3, Antineoplaston A4, Antineoplaston A5, Antineoplaston AS2-1, Antineoplaston AS2-5, Antineoplaston AS5, Antineoplaston Ch, Antineoplaston F, Antineoplaston H, Antineoplaston K, Antineoplaston L, Antineoplaston O.)
36. Antioxidant
37. Apitherapy (Also known as: Apipuncture, Apis Mellifera Venom, Apis Venenum Purum, Apiterapia, Api-Therapy, Api-Treatment, Apithérapie, Api-Venin-Thérapie, Bee Sting Therapy, Bee Therapy, Bee Treatment, Bee Venom Therapy, BVT, Honey Bee Venom Therapy, Thérapie des Abeilles, Thérapie par Venin d'Abeille, Traitement par Piqûres d'Abeilles.)
38. Applied Biomechanics
39. Applied Kinesiology (Also known as: AK, Health Kinesiology, Kinésiologie Appliquée, Kinésiologie Éducative, Kinesiology Muscle Test, Quinesiología, Test Musculaire de la Kinésiologie.)
40. Arachidonic Acid
41. Aristolochia (Scientific name: Aristolochia auricularia; Aristolochia clematitis; Aristolochia fangchi; Aristolochia heterophylla; Aristolochia kwangsiensis; Aristolochia manshuriensis; Aristolochia moupinensis; Aristolochia reticulata; Aristolochia serpentaria; other Aristolochia species. Also known as: Aristoloche, Aristoloche Clématite, Aristoloche de Texas, Aristoloche des Vignes, Aristoloche de Virginie, Aristoloche Vulgaire, Aristoloquia, Birthwort, Guan Mu Tong, Guang Fang Ji, Long Birthwort, Pelican Flower, Poison de Terre, Pomerasse, Ratelaine, Rateline, Red River Snakeroot, Sangree Root, Sangrel, Sarrasine, Serpentaire, Serpentaire de la Rivière Rouge, Serpentaria, Snakeroot, Snakeweed, Texas Snakeroot, Virginia Serpentary, Virginia Snakeroot.)
42. Arnica (Scientific name: Arnica montana, Arnica fulgens, Arnica sororia, Arnica latifolia, Arnica cordifolia, Arnica angustifolia, Arnica chamissonis. Also known as: American Arnica, Arctic Arnica, Arnica des Montagnes, Arnica Flos, Arnica Flower, Arnikablüten, Bergwohlverleih, Doronic d'Allemagne, European Arnica, Fleurs d'Arnica, Foothill Arnica, Heart-Leaf Arnica, Herbe aux Chutes, Herbe aux Prêcheurs, Hillside Arnica, Kraftwurz, Leopard's Bane, Mountain Arnica, Mountain Snuff, Mountain Tobacco, North American Meadow Arnica, Plantin des Alpes, Quinquina des Pauvres, Souci des Alpes, Tabac des Savoyards, Tabac des Vosges, Twin Arnica, Wolf's Bane, Wolfsbane, Wundkraut.)
43. Aromatherapy (Also known as: Aroma, Aroma Therapy, Aroma Treatment, Aromaterapia, Aromathérapie, Aromatic Oils, Aromatic Therapy, Essential Oils, Huiles Aromatiques, Huiles Essentielles, Scent Therapy, Traitement par les Essences de Plantes.)
44. Art Therapy (Also known as: Anthroposophic Art Therapy, Art Stimulation, Chinese Calligraphy Therapy, Clay Art Therapy, Creative Arts Intervention, Creative Arts Therapy, Creative Expression, Mindfulness Based Art Therapy, Phenomenological Art Therapy, Psychodynamic Group Art Therapy, Visual Art Therapy.)
45. Artemisia Herba-Alba (Scientific name: Artemisia herba-alba. Also known as: Absinthe du Désert, AHAE, AHE, Armoise Herbe Blanche, Artemisia, Chih, Common Wormwood, Common Worm Wood, Desert Wormwood, Herba Alba, Shih, Thym des Steppes.)
46. Artichoke (Scientific name: Cynara cardunculus, synonym Cynara scolymus. Also known as: Alcachofa, Alcaucil, ALE, Artichaut, Artichaut Commun, Artichaut Sauvage, Artichoke Extract, Artichoke Fruit, Artichoke Leaf, Artichoke Leaf Extract, Artischocke, Cardo, Cardo de Comer, Cardon d'Espagne, Cardoon, Cynara, Extrait d'Artichaut, Feuille d'Artichaut, Garden Artichoke, Gemuseartischocke, Globe Artichoke, Kardone, Tyosen-Azami, Wild Egyptian Artichoke.)
47. Asian Diet
48. Astragaloside
49. Astragalus (Scientific name: Astragalus membranaceus, synonym Phaca membranacea, Astragalus mongholicus. Also known as: Astragale, Astragale à Feuilles de Réglisse, Astragale Queue-de-Renard, Astragale Réglissier, Astragali, Astragali Membranaceus, Astragalo, Astragli Membranceus, Beg Kei, Bei Qi, Buck Qi, Chinese Astragalus, Huang Qi, Huang Se, Huanggi, Hwanggi, Membranous Milk Vetch, Membranous Milkvetch, Milk Vetch, Mongolian Milk, Mongolian Milkvetch, Ogi, Radix Astragali, Radix Astragalus, Réglisse Bâtarde, Réglisse Sauvage.)
50. Atkins Diet (Also known as: Atkins, Dieta de Atkins, Dr. Robert Atkins' Diet, High Protein Diet, LCD, LCHPD, Low-Carb Diet, Low- Carbohydrate Diet, Low-Carbohydrate High-Protein Diet, Low Carb Diet, Low Carbohydrate Diet, Régime Atkins, Régime d'Atkins, Régime du Dr Robert Atkins, Régime Hyperprotéiné, Régime Hyperprotidique, Régime Hypoglucidique.)
51. Atractylis gummifera
52. Auriculotherapy/Auricular Therapy
53. Autogenic Training (Also known as: AT, Autogenic, Autogenic Relaxation, Autogenic Relaxation Training, Autogenic Relaxation Technique, Autorelaxation Concentrative, Autosuggestion, Entraînement Autogène, Entrenamiento Autógeno, Méthode de Schultz, Relaxation Therapy, Standard Autogenic Training, Training Autogène, Training Autogène de Schultz.)
54. Aversion Therapy
55. Ayurveda (Also known as: Ayurvéda, Ayurvedic Medicine, Médecine Ayurvédique, Médecine Traditionnelle Asiatique, Médecine Traditionnelle Indienne, TAM, TIM, Traditional Asian Medicine, Traditional Indian Medicine.)
56. Ba Wei Di Huang Wan (Also known as: Shen Qi Wan, Jin Gui Shen Qi Wan.)
57. Bach Flower Remedies (Also known as: Bach, Bach Flower, Bach Flower Essence, Bach Flower Remedies, Bach Flower Remedy, Bach Remedies, Batch Flower Remedies, BFR, BFRs, Edward Bach Remedies, Élixirs Floraux du Docteur Bach, Essences Florales de Bach, Fleurs de Bach, Florathérapie, Flower Dilutions, Flower Essence, Flower Essence Dilution, Flower Remedies, Flower Remedies, Remèdes Floraux, Remèdes Floraux de Bach, Remèdes Floraux du Dr Bach, Remedios Florales de Bach.)
58. Balance Technique
59. Balneotherapy (Also known as: Bain de la Mer Morte, Bain Minéral, Bain de Soufre, Bain Thermal, Balneological Treatment, Balneoterapia, Balneotherapeutics, Balnéothérapie, Balneum, Bath Therapy, Bath Treatment, Crenobalneotherapy, Dead Sea Baths, Hydrotherapy, Low-Dose Radon Hyperthermia Balneo Treatment, Mineral Bath, Radon Balneotherapy, Spa Therapy, Sulfur Baths, Therapeutic Bathing, Thérapie Thermale, Thermal Baths, Thermal Mineral Baths, Thermal Therapy, Traitement par le Bain, Water Therapy.)
60. Bay Leaf (Scientific name: Laurus nobilis. Also known as: Bay, Bay Laurel, Bay Leaf, Bay Tree, Daphne, Grecian Laurel, Laurel, Laurel Común, Laurier d'Apollon, Laurier Noble, Laurier-Sauce, Laurier Vrai, Mediterranean Bay, Noble Laurel, Roman Laurel, Sweet Bay, Sweet Laurel, True Bay, True Laurel.)
61. Bear Bile
62. Behaviour Medicine
63. Berberine (Scientific name: Berberine. Also known as: Alcaloïde de Berbérine, Berberina, Berbérine, Berberine Alkaloid, Berberine Complex, Berberine Sulfate, Sulfate de Berbérine, Umbellatine.)
64. Beta-Carotene (Scientific name: Beta-Carotene. Also known as: A-Beta-Carotene, A-Bêta-Carotène, Bêta-Carotène, Bêta-Carotène Tout Trans, Beta-Caroteno, Carotenes, Carotènes, Carotenoids, Caroténoïdes, Caroténoïdes Mélangés, Mixed Carotenoids, Provitamin A, Provitamine A.)
65. Bibliotherapy
66. Bilberry (Scientific name: Vaccinium myrtillus. Also known as: Airelle, Arándano, Bilberry Fruit, Bilberry Leaf, Black Whortles, Bleaberry, Brimbelle, Burren Myrtle, Dwarf Bilberry, Dyeberry, European Bilberry, Feuille de Myrtille, Fruit de Myrtille, Gueule Noire, Huckleberry, Hurtleberry, Mauret, Myrtille, Myrtille Européenne, Myrtilli Fructus, Raisin des Bois, Swedish Bilberry, Trackleberry, Whortleberry, Wineberry.)
67. Biochemical Therapy
68. Biodynamic Therapy
69. Biofeedback (Also known as: Assisted Biofeedback, Biofeedback Therapy, Biofeedback Training, Biofeedback de Variabilité Cardiaque, Bio-rétroaction, Biorretroalimentación, EEG Biofeedback, Electroencephalogram Biofeedback, Electromyography Biofeedback, EMG Biofeedback, Heart Rate Variability Biofeedback, HRV Biofeedback, Neurofeedback, Neuro-Rétroaction, Rétroaction Biologique, Rétroaction Biologique par EEG, Rétroaction Biologique par Électro-encéphalogramme, Rétroaction Biologique par EMG, Rétroaction Biologique par Électromyographie, Rétroaction Biologique de la Variabilité de la Fréquence Cardiaque, Rétroaction Biologique de la Variabilité du Rythme Cardiaque, Rétrocontrôle Biologique.)
70. Biofunctional Diagnostic Testing
71. Biologic Treatment
72. Bioresonance (Also known as: Appareil Mora, Biocom, Biocommunication, Biophysical Information, Biorésonance, Bioresonance Therapy, Biorresonancia, Information Biophysique, Mora Device, Mora Therapy, Morathérapie, Multicom, Multiresonance, Multirésonance, Thérapie Mora.)
73. Bitter Orange (Scientific name: Citrus aurantium, synonyms Citrus amara, Citrus bigarradia, Citrus vulgaris. Also known as: Aurantii Fructus, Aurantii Fructus Immaturus, Aurantii Pericarpium, Aurantium, Bigarade, Bigarade Orange, Bitter Orange Flower, Bitter Orange Peel, Chao Zhi Ke, Chisil, Citrus Aurantium Fruit, Extrait de Zeste d'Orange, Fleur d'Orange Amère, Flos Citri Auranti, Fructus Aurantii, Fructus Aurantii Immaturus, Green Orange, Kijitsu, Marmalade Orange, Meta-Synephrine, N-Methyltyramine, Naranja Amarga, Neroli Oil, Norsynephrine, Octopamine, Octopamine HCl, Orange Amère, Orange de Séville, Orange Peel Extract, Orange Verte, Seville Orange, Shangzhou Zhiqiao, Sour Orange, Synephrine, Synéphrine, Synephrine HCl, Synéphrine HCl, Synephrine Hydrochloride, Zeste d'Orange Amère, Zhi Ke, Zhi Qiao, Zhi Shi.)
74. Black Cohosh (Scientific name: Actaea racemosa, synonym Cimicifuga racemosa; Actaea macrotys. Also known as: Actée à Grappes, Actée à Grappes Noires, Actée Noire, Aristolochiaceae Noire, Baie d'actée, Baneberry, Black Aristolochiaceae, Black Snakeroot, Bugbane, Bugwort, Cimicaire à grappes, Cimicifuga, Cimicifuge, Cohosh Negro, Cohosh Noir, Cytise, Herbe aux Punaises, Macrotys, Phytoestrogen, Phytoestrogène, Racine de Serpent, Racine de Squaw, Racine Noire de Serpents, Rattle Root, Rattle Top, Rattlesnake Root, Rattleweed, Rhizoma Cimicifugae, Sheng Ma, Snakeroot, Squaw Root.)
75. Black Pepper (Scientific name: Piper nigrum. Also known as: Black Peppercorn, Extrait de Poivre, Grain de Poivre, Hu Jiao, Kali Mirchi, Kosho, Marich, Maricha, Miris, Peber, Peper, Pepe, Peppar, Pepper, Pepper Extract, Peppercorn, Pfeffer, Pimenta, Pimienta, Pimienta Negra, Pipar, Piper, Piperine, Pippuri, Poivre, Poivre Noir, Poivrier, Vellaja.)
76. Black Seed (Scientific name: Nigella sative. Also known as: Ajenuz, Aranuel, Baraka, Black Caraway, Black Cumin, Black Cumin Seed Oil, Charnuska, Cheveux de Vénus, Cominho Negro, Comino Negro, Cumin Noir, Cyah Dane, Fennel Flower, Fitch, Graine de Nigelle, Graine Noire, Habatul Sauda, Habbatul Baraka, Kalajaji, Kalajira, Kalonji, Ketsah, La Grainer Noire, Love in a Mist, Mugrela, Nielle, Nigelle de Crête, Nigelle Cultivée, Nutmeg Flower, Poivrette, Roman-Coriander, Schwarzkummel, Seed of Blessing, Siyah Dane, Shoniz, Small Fennel, Toute Épice, Upakuncika.)
77. Blueberry (Scientific name: Vaccinium angustifolium, synonyms Vaccinium brittonii, Vaccinium lamarckii, Vaccinium pensylvanicum; Vaccinium virgatum, synonyms Vaccinium ashei, Vaccinium amoenum; Vaccinium corymbosum, synonym Vaccinium constablaei; Vaccinium pallidum, synonyms Vaccinium altomontanum, Vaccinium vacillans. Also known as: Arándano, Bleuet, Bleuet des Champs, Bleuet des Montagnes, Bleuets, Blueberries, Highbush Blueberry, Hillside Blueberry, Lowbush Blueberry, Myrtille, Rabbiteye Blueberry, Rubel, Tifblue.)
78. Bobath
79. Body Control (Also known as: Body Manipulation, Body Electronics, Bodywork.)
80. Bone Setting
81. Borage (Scientific name: Borago officinalis. Also known as: Bee Plant, Beebread, Borage Oil, Borage Seed Oil, Borago, Borraja, Bourrache, Bourrache Commune, Burage, Burrage, Common Borage, Common Bugloss, Cool Tankard, Feuille de Bourrache, Fleur de Bourrache, Huile de Bourrache, Huile de Graines de Bourrache, Langue de Bœuf, Ox's Tongue, Pain-des-Abeilles, Starflower, Starflower Oil, Talewort.)
82. Boron (Scientific name: B, Atomic number 5. Also known as: Acide Borique, Anhydride Borique, B (symbole chimique), Borate, Borate de Sodium, Borates, Bore, Boric Acid, Boric Anhydride, Boric Tartrate, Boro, Numéro Atomique 5, Sodium Borate.)
83. Botanicals
84. Bowen Technique (Also known as: Bowen Manipulative Therapy, Bowen Therapist, Bowen Therapy, BT, Méthode Bowen, Técnica Bowen, Technique Bowen, Technique de Bowen, Thérapeute en Bowen, Thérapie Manuelle.)
85. Bromelain (Scientific name: Ananas comosus (Pineapple), synonyms Ananas ananas, Ananas duckei, Ananas sativus, Bromelia ananas, Bromelia comosa. Also known as: Ananas, Bromelaine, Bromélaïne, Bromelains, Bromelainum, Bromelin, Bromelina, Broméline, Concentré de Protéase Végétale, Enzyme d'Ananas, Extrait d'Ananas, Pineapple Enzyme, Pineapple Extract, Fruit Bromelain, Plant Protease Concentrate, Protease.)
86. Brown Peterson Technique
87. Buddhist Tantric Practice
88. Burdock (Scientific name: Arctium lappa, Arctium minus, Arctium tomentosum. Also known as: Arctium, Bardana, Bardana-Minor, Bardanae Radix, Bardane, Bardane Comestible, Bardane Géante, Bardane Majeure, Beggar's Buttons, Burdock Root Extract, Burr Seed, Clotbur, Cocklebur, Cockle Buttons, Edible Burdock, Fox's Clote, Gobo, Glouteron, Grande Bardane, Great Bur, Great Burdocks, Happy Major, Hardock, Harebur, Herbe aux Teigneux, Herbe du Teigneux, Lappa, Love Leaves, Niubang, Niu Bang Zi, Orelha-de-Gigante, Personata, Philanthropium, Rhubarbe du Diable, Thorny Burr.)
89. Buteyko Breathing Technique (Also known as: Buteyko Breathing Training, Buteyko Institute Method, Buteyko Method, Buteyko Technique, Eucapnic Breathing Technique, Eucapnic Buteyko Breathing.)
90. Butterbur (Scientific name: Petasites hybridus, synonyms Petasites officinalis, Tussilago hybrida. Also known as: Blatterdock, Bog Rhubarb, Bogshorns, Butter Bur, Butter-Dock, Butterfly Dock, Capdockin, Chapelière, Common Butterbur, Contre-Peste, Exwort, Feuille de Pétasite, Flapperdock, Fleur de Pétasite, Grand Bonnet, Herbe à la Peste, Herbe aux Teigneux, Langwort, Pestwurz, Pétasite, Pétasite Hybride, Pétasite Officinal, Pétasite Vulgaire, Petasites, Petasites Vulgaris, Petasitidis Folium, Petasitidis Hybridus, Petasitidis Rhizoma, Plague Root, Purple Butterbur, Racine de Pétasite, Rhizome de Pétasite, Umbrella Leaves.)
91. Bwiti
92. Calcium (Scientific name: Ca, Atomic number 20. Also known as: Acétate de Calcium, Aspartate de Calcium, Bone Meal, Calcarea Carbonica, Calcarea Phosphorica, Calcio,Calcium Acetate, Calcium Aspartate, Calcium Carbonate, Calcium Chelate, Calcium Chloride, Calcium Citrate, Calcium D-Gluconate, Calcium Disuccinate, Calcium Glucoheptonate, Calcium Gluconate, Calcium Glycerophosphate, Calcium HVPChelate, Calcium Hydrogen Phosphate, Calcium Hydroxyapatite, Calcium Lactate, Calcium Lactogluconate, Calcium Orotate,Calcium Oxide, Calcium Phosphate, Calcium Sulfate, Carbonate de Calcium, Chélate de Calcium, Chlorure de Calcium, Citratede Calcium, Citrate Malate de Calcium, Coquilles d'Huîtres Moulues, Coquilles d'oeuf, Di-Calcium Phosphate, Dolomite, EggShell Calcium, Gluconate de Calcium, Glycérophosphate de Calcium, Heated Oyster Shell-Seaweed Calcium, Hydroxyapatite,Lactate de Calcium, Lactogluconate de Calcium, MCHA, MCHC, Microcrystalline Hydroxyapatite, Orotate de Calcium, OsseinHydroxyapatite, Oyster Shell, Oyster Shell Calcium, Phosphate de Calcium, Phosphate de Calcium Hydrogène, Phosphate de di-Calcium, Phosphate Tricalcium, Poudre d'os, Sulfate de Calcium, Tricalcium Phosphate.)
93. Calendula (Scientific name: Calendula officinalis. Also known as: Caléndula, Calendule, English Garden Marigold, Fleur de Calendule, Fleur de Tous les Mois, Garden Marigold, Gold-Bloom, Holligold, Marigold, Marybud, Pot Marigold, Souci des Champs, Souci des Jardins, Souci des Vignes, Souci Officinal, Zergul.)
94. Calorie Control
95. Cannabis and Cannabinoids
a. Cannabis (Scientific name: Cannabis sativa. Also known as: Anashca, Banji, Bhang, Blunt, Bud, Cannabis, Charas, Dope, Esrar, Gaga, Ganga, Grass, Haschisch, Hash, Hashish, Herbe, Huo Ma Ren, Joint, Kif, Marie-Jeanne, Mariguana, Marihuana, Marijuana, Marijuana Médicale, Mary Jane, Medical Marijuana, Pot, Sawi, Sinsemilla, Weed
b. Cannabinoids (Cannabidiol (CBD), Tetrahydrocannabinol (THC))
96. Capsaicin
97. Capsicum (Scientific name: Capsicum frutescens, Capsicum annuum, Capsicum chinense, Capsicum baccatum, Capsicum pubescens, Capsicum minimum, and other Capsicum species. Also known as: African Bird Pepper, African Chillies, African Pepper, Aji, Bird Pepper, Capsaicin, Capsaïcine, Capsicum Fruit, Capsicum Oleoresin, Cayenne, Cayenne Fruit, Cayenne Pepper, Chili, Chili Pepper, Chilli, Chillies, Cis-capsaicin, Civamide, Garden Pepper, Goat's Pod, Grains of Paradise, Green Chili Pepper, Green Pepper, Hot Pepper, Hungarian Pepper, Ici Fructus, Katuvira, Lal Mirchi, Louisiana Long Pepper, Louisiana Sport Pepper, Mexican Chilies, Mirchi, Oleoresin Capsicum, Paprika, Paprika de Hongrie, Pili-pili, Piment de Cayenne, Piment Enragé, Piment Fort, Piment-oiseau, Pimento, Poivre de Cayenne, Poivre de Zanzibar, Poivre Rouge, Red Pepper, Sweet Pepper, Tabasco Pepper, Trans-Capsaicin, Zanzibar Pepper, Zucapsaicin, Zucapsaïcine.)
98. Caraway (Scientific name: Carum carvi, synonym Carum velenovskyi. Also known as: Alcaravea, Anis Canadien, Anis des Prés, Anis des Vosges, Apium Carvi, Carraway, Carvi, Carvi Commun, Carvi Fructus, Cumin des Montagnes, Cumin des Prés, Faux Anis, Haravi, Jeera, Jira, Kala Jira, Karwiya, Krishan Jeeraka, Krishnajiraka, Kummel, Kummich, Roman Cumin, Semen Cumini Pratensis, Semences de Carvi, Shahijra, Shiajira, Wiesen-Feldkummel, Wild Cumin.)
99. Carbohydrate Restricted Diet
100. Carnitine
a. L-Carnitine (Scientific name: 3-carboxy-2-hydroxy-N,N,N-trimethyl-1-propanaminium inner salt, (3-carboxy2-hydroxypropyl) trimethylammonium hydroxide inner salt, B-hydroxy-Ntrimethyl aminobutyric acid, Beta-hydroxy-gammatrimethylammonium butyrate, L-3-hydroxy-4-(trimethylammonium)-butyrate, (R)-(3-carboxy-2 hydroxypropyl)trimethylammonium hydroxide, (R)-3-hydroxy-4-trimethylammonio-butyrate, 3-hydroxy-4-Ntrimethylaminobutyrate. Also known as: Aminocarnitine, Beta-hydroxyl-gamma-tributyl aminobutyrate, B(t) Factor, Carnitine, Carnitor, D-Carnitine, DLCarnitine, Facteur B(t), L-b-hydroxy-c-N-trimethylaminobutyric acid, L-Carnitina, L-Carnitine Fumarate, L-Carnitine L-Tartrate, L-Carnitine Tartrate, Levocarnitine, Lévocarnitine, Levocarnitine Fumurate, Vitacarn, Vitamin B(t), Vitamine B(t).)
b. Acetyl-L-Carnitine (Scientific name: 2-(acetyloxy)-3-carboxy-N,N,N-trimethyl-1-propanaminium inner salt; (3-carboxy-2-hydroxypropyl)trimethylammonium hydroxide inner salt acetate. Also known as: Acetil-L-Carnitina, Acetyl Carnitine, Acétyl Carnitine, Acetyl L-Carnitine, Acétyl-L-Carnitine, Acetyl-L-Carnitine Arginate Dihydrochloride, Acetyl-L-Carnitine Arginate HCl, Acétyl-L-Carnitine Arginate HCl, Acetyl-L-Carnitine HCl, Acétyl-LCarnitine HCl, Acetyl L-Carnitine Hydrochloride, Acetyl Carnitine, Acetyl-Carnitine, Acetyl-Levocarnitine, Acétyl-Lévocarnitine, ALC, ALCAR, Aminocarnitine, Carnitine Acetyl Ester, Dihydrochlorure d'Acétyl-L-Carnitine Arginate, Gamma-Trimethyl-BetaAcetylbutyrobetaine, L-Acetylcarnitine, L-Acétylcarnitine, Levacecarnine, N-Acetyl-Carnitine, N-Acétyl-Carnitine, N-AcetylCarnitine Hydrochloride, N-Acetyl-L-Carnitine, N-Acétyl-L-Carnitine, ST-200, Vitamin B(t) Acetate.)
101. Cartilage Supplement
a. Shark Cartilage (Scientific name: Squalus acanthias (Spiny Dogsh Shark); Sphyrna lewini (Scalloped Hammerhead Shark). Also known as: AE-941, Cartilage de Requin, Cartilage de Requin du Pacique, Cartilago de Tiburon, Collagène Marin, Extrait de Cartilage de Requin, Liquide de Cartilage Marin, Marine Collagen, Marine Liquid Cartilage, MSI-1256F, Neovastat, Pacic Shark Cartilage, Poudre de Cartilage de Requin, Shark Cartilage Extract, Shark Cartilage Powder.)
b. Bovine Cartilage (Also known as: Antitumor Angiogenesis Factor (Anti-TAF), Bovine Tracheal Cartilage (BTC), Cartilage Trachéal de Bovins, Cartílago Bovino, Catrix, Catrix-S, Collagen Bovine, Collagène Bovin, Glycosaminoglycan Polysulphuric Acid Complex, Processed Bovine Cartilage, Psoriacin, Psoriacin-T, Rumalon.)
102. Casein Free Diet
103. Catgut Implantation
104. Cat's Claw (Scientific name: Uncaria guianensis, Uncaria tomentosa. Also known as: Griffe Du Chat, Liane du Pérou, Life-Giving Vine of Peru, Peruvian Liana, Samento, Uña De Gato.)
105. Cellasene
106. Cetyl Myristoleate
107. Chamomile
a. German Chamomile (Scientific name: Matricaria recutita, synonyms Chamomilla recutita, Matricaria chamomilla. Also known as: Blue Chamomile, Camomèle, Camomilla, Camomille, Camomille Allemande, Camomille Sauvage, Camomille Tronquée, Camomille Vraie, Chamomile, Chamomilla, Echte Kamille, Feldkamille, Fleur de Camomile, Hungarian Chamomile, Kamillen, Kleine Kamille, Manzanilla, Manzanilla Alemana, Matricaire, Matricaire Camomille, Matricariae Flos, Œil du Soleil, Petite Camomille, Pin Heads, Sweet False Chamomile, True Chamomile, Wild Chamomile.)
b. Roman Chamomile (Scientific name: Chamaemelum nobile, synonyms Anthemis nobilis, Ormenis nobilis. Also known as: Anthémis, Anthémis Odorante, Anthemis nobilis, Babounge, Babuna Ke Phool, Camomille d'Anjou, Camomille Noble, Camomille Romaine, Chamaemelum nobile, Chamomile, Chamomilla, Chamomillae Ramane Flos, English Chamomile, Fleur de Camomille Romaine, Flores Anthemidis, Garden Chamomile, Grosse Kamille, Ground Apple, Huile Essentielle de Camomille Romaine, Low Chamomile, Manzanilla, Manzanilla Romana, Ormenis nobilis, Roman Chamomile Essential Oil, Römische Kamille, Sweet Chamomile, Whig Plant.)
108. Chanting
109. Cheirology
110. Chelated Minerals (Scientific name: Mineral-Amino Acid Complex. Also known as: Bore Chélaté, Calcium Chélaté, Chelated Boron, Chelated Calcium, Chelated Chromium, Chelated Cobalt, Chelated Copper, Chelated Iron, Chelated Magnesium, Chelated Manganese, Chelated Molybdenum, Chelated Potassium, Chelated Selenium, Chelated Trace Minerals, Chelated Vanadium, Chelated Zinc, Chrome Chélaté, Cobalt Chélaté, Cuivre Chélaté, Fer Chélaté, Magnésium Chélaté, Manganèse Chélaté, Minerales Quelados, Minéraux Chélatés, Molybdène Chélaté, Potassium Chélaté, Sélénium Chélaté, Vanadium Chélaté, Zinc Chélaté.)
111. Chiropractic (Also known as: Chiro Therapy, Chirotherapy, Chiropractic Physician, Chiropraticien, Chiropractie, Chiropractors, Chiropratique, Chiropraxie, Manipulation Rachidienne, Manipulation Vertébrale, Manipulative Therapy, Physical Medicine, Quiropráctica, SMT, Spinal Manipulative Therapy, Subluxation.)
112. Chitosan (Scientific name: Chitosan. Also known as: Ascorbate de Chitosane, Chitosan Ascorbate, Chitosane, Chitosane Déacétylé, Chitosane Mono-Carboxyméthylé, Chitosan-N-Acetylcysteine, Deacetylated Chitin, Deacetylated Chitosan, Enzymatic Polychitosamine Hydrolisat, HEP-30, Hydrolisat Enzymatique de Polychitosamine, Mono-Carboxymethylated Chitosan, N-Carboxybutyl Chitosan, N-Carboxybutyl Chitosane, N,O-Sulfated Chitosan, O-Sulfated N-Acetylchitosan, Poly-D-Glucosamine, Poly-N-Acetyl-Glucosamine, Quitosano, Sulfated N-Carboxymethylchitosan, Sulfated O-Carboxymethylchitosan, Trimethyl Chitosan Chloride.)
113. Choline (Scientific name: Trimethylethanolamine, (beta-hydroxyethyl) trimethylammonium hydroxide. Also known as: Bitartre de Choline, Chlorure de Choline, Choline Bitartrate, Choline Chloride, Choline Citrate, Citrate de Choline, Colina, Facteur Lipotropique, Hydroxyde de Triméthylammonium (bêta-hydroxyéthyl), Intrachol, L-Choline, Lipotropic Factor, Methylated Phosphatidylethanolamine, Triméthyléthanolamine.)
114. Chondroitin Sulfate (Scientific name: Chondroitin 4-sulfate; Chondroitin 4- and 6-sulfate. Also known as: Calcium Chondroitin Sulfate, CDS, Chondroitin Polysulfate, Chondroitin Sodium Sulfate, Chondroitin Sulphate, Chondroïtine, Chondroïtine Sulfate A, Chondroïtine Sulfate B, Chondroïtine Sulfate C, Chondroïtine 4-Sulfate, Chondroïtine 4- et 6-Sulfate, Condroitin, CPS, CS, CSA, CSC, GAG, Galactosaminoglucuronoglycan Sulfate, Poly-(1- > 3)-N-Acetyl-2-Amino-2- Deoxy-3-O-Beta-D-Glucopyranurosyl-4-(or 6-), Polysulfate de Chondroïtine, Shark Chondroitin Sulphate, Sulfate de Chondroïtine, Sulfate de Galactosaminoglucuronoglycane, Sulfates de Chondroïtine, Sulfato de Condroitina.)
115. Chromium (Scientific name: Cr, Atomic number 24. Also known as: Acétate de Chrome, Chlorure Chromique, Chlorure de Chrome, Chrome, Chrome III, Chrome 3 + , Chrome FTG, Chrome Facteur de Tolérance au Glucose, Chrome Trivalent, Chromic Chloride, Chromium Acetate, Chromium Chloride,Chromium Nicotinate, Chromium Picolinate, Chromium Polynicotinate, Chromium Proteinate, Chromium Trichloride, Chromium Tripicolinate, Chromium III, Chromium III Picolinate, Chromium 3 + , Cr III, Cr3 + , Cromo, Glucose Tolerance Factor-Cr, GTF, GTF Chromium, GTF-Cr, Kali Bichromicum, Nicotinate de Chrome, Numéro Atomique 24, Picolinate de Chrome, Picolinate de Chrome III, Polynicotinate de Chrome, Potassium Bichromate, Protéinate de Chrome, Trichlorure de Chrome, Tripicolinate de Chrome, Trivalent Chromium.)
116. Chromotherapy (Also known as: Chromothérapeute, Chromotherapie, Chromothérapie, Chromotherapist, Color Medicine, Color Therapy, Colorologie, Colorology, Cromoterapia, Thérapie des Couleurs, Thérapie par les Couleurs.)
117. Chronotherapy
118. Cinnamon
a. Cassia cinnamon (Scientific name: Cinnamomum aromaticum, synonym Cinnamomum cassia. Also known as: Bastard Cinnamon, Canela de Cassia, Canela de la China, Canela Molida, Canelero Chino, Canelle, Cannelle Bâtarde, Cannelle Cassia, Cannelle de Ceylan, Cannelle de Chine, Cannelle de Cochinchine, Cannelle de Padang, Cannelle de Saigon, Cannelier Casse, Cannelier de Chine, Canton Cassia, Casse, Casse Odorante, Cassia, Cassia Aromaticum, Cassia Bark, Cassia Lignea, Chinazimt, Chinese Cassia, Chinese Cinnamon, Chinesischer Zimtbaum, Cinnamomi Cassiae Cortex, Cinnamomum, Cinnamon, Cinnamon Essential Oil, Cinnamon Flos, Cinnamoni Cortex, Cinnamonomi Cortex, Cortex Cinnamomi, Écorce de Cassia, False Cinnamon, Fausse Cannelle, Gui Zhi, Huile Essentielle de Cannelle, Kassiakanel, Keishi, Laurier des Indes, Nees, Ramulus Cinnamomi, Rou Gui, Sthula Tvak, Taja, Zimbluten, Zimtcassie.)
b. Ceylon Cinnamon (Scientific name: Cinnamomum verum, synonyms Cinnamomum zeylanicum, Laurus cinnamomum. Also known as: Batavia Cassia, Batavia Cinnamon, Canela, Canelero de Ceilán, Cannelier de Ceylan, Cannelle de Ceylan, Cannelle de Saïgon, Cannelle du Sri Lanka, Ceylonzimt, Ceylonzimtbaum, Cinnamon Bark, Corteza de Canela, Dalchini, Écorce de Cannelle, Echter Ceylonzimt, Madagascar Cinnamon, Sri Lanka Cinnamon, Thwak, True Cinnamon, Tvak, Xi Lan Rou Gui, Zimtbaum.)
c. Saigon Cinnamon (Scientific name: Cinnamomum loureirii, synonym Cinnamomum loureiroi. Also known as: Baker's Cinnamon, Canela de Saigón, Nikkei, Nhucgue, Que Thanh, Saigon Cassia, Saigonkanel, Saigonzimt, Saigonzimtbaum, Vietnamese Cassia, Vietnamese Cinnamon, Yukgyenamu.)
d. Padang Cassia (Scientific name: Cinnamomum burmannii. Also known as: Batavia cassia, Batavia Cinnamon, Birmazimt, Birmazimtbaum, Canelle de Padang, Cannelier de Malaisie, Cassia Vera, Cinnamon Stick, Fagot Cassia, Indonesian Cassia, Indonesian Cinnamon, Indonesische Kaneel, Indonesischer Zimt, Jaavakaneli, Java Cassia, Java Cinnamon, Kayo Manis Padang, Kayu Manis Padang, Korintje, Korintje Cassia, Korintje Cinnamon, Padang Cassia, Padang Cinnamon, Padang Zimt, Padangzimt, Padangzimtbaum, Timor Cassia.)
e. Indian Cassia (Scientific name: Cinnamomum tamala. Also known as: Chai Gui, Indian Bay Leaf, Indian Bark, Indian Cassia, Malobathrum, Talish Pattri, Tamala, Tamala Patar, Tamala Patra, Tamalpatra, Tejpat, Tejpat Oil, Tejpata, Tejpatra, Tejpatta, Tez Pat, Tezpat.)
119. Cleansing
120. Climatotherapy
121. Clinical Ecology
122. Clove (Scientific name: Syzygium aromaticum. Also known as: Bourgeon Floral de Clou de Girofle, Bouton Floral de Clou de Girofle, Caryophylli Flos, Caryophyllum, Clavo de Olor, Clous de Girolfe, Clove Flower, Clove Flowerbud, Clove Leaf, Clove Oil, Clove Stem, Cloves, Cloves Bud, Ding Xiang, Feuille de Clou de Girofle, Fleur de Clou de Girofle, Flores Caryophylli, Gewurznelken Nagelein, Girofle, Giroflier, Huile de Clou de Girofle, Kreteks, Lavang, Lavanga, Oil of Clove, Tige de Clou de Girofle.)
123. Coenzyme Q10 (Scientific name: Ubiquinol, Ubiquinone, Ubidecarenone, Mitoquinone. Also known as: Coenzima Q-10, Coenzyme Q-10, CoQ10, Ubidécarénone, Ubiquinone-10.)
124. Coffee (Scientific name: Coffea arabica, Coffea canephora, synonyms Coffearobusta, Coffea bukobensis; Coffea liberica, synonym Coffea arnoldiana; other Coffea species. Also known as: Cafe, Café, Café Arabica, Café Robusta, Coffea Cruda, Espresso, Expresso, Java, Mocha.)
125. Cognitive Therapy
126. Coley's Toxin (Also known as: Mixed Bacterial Vaccine.)
127. Colloidal Silver (Scientific name: Silver in Suspending Agent. Also known as: Argent Colloïdal, Argent Ionique, Argent Natif, Argentum Metallicum, Colloidal Silver Protein, Ionic Silver, Native Silver, Plata Coloidal, Protéine d'Argent, Silver, Silver Alginate, Silver Protein, Tetrasilver Tetroxide, Tétroxyde de Tétra-Argent.)
128. Complementary Medicine (Also known as: Complementary Therapy, Complementary Approach, Complementary Health.)
129. Conjugated Linoleic Acid (CLA) (Scientific name: cis-9,trans-11 conjugated linoleic acid; Trans-10,cis-12 conjugated linoleic acid. Also known as: Acide Linoléique Conjugué, Acide Linoléique Conjugué Cis-9,trans-11, Acide Linoléique Conjugué trans-10,cis12, Acido Linoleico Conjugado, ALC, Cis-Linoleic Acid, CLA, CLA-Free Fatty Acid, CLA-Triacylglycerol, LA, Linoleic Acid.)
130. Contact Reflex Analysis
131. Copper (Scientific name: Cu, Atomic number 29. Also known as: Citrate de Cuivre, Cobre, Copper Citrate, Copper Gluconate, Copper Sulfate, Cuivre, Cuivre Élémentaire, CupricOxide, Cupric Sulfate, Cupric Sulfate Pentahydrate, Cuprum Aceticum, Cuprum Metallicum, Elemental Copper, Gluconate deCuivre, Numéro Atomique 29, Oxyde Cuivrique, Pentahydrate de Sulfate de Cuivre, Sulfate de Cuivre, Sulfate Cuivrique, Sulfate Cuprique.)
132. Copper Wrist Band
133. Cordyceps (Scientific name: Ophiocordyceps sinensis, synonyms Cordyceps sinensis. Also known as: Caterpillar Fungus, Caterpillar Mushroom, Champignon Chenille, Chinese Caterpillar Fungus, Cs-4, Dong Chong Xia Cao, Dong Chong Zia Cao, Hsia Ts'Ao Tung Ch'Ung, Jinshuibao Jiaonang, Jinshuibao Pian, Tochukaso, Vegetable Caterpillar, Winter Worm Summer Grass.)
134. Coriolus Mushroom (Scientific name: Coriolus versicolor, synonyms Trametes versicolor, Polyporus versicolor, Boletus versicolor, Polystictus versicolor. Also known as: Bolet à Couleurs Variées, Bolet Versicolore, Champignon Coriolus, Champignon de Queue de Dinde, Cloud Mushroom, Coriolus, Hongo Coriolus, Kawaratake, Krestin, Polypore à Couleurs Variées, Polypore Versicolor, PolysaccharideK, Polysaccharide Krestin, Polysaccharide Peptide, Polysaccharopeptide, PSK, PSP, Turkey Tail Mushroom, Yun Chi, Yun Zhi, Yunzhi, Yun-Zhi.)
135. Counseling
136. Cranberry (Scientific name: Vaccinium macrocarpon, synonym Oxycoccus macrocarpos; Vaccinium oxycoccos, synonyms Oxycoccus hagerupii, Oxycoccus microcarpus, Oxycoccus palustris, Oxycoccus quadripetalus, Vaccinium hagerupii, Vaccinium microcarpum, Vaccinium palustre. Also known as: Agrio, Airelle à Gros Fruits, Airelle Canneberge, Airelle Européenne, Airelle Rouge, American Cranberry, Arándano, Arándano Americano, Arándano Rojo, Arándano Trepador, Atoca, Atoka, Bearberry, Canneberge, Canneberge à Feuillage Persistant, Canneberge d'Amérique, Canneberge Européenne, Cocktail au Jus de Canneberge, Cranberry Extract, Cranberry Fruit, Cranberry Fruit Juice, Cranberry Juice, Cranberry Juice Cocktail, Cranberry Juice Concentrate, Cranberry Powder, Cranberry Powdered Extract, Craneberry, Da Guo Yue Jie, Da Guo Yue Ju, Da Guo Suan Guo Man Yue Ju, European Cranberry, Extrait de Canneberge, Große Moosbeere, Gros Atoca, Grosse Moosbeere, Jus de Canneberge, Jus de Canneberge à Base de Concentré, Jus de Canneberge Frais, Kliukva, Kliukva Obyknovennaia, Kranbeere, Large Cranberry, Man Yue Ju, Man Yue Mei, Moosebeere, Mossberry, Oomi No Tsuruko Kemomo, Petite Cannberge, Pois de Fagne, Pomme des Prés, Ronce d'Amerique, Sirop de Canneberge, Small Cranberry, Trailing Swamp Cranberry, Tsuru-Kokemomo.)
137. Craniosacral Therapy (Also known as: Approche Craniosacrale, Cranial Osteopathy, Cranial Therapy, Cranio-Occipital Technique, Craniosacral, Craniosacral Approach, Craniosacral Bodywork, Craniosacral Rhythm, Craniosacral System, Craniosacral Treatment, Craniosacrale Thérapie, CST, Ostéopathie Crânienne, Rythme Craniosacral, Système Craniosacral, Terapia Craneosacral, Thérapie Crânienne, Thérapie Craniosacrale, Thérapie Cranio-sacrée, Thérapie Cranio-sacrée Upledger, Traitement Craniosacral, Upledger Therapy.)
138. Crystal Therapy (Also known as: Cristaux, Crystal Healing, Crystals, Electrocrystal Therapy, Gem Therapy, Gemstone Therapy, Guérison par les Pierres, Lithothérapie, Pierre Précieuse, Terapia de Cristal, Thérapie par les Cristaux, Thérapie par les Pierres.)
139. Cumin (Scientific name: Cuminum cyminum, synonym Cuminum odorum. Also known as: Anis Âcre, Comino, Cumin de Malte, Cummin, Green Cumin, Jeeraka, Svetajiraka, Zira.)
140. Cupping (Also known as: Bleeding Cupping, Blood-Letting Cupping, Cupping Massage, Dry Cupping, Fire-Cupping, Flash Cupping, Hijamat, Liquid Cupping, Medicinal Cupping, Momentary Cupping, Moving Cupping, Multiple Cupping, Needle Cupping, Pneumatic Pulsation Therapy, Pulsating Cupping, Retained Cupping, Shuiguanfa, Traditional Cupping, Ventosaterapia, Ventousothérapie, Water Cupping, Wet Cupping, Wet-Cupping.)
141. Curanderismo (Also known as: Curandera, Curandero, Folk Healers, Latin American Folk Medicine, Latin American Healing, Mexican American Healing Tradition.)
142. Curcumin
143. Current Therapy
144. Cymatic Therapy
145. Dance Therapy
146. Dandelion (Scientific name: Taraxacum officinale, synonyms Taraxacum vulgare, Leontodon taraxacum, Taraxacum dens-leonis, Taraxacum mongolicum, Taraxacum sinicum, Taraxacum laevigatum. Also known as: Blowball, Cankerwort, Cochet, Common Dandelion, Couronne de Moine, Délice Printanier, Dandelion Extract, Dent-de-Lion, Diente de Leon, Dudal, Endive Sauvage, Fausse Chicorée, Florin d'Or, Florion d'Or, Ghasedak, Herba Taraxaci, Laitue de Chien, Lion's Teeth, Lion's Tooth, Piss-A-Bed, Pisse au Lit, Pissenlit, Pissenlit Vulgaire, Priest's Crown, Pu Gong Ying, Red-Seed Dandelion, Salade de Taupe, Swine Snout, Taraxaci Herba, Taraxacum, Tête de Moine, Wild Endive.)
147. Danshen (Scientific name: Salvia bowleyana, Salvia miltiorrhiza, Salvia przewalskii, Salvia yunnanensis. Also known as: Ch'ih Shen, Chinese Red Sage, Chinese Sage, Chinese Salvia, Dan Shen, Dan-Shen, Huang Ken, Pin-Ma Ts'ao, Racine de Salvia, Radix Salviae Miltiorrhizae, Radix Salvie Miltiorrhiae, Red Root Sage, Red Rooted Sage, Red Sage, Salvia Przewalskii Mandarinorum, Salvia Root, Sage Miltiorrhiza, Salviae Miltiorrhizae, Sauge Rouge, Sauge Rouge Chinoise, Shu-Wei Ts'ao, Tan Seng, Tan-Shen, Tzu Tan-Ken.)
148. Dehydroepiandrosterone
149. Dengzhanhua Preparation
150. Dervish Dance (Also known as: Sufi Whirling, Sufi Dance.)
151. Detoxification (Also known as: Aqua Detox, Body Detox, Coffee Enema, Colon Cleanse, Colon Hydrotherapy, Enema, Desintoxicación, Detox, Detox Diet, Detox Treatment, Détoxification, Diet Detox, Fad Diet, Gallbladder Cleanse, Gallbladder Flushing, Herbal Cleansing, Herbal Detox, Hydrothérapie du Côlon, Jeûne de Jus de Fruits, Juice Fasting, Lavement, Lavement de Café, Liver Cleanse, Liver Flushing, Nettoyage à Base de Plantes, Nettoyage du Côlon, Purification Rundown, Régime Détox, Starvation Diet, Traitement de Détoxfiication, Water Diet, Water Fasting.)
152. Devil's Claw (Scientific name: Harpagophytum procumbens, synonym Uncaria procumbens; Harpagophytum zeyheri. Also known as: Devils Claw, Devil's Claw Root, Garra del Diablo, Grapple Plant, Griffe du Diable, Harpagophyti Radix, Harpagophytum, Racine de Griffe du Diable, Racine de Windhoek, Teufelskrallenwurzel, Wood Spider.)
153. Devil's Club (Scientific name: Oplopanax horridus; synonyms Echinopanax horridus, Fatsia horrida. Also known as: Alaskan Ginseng, Bois Piquant, Cukilanarpak, Devils Club, Devil's Root, Fatsia, Garrote del Diablo, Panax Horridum.)
154. Diamond Diet
155. Dianxianning Pill
156. Diathermy
157. Diet Therapy
158. Dihomogammalinolenic Acid
159. Dimethylaminoethanol (Also known as: Dimethylethanolamine, Deanol.)
160. Dimethylsulfoxide (DMSO) (Also known as: Dimethyl Sulfoxide, Dimethyl Sulphoxide, Dimethylis Sulfoxidum, Diméthylsulfoxyde, Dimetilsulfóxido, MethylSulphoxide, NSC-763, SQ-9453, Sulfoxyde de Diméthyl, Sulphinybismethane.)
161. DMPS Chelation Therapy (Also known as: 2,3-dimercaptopropane-1-sulfonate, Dimaval, DMPS, Chelation Therapy.)
162. Docosahexaenoic acid (DHA) (Scientific name: Docosahexaenoic Acid. Also known as: Acide Docosahexaénoïque, Acide Gras d'Huile de Poisson, Acide Gras Oméga 3, Acide Gras N-3, Acide Gras W-3, Acido Docosahexaenoico, ADH, DHA, Fish Oil Fatty Acid, N-3 Fatty Acid, Neuromins, Omega 3, Oméga 3, Omega 3 Fatty Acids, Omega-3, Omega-3 Fatty Acids, W-3 Fatty Acid.)
163. Doman Delacato Patterning Therapy
164. Dong Quai (Scientific name: Angelica sinensis, synonym Angelica polymorpha var. sinensis. Also known as: Angelica China, Angelicae Gigantis Radix, Angélique Chinoise, Angélique de Chine, Chinese Angelica, Dang Gui, Danggui, Danguia, Dang Gui Shen, Dang Gui Tou, Dang Gui Wei, Don Quai, Kinesisk Kvan, Ligustilides, Phytoestrogen, Radix Angelicae Gigantis, Radix Angelicae Sinensis, Tan Kue Bai Zhi, Tang Kuei, Tanggwi, Toki.)
165. Drama Therapy
166. Dream Therapy
167. Dukun
168. Ear Candling (Also known as: Auricular Candles, Auriculothérapie, Bougie Creuse, Bougie Hopi, Bougie d'Oreille, Chandelles Auriculaires, Cônage d'Oreille, Ear Candle, Ear Candle Therapy, Ear Candle Treatment, Ear Coning, Terapia de Vela en el Oído, Thérapie Auriculaire Thermale, Thermal-Auricular Therapy.)
169. Echinacea (Scientific name: Echinacea angustifolia, synonym Brauneria angustifolia; Echinacea pallida, synonyms Brauneria pallida, Rudbeckia pallida, Echinacea purpurea, synonyms Brauneria purpurea, Echinacea intermedia, Echinacea serotina, Echinacea speciosa, Helichroa purpurea, Rudbeckia purpurea. Also known as: American Cone Flower, Black Sampson, Black Susans, Comb Flower, Coneflower, Echinaceawurzel, Échinacée, Échinacée Angustifolia, Échinacée Pallida, Échinacée Pourpre, Échinacée Purpurea, Equinácea, Fleur À Hérisson, Hedgehog, Igelkopfwurzel, Indian Head, Kansas Snakeroot, Narrow-Leaved Echinacea, Narrow-Leaved Purple Coneflower, Narrow-leaved Purple Cone Flower, Pale Coneflower, Pale Flower Echinacea, Pale Purple Coneflower, Purple Cone flower, Purple Cone Flower, Purpursonnenhutkraut, Purpursonnenhutwurzel, Racine D'echininacea, Red Sunflower, Rock-Up-Hat, Roter Sonnenhut, Rudbeckie Pourpre, Schmallblaettrige Kegelblumenwurzel, Schmallblaettriger Sonnenhut, Scurvy Root, Snakeroot, Sonnenhutwurzel.)
170. Eclectic Medicine
171. Edetic Acid (Also known as: Edetate.)
172. Effleurage
173. Eicosapentaenoic Acid (Scientific name: Eicosapentaenoic Acid. Also known as: 20:5n-3, Acide Eicosapentaénoïque, Acide Éthyle-Eicosapentaénoïque, Acide Gras Essentiel, Acide Gras d'Huile de Poisson, Acide Gras N-3, Acide Gras Omega, Acide Gras Oméga 3, Acide Gras Polyinsaturé, Acide Gras W3, Acido Eicosapentaenoico, EPA, E-EPA, Eicosapentanoic Acid, Essential Fatty Acid, Ethyl Eicosapentaenoic Acid, Ethyl-Eicosapentaenoic Acid, Ethyl-EPA, Fish Oil Fatty Acid, Icosapent Ethyl, N-3 Fatty Acid, Omega Fatty Acid, Omega 3, Oméga 3, Omega 3 Fatty Acids, Omega-3, Omega-3 Fatty Acids, Polyunsaturated Fatty Acid, PUFA, W-3 Fatty Acid.)
174. Electrodermal Testing (Also known as: EDT, Vega Test, Wheatstone Bridge.)
175. Electrotherapy (Also known as: Electrostimulation.)
176. Elemental Diet
177. Ellagic Acid (Scientific name: Ellagic Acid. Also known as: 3,4,3',4'-Hydroxyl-Benzopyranol[5,4,3-c,d,e][1]Benzopyrn-6–6'-Dione, Acide Ellagique, Ácido Elágico, Gallogen.)
178. Emodin
179. Emotional Freedom Technique (Also known as: Body Tapping, EFT, Emotional Freedom Technique, Tapping.)
180. Energy Therapy (Also known as: Bioénergie, Biofield Therapies, Energy Healing, Energy Medicine, Putative Energy Therapy, Veritable Energy Therapy.)
181. Environmental Medicine
182. Enzyme Therapy (Also known as: Enzyme Replacement Therapy, Systemic Enzyme Therapy.)
183. Ephedra (Scientific name: Ephedra distachya, synonym Ephedra vulgaris; Ephedra equisetina, synonym Ephedra shennungiana; Ephedra gerardiana; Ephedra intermedia; Ephedra sinica; Ephedra sinensis; and other Ephedra species. Also known as: Alcaloïde d'Éphédrine, Belcho, Cao Mahuang, Chinese Ephedra, Chinese Joint-Fir, Cao Ma-Huang, Desert Herb, Efedra, Éphédra, Éphédra Américain, Éphédra Chinoise, Éphédra Européen, Ephedra Sinisa, Éphèdre, Ephedrine, Éphédrine, Ephedrine Alkaloid, Épitonin, Herbal Ecstasy, Indian Jointfir, Joint Fir, Ma Huang, Mahuanggen (Ma Huang Root), Mongolian Ephedra, Muzei Ma Huang, Pakistani Ephedra, Popotillo, Raisin de Mer, Sea Grape, Shuang Sui Ma Huang, Teamster's Tea, Thé de Désert, Yellow Astringent, Yellow Horse, Zhong Mahuang.)
184. Esoteric Therapy
185. Essaic
186. Essence Therapy
187. Estrogen Supplement
188. Ethnomedicine
189. Ethylenediaminetetraacetic Acid
190. Evening Primrose (Scientific name: Oenothera biennis, synonyms Oenothera muricata, Oenothera rubricaulis, Oenothera suaveolens, Onagra biennis. Also known as: Aceite de Onagra, Acide Cis-linoléique, Cis-Linoleic Acid, EPO, Evening Primrose Oil, Evening Primrose Seed Oil, Evening Star, Fever Plant, Herbe-aux-ânes, Huile de Graines d'Onagre, Huile D'Onagre, Huile de Primerose, Huile de Primevère Vespérale, Jambon de Jardinier, Jambon du Paysan, King's Cureall, Mâche Rouge, Night Willow-Herb, OEnothère, Oil of Evening Primrose, Onagraire, Onagre Bisannuelle, Onagre Commune, Primevère du Soir, Primrose, Primrose Oil, Scabish, Scurvish, Sun Drop, Tree Primrose.)
191. Exercise (Also known as: Physical Therapy.)
192. Experience Based Medicine
193. Eye Movement Desensitization and Reprocessing
194. Fasting (Also known as: Alternate-Day Fasting, Alternate-Day Modified Fasting, Caloric Restriction, Energy Restriction, IER, Intermittent Energy Restriction, Intermittent Fasting, Intermittent Severe Energy Restriction, Periodic Fasting, Ramadan Intermittent Fasting, Time-Restricted Feeding, Therapeutic Fasting, Total Caloric Desistance, Water-Only Fasting, Zero Calorie Diet.)
195. Fat-Restricted Diet
196. Fatty Acids
a. Cetylated Fatty Acids (CFAs) (Scientific name: Cetylated fatty acids. Also known as: Acides Gras Cetylated, Acides Gras Cétylés, Acides Gras Estérisés, Acides Gras Mono-Insaturés Cétylés, Ácidos Grasos Cetilados, Cerasomal-cis-9-cetylmyristoleate, Cetyl Decanoate, Cetyl Laurate, Cetyl Laureate, Cetyl Myristate, Cetyl Myristoleate, Cetyl Oleate, Cetyl Palmitate, Cetyl Palmitoleate, Cetyl Stearate, Cetylated Monounsaturated Fatty Acids, Cetylmyristoleate, CFA, Cis-9-cetylmyristoleate, CM, CMO, Esteried Fatty Acid Carbons, Esteried Fatty Acids, Lauréate Cétyl, Myristate Cétyl, Myristoléate Cétyl, Oléate Cétyl, Palmitate Cétyl, Palmitoléate Cétyl.)
b. Omega-6 Fatty Acids (Scientific name: Omega-6 Polyunsaturated Fatty Acids. Also known as: Acides Gras Essentiels N-6, Acides Gras Oméga-6, Acides Gras Omégas 6, Acides Gras Polyinsaturés, Acidos Grasos Omega 6, AGE, AGPI, Huiles d'Oméga 6, N-6 Essential Fatty Acids, Omega 6 Fatty Acids, Omega 6 Oils, Polyunsaturated Fatty Acids, PUFAs.)
197. Feldenkrais Method (Also known as: Awareness Through Movement, Feldenkrais Bodywork, Functional Integration, Gestalt Synergy.)
198. Feng Shui (Also known as: Feng-Shui, Fengshui, Foong Shway, Fung Shway, Fusui, Phong-Thuy, Pung-Su.)
199. Fenugreek (Scientific name: Trigonella foenum-graecum; Trigonella foenugraecum. Also known as: Alholva, Bird's Foot, Bockshornklee, Bockshornsame,
Chandrika, Egypt Fenugreek, Fenogreco, Fenugrec, Foenugraeci Semen, Foenugreek, Greek Clover, Greek Hay, Greek Hay Seed, Hu Lu Ba, Methi, Methika, Medhika, Senegrain, Senegre, Trigonella, Trigonella Foenum, Trigonelle, Woo Lu Bar.)
200. Feverfew (Scientific name: Tanacetum parthenium, synonyms Chrysanthemum parthenium, Chrysanthemum praealtum, Leucanthemum parthenium, Matricaria eximia, Matricaria parthenium, Pyrethrum parthenium. Also known as: Altamisa, Bachelor's Buttons, Chrysanthème Matricaire, Featerfoiul, Featherfew, Featherfoil, Flirtwort Midsummer Daisy, Grande Camomille, Matricaria, Partenelle, Pyrèthre Doré, Pyrèthre Mousse, Santa Maria, Tanaceti Parthenii, Tanaisie Commune.)
201. Finnish Sauna
202. Fish Oil (Also known as: Aceite de Pescado, Acides Gras Oméga-3, Acides Gras Oméga 3, Acides Gras Oméga 3 Sous Forme Ester Éthylique, Acides Gras N-3, Acides Gras Polyinsaturés N-3, Acides Gras W3, ACPI, EPA/DHA Ethyl Ester, Ester Éthylique de l'AEP/ADH, Herring Oil, Huile de Foie de Morue, Huile de Hareng, Huile de Menhaden, Huile de Poisson, Huile de Saumon, Huile de Thon, Huile Lipidique Marine, Huile Marine, Huiles Marines, Lipides Marins, Marine Lipid Concentrate, Marine Fish Oil, Marine Lipid Oil, Marine Lipids, Marine Oil, Marine Triglyceride, Menhaden Oil, N-3 Fatty Acids, N3-polyunsaturated Fatty Acids, Omega-3, Oméga-3, Omega-3 Fatty Acid Ethyl Ester, Omega-3 Fatty Acids, Omega-3 Marine Triglycerides, PUFA, Salmon Oil, Triglycérides Marins, Tuna Fish Oil, Tuna Oil, W-3 Fatty Acids.)
203. Flavonoids
a. Chrysin (Scientific name: 5,7-dihydroxy-2-phenyl-4H-chromen-4-one. Also known as: 5,7-Chrysin, 5,7-Dihydroxyavone, Chrysine, Flavone X, Flavonoid, Flavonoïde, Galangin Flavanone, Galangine Flavanone.)
b. Diosmin (Scientific name: Diosmin. Also known as: 3', 5, 7-trihydroxy-4'-methoxyavone-7-rhamnoglucoside, Bioavonoid, Bioavonoid Complex, Bioavonoid Concentrate, Bioavonoid Extract, Bioavonoïde, Bioavonoïde d'Agrume, Bioavonoïdes d'Agrumes, Citrus Bioavones, Citrus Bioavonoid, Citrus Bioavonoids, Citrus Bioavonoid Extract, Citrus Flavones, Citrus Flavonoids, Complexe de Bioavonoïde, Concentré de Bioavonoïde, Diosmetin 7-O-rutinoside, Diosmina, Diosmine, Extrait de Bioavonoïde, Extrait de Bioavonoïde d'Agrume, Flavonoid, Flavonoïde, Micronised Puried Flavonoid Fraction.)
c. Quercetin (Scientific name: Quercetin. Also known as: 3,3',4′5,7-Pentahydroxyavone, Bioavonoid, Bioavonoid Complex, Bioavonoid Concentrate, Bioavonoid Extract, Bioavonoïde, Bioavonoïde de Citron, Bioavonoïdes de Citron, Citrus Bioavones, Citrus Bioavonoid, Citrus Bioavonoids, Citrus Flavones, Citrus Flavonoids, Complexe de Bioavonoïde, Concentré de Bioavonoïde, Extrait de Bioavonoïde, Extrait de Bioavonoïdes de Citron, Flavones de Citron, Flavonoid, Flavonoïde, Meletin, Mélétine, Quercetin Dihydrate, Quercetina, Quercétine, Sophretin, Sophrétine.)
d. Hesperidin (Scientific name: Hesperidin. Also known as: Bioavonoid, Bioavonoid Complex, Bioavonoid Concentrate, Bioavonoid Extract, Bioavonoïde, Bioavonoïde d'Agrume, Bioavonoïdes d'Agrumes, Citrus Bioavones, Citrus Bioavonoid, Citrus Bioavonoids, Citrus Bioavonoid Extract, Citrus Flavones, Citrus Flavonoids, Complexe de Bioavonoïdes, Concentré de Bioavonoïdes, Extrait de Bioavonoïdes, Extrait de Bioavonoïdes d'Agrumes, Flavonoid, Flavonoïde, Hesperidin Methyl Chalcone, Hesperidina, Hespéridine, Trimethylhesperidin-Chalcon.)
e. Rutin (Scientific name: Rutin, Rutoside. Also known as: 3, 3', 4', 5, 7-pentahydroxyavone-3-rhamnoglucoside, Bioavonoid, Bioavonoid Complex, Bioavonoid Concentrate, Bioavonoid Extract, Bioavonoїde, Bioavonoїdes d'Agrumes, Citrus Bioavonoid, Citrus Bioavones, Citrus Bioavonoid Extract, Citrus Bioavonoids, Citrus Flavones, Citrus Flavonoids, Complexe de Bioavonoїdes, Concentré de Bioavonoїde, Eldrin, Extrait de Bioavonoїde, Flavonoid, Flavonoїde, Flavonoїdes d'Agrumes, Quercetin-3-Rhamnoglucoside, Quercetin-3-Rutinoside, Quercétine-3-Rutinoside, Rutina, Rutine, Rutinum, Rutosid, Rutosidum, Sclerutin, Sophorin.)
204. Flaxseed (Scientific name: Linum usitatissimum, synonyms Linum crepitans, Linum humile. Also known as: Alasi, Aliviraaii, Brown Flaxseed, Brown-Seeded Flax, Common Flax, Echter Lein, Flachs, Flachssamen, Flax, Flax Hull, Flax Lignans, Flax Meal, Flax Seed, Gemeiner Flachs, Golden Flax, Graine de Lin, Kattan, Keten, Leinsamen, Lignanes de Lin, Lignans, Lin, Lin Commun, Lin Oléagineux, Lin Textile, Linaza, Lini Semen, Linho, Lino, Lino Comune, Lino Mazzese, Lino Usuale, Linseed, Linseed Flax, Lint Bells, Linum, Malsag, Phytoestrogen, Phyto-Oestrogène, Saatlein, Ta Ma,Tisii, Winterlien.)
205. Flor Essence Formula (Also known as: Flower Essence.)
206.Flower Remedies
207. Folk Medicine
208. Frangula purshiana
209. Free and Easy Wanderer
210. Fringe Medicine
211. Fructans
212. Fructooligosaccharides (FOS) (Also known as: Chicory Inulin Hydrolysate, Complexe d'Oligosaccharide, FOS, Fructo Oligo Saccharides, Fructo-Oligosacáridos, Fructooligosaccharides, Fructo-Oligosaccharides à Courte Chaîne, Inulin Hydrolysate, Oligofructan, Oligofructose, Oligosaccharide Complex, Oligosaccharides, Prebiotic, Prébiotique, SC-FOS, Short Chain Fructo-Oligosaccharides.)
213. Fu Zheng (Also known as: Fuzheng.)
214. Galactans
215. Galacto-Oligosaccharides (GOS) (Also known as: Galactooligosaccharides.)
216. Gamma Oryzanol (Scientific name: Gamma Oryzanol. Also known as: Gama Orizanol, Gamma-Oryzanol, Gamma-OZ, Oryzanol.)
217. Gamma-Linolenic Acid (GLA) (Scientific name: Gamma Linolenic Acid. Also known as: Acide Gammalinolénique, Acide Gamma-Linolénique, Ácido Gama Linolénico, AGL, Gammalinolenic Acid, Gamma-Linolenic Acid, Gamolenic Acid, GLA, (Z,Z,Z)-Octadeca-6,9,12-Trienoic Acid.)
218. Garcinia (Scientific name: Garcinia gummi-gutta, synonyms Cambogia binucao, Cambogia gemmi-guta, Cambogia solitaria, Garcinia affinis, Garcinia cambogia, Garcinia sulcata, Mangostana cambogia. Also known as: Brindal Berry, Brindle Berry, Gorikapuli, Kankusta, Kudam Puli, Malabar Tamarind, Tamarinier de Malabar, Vrikshamla.)
219. Garlic (Scientific name: Allium sativum. Also known as: Aged Garlic Extract, Ail, Ail Blanc, Ail Cultive, Ail Rocambole, Ajo, Alho, Allii Sativi Bulbus, Allium, Angio D'India, Camphor Of The Poor, Clove Garlic, Common Garlic, Da Suan, Echte Rokkenbolle, Echter Knoblauch, Garlic Oil, Knoblauch, Lahsun, Lasun, Lasuna, Maneul, Nectar Of The Gods, Ninniku, Ophio Garlic, Poor Man's Treacle, Rason, Rocambole, Rockenbolle, Rust Treacle, Schlangenknoblauch, Serpent Garlic, Spanish Garlic, Stinking Rose, Suan, Thoum, Vitlok.)
220. Genistein Combined Polysaccharide (Also known as: Basidiomycetes Polysaccharide, Fermented Genistein, Fermented Isoflavone, GCP, Genistein Polysaccharide, Génistéine du Polysaccharide Combiné, Génistéine Fermentée, Isoflavone Combined Polysaccharide, Isoflavone Fermentée, Polisacáridos Combinados de Genisteína, Polysaccharide de Génistéine, Polysaccharide des Basidiomycètes, Polysaccharide d'Isoflavone de Soja, Soy Isoflavone Polysaccharide.)
221. Germander
a. Germander (Scientific name: Teucrium chamaedrys. Also known as: Camedrio, Chasse-Fièvre, Chêneau, Chenette, Germandrée, Germandrée Officinale, Germandrée Petit Chêne, Petit Chêne, Wall Germander, Wild Germander.)
b. Water Germander (Scientific name: Teucrium scordium. Also known as: Camedrio Acuático, Chamarras, Escordio, Garlic Germander, Germandrée Aquatique, Germandrée d'Eau, Germandrée des Marais, Germandrée Scordium, Germandrina de Agua.)
222. Gerson Therapy/Diet
223. Gestalt Therapy
224. Ginger (Scientific name: Zingiber officinale, synonym Amomum zingiber. Also known as: African Ginger, Ardraka, Black Ginger, Cochin Ginger, Gan Jiang, Gingembre, Gingembre Africain, Gingembre Cochin, Gingembre Indien, Gingembre Jamaïquain, Gingembre Noir, Ginger Essential Oil, Ginger Root, Huile Essentielle de Gingembre, Imber, Indian Ginger, Jamaica Ginger, Jengibre, Jiang, Kankyo, Kanshokyo, Nagara, Race Ginger, Racine de Gingembre, Rhizoma Zingiberi, Rhizoma Zingiberis, Rhizoma Zingiberis Recens, Shen Jiang, Sheng Jiang, Shoga, Shokyo, Shunthi, Srungavera, Sunth, Sunthi, Vishvabheshaja, Zingiberis Rhizoma, Zingiberis Siccatum Rhizoma, Zinzeberis, Zinziber Officinale, Zinziber Officinalis.)
225. Ginkgo (Scientific name: Ginkgo biloba. Also known as: Abricot Argenté Japonais, Adiantifolia, Arbe aux Écus, Arbe aux Quarante Écus, Arbe du Ciel, Arbre Fossile, Bai Guo Ye, Baiguo, Extrait de Feuille de Ginkgo, Extrait de Ginkgo, Fossil Tree, Ginkgo Biloba Leaf, Ginkgo Folium, Graine de Ginkgo, Herba Ginkgo Biloba, Japanese Silver Apricot, Kew Tree, Maidenhair Tree, Noyer du Japon, Pei Go Su Ye, Salisburia Adiantifolia, Yen Xing, Yinhsing)
226. Ginseng
a. Panax ginseng (Scientific name: Panax ginseng, synonym Panax schinseng. Also known as: sian Ginseng, Asiatic Ginseng, Chinese Ginseng, Chinese Red Ginseng, Ginseng, Ginseng Asiatique, Ginseng Blanc, Ginseng Blanc de Corée, Ginseng Chinois, Ginseng Coréen, Ginseng Coréen Rouge, Ginseng de Corée, Ginseng Japonais, Ginseng Oriental, Ginseng Panax, Ginseng Radix Alba, Ginseng Root, Ginseng Rouge, Ginseng Sino-coréen, Ginseng Tibétain, Guigai, Hong Shen, Insam, Japanese Ginseng, Jen-Shen, Jinsao, Jintsam, Korean Ginseng, Korean Ginseng Root, Korean Panax, Korean Panax Ginseng, Korean Red Ginseng, Korean White Ginseng, Manchurian Ginseng, Mandragore de Chine, Ninjin, Ninzin, Oriental Ginseng, Panax Coréen, Panax Ginseng Blanc, Racine de Vie, Radix Ginseng Rubra, Red Chinese Ginseng, Red Ginseng, Red Kirin Ginseng, Red Korean Ginseng, Red Panax Ginseng, Renshen, Renxian, Sheng Shai Shen, Tibetan Ginseng, White Ginseng, White Panax Ginseng.)
b. American Ginseng (Scientific Name: Panax quinquefolius. Also known as: Anchi Ginseng, Baie Rouge, Canadian Ginseng, Ginseng, Ginseng à Cinq Folioles, Ginseng Américain, Ginseng Americano, Ginseng d'Amérique, Ginseng D'Amérique du Nord, Ginseng Canadien, Ginseng de l'Ontario, Ginseng du Wisconsin, Ginseng Occidental, Ginseng Root, North American Ginseng, Occidental Ginseng, Ontario Ginseng, Panax Quinquefolia, Panax Quinquefolium, Racine de Ginseng, Red Berry, Ren Shen, Sang, Shang, Shi Yang Seng, Wisconsin Ginseng, Xi Yang Shen.)
227. Glucosamine (Scientific name: 3-Amino-6-(Hydroxymethyl)Oxane-2,4,5-Triol Sulfate. Also known as: (3R,4R,5S,6R)-3-Amino-6-(Hydroxymethyl)Oxane-2,4,5-Triol Hydrochloride, 2-Acetamido-2-deoxyglucose, 2-Amino-2-Deoxy-Beta-D-Glucopyranose Hydrochloride, 2-Amino-2-Deoxy-D-Glucosehydrochloride, 2-Amino-2-Deoxy-Beta-D-Glucopyranose, 2-Amino-2-Deoxy-D-Glucose Sulfate, Acetylglucosamine, Acétylglucosamine, GlcNAc, Chitosamine, Chitosamine Hydrochloride, Chlorhidrato de Glucosamina, Chlorhydrate de Glucosamine, D-Glucosamine HCl, D-Glucosamine Hydrochloride, D-Glucosamine Sulfate, D-Glucosamine Sulphate, G6S, Glucosamine HCl, Glucosamine KCl, Glucosamine NAcetyl, Glucosamine Potassium Sulfate, Glucosamine Sulfate, Glucosamine Sulfate 2KCl, Glucosamine Sulfate-Potassium Chloride, Glucosamine Sulphate, Glucosamine Sulphate KCl, Glucosamine-6-Phosphate, GS, Mono-Sulfated Saccharide, NAcetil Glucosamina, N-Acétyl Glucosamine, N-Acétyl-Glucosamine, N-Acétylglucosamine, N-Acetyl D-Glucosamine, N-Acétyl D-Glucosamine, NAG, N-A-G, pGlcNAc, Poly-N-Acetyl Glucosamine, Poly-NAG, Poly-(1- > 3)-N-Acetyl-2-Amino-2-Deoxy-3-OBeta-D-Glucopyranurosyl-4-(or 6-) Sul, p-GlcNAc, Saccharide Mono-Sulfaté, Saccharide Sulfaté, Sulfate de Glucosamine, Sulfate de Glucosamine 2KCl, SG, Sulfated Monosaccharide, Sulfated Saccharide, Sulfato de Glucosamina.)
228. Glutamine (Scientific name: L-( +)-2-Aminoglutaramic acid. Also known as: Acide Glutamique, Acide Glutamique HCl, Acide L-( +)-2-Aminoglutaramique, Acide L-Glutamique, Acide L-Glutamique HCl, Alanyl-L-Glutamine Dipeptide, Éthyle Ester de Glutamine, Éthyle Ester de Glutamine HCl, GLN, Glutamate, Glutamic Acid, Glutamic Acid HCl, Glutamic Acid Hydrochloride, Glutamina, Glutaminate, Glutamine Ethyl Ester, Glutamine Ethyl Ester HCl, Glutamine Methyl Ester, Glutamine Peptides, Levoglutamide, Levoglutamine, L-Alanyl-L-Glutamine, L-Glutamic Acid, L-Glutamic Acid HCl, L-Glutamic Acid Hydrochloride, L-Glutamic Acid 5-Amide, L-Glutamine, N-Acetyl-L-Glutamine, Peptides de Glutamine, Q, (S)-2,5-Diamino-5-oxopentanoic Acid.)
229. Gluten-Free Diet (Also known as: Wheat-Free Diet.)
230. Glyconutrients (Also known as: Ambrotose, Gluconutrientes, Glyconutriments, Manapol.)
231. Grahamism
232. Grape Seed (Scientific name: Vitis vinifera, Vitis labrusca. Also known as: Activin, Black Grape Raisins, Calzin, Concord Grape, Draksha, Enocianina, European Wine Grape, Extrait de Feuille de Raisin, Extrait de Feuille de Vigne Rouge, Extrait de Peau de Raisin, Extrait de Pepins de Raisin, Feuille de raisin, Feuille de Vigne Rouge, Feuille de Vigne Rouge AS 195, Flame Grape, Flame Raisins, Flame Seedless, Folia Vitis Viniferae, Fox Grape, Grape Fruit, Grape Fruit Skin, Grape Juice, Grape Leaf, Grape Leaf Extract, Grape Polyphenols, Grape Pomace, Grape Seed, Grape Seed Extract, Grape Seed Oil, Grape Skin, Grape Skin Extract, Grapes, Grapeseed, Huile de Pépins de Raisin, Kali Draksha, Leucoanthocyanin, Muscat, Muskat, Oligomères Procyanidoliques, Oligomeric Proanthocyanidins, Oligomeric Procyanidins, OPC, OPCs, PCO, PCOs, Peau de Raisin, Pépin de Raisin, Petite Sirah, Proanthocyanidines Oligomériques, Proanthodyn, Proanthodyne, Procyanidines Oligomériques, Procyanidolic Oligomers, Purple Grape, Raisin, Raisin Blanc, Raisin de Table, Raisin de Vigne, Raisins, Raisins Noirs, Red Globe, Red Grape, Red Malaga, Red Vine, Red Vine Leaf AS 195, Red Vine Leaf Extract, Skunk Grape, Sultanas, Table Grapes, Thompson Seedless, Uva, Vin Rouge, White Grape, Wine Grape, Wine Grapes.)
233. Grapefruit (Scientific name: Citrus paradisi. Also known as: Bioflavonoid Complex, Bioflavonoid Concentrate, Bioflavonoid Extract, Bioflavonoids, Bioflavonoïdes, Bioflavonoïdes d'Agrumes, Citrus Bioflavones, Citrus Bioflavonoid, Citrus Bioflavonoid Extract, Citrus Bioflavonoids, Citrus Extract, Citrus Flavones, Citrus Flavonoids, Citrus Grandis Extract, Citrus Seed Extract, Cold-Pressed Grapefruit Oil, Complexe Bioflavonoïde, Complexe Bioflavonoïde de Pamplemousse, Concentré de Bioflavonoïde, CSE, Expressed Grapefruit Oil, Extrait d'Agrume, Extrait de Bioflavonoïde, Extrait de Bioflavonoïdes d'Agrumes, Extrait de Graines de Pamplemousse, Extrait de Pamplemousse, Extrait Normalisé de Pamplemousse, Flavonoïdes d'Agrumes, Grapefruit Bioflavonoid Complex, Grapefruit Extract, Grapefruit Oil, Grapefruit Seed Extract, Grapefruit Seed Glycerate, GSE, Huile de Pamplemousse, Huile de Pamplemousse Pressée à Froid, Pamplemousse, Pamplemousse Rose, Paradisapfel, Pink Grapefruit, Pomelo, Red Mexican Grapefruit, Shaddock Oil, Standardized Extract of Grapefruit, Toronja.)
234. Gravity Inversion (Also known as: Inversion Therapy.)
235. Greater Celandine (Scientific name: Chelidonium majus. Also known as: Bai Qu Cai, Celandine, Celidonia Mayor, Chelidonii Herba, Grande Chélidoine, Grande Éclaire, Herbe à Verrues, Herbe aux Verrues, Parties Aériennes de la Grande Chélidoine, Racine de Chélidoine, Racine de Grande Chélidoine, Rhizome de Chélidoine, Rhizome de la Grande Chélidoine, Schollkraut, Swallow Wort, Tetterwort, Verruguera.)
236. Green Tea (Scientific name: Camellia sinensis, synonyms Camellia thea, Camellia theifera, Thea bohea, Thea sinensis, Thea viridis. Also known as: Benifuuki, Constituant Polyphénolique de Thé Vert, CPTV, EGCG, Epigallo Catechin Gallate, Épigallo-Catéchine Gallate, Epigallocatechin Gallate, Extrait de Thé Vert, Extrait de Camellia Sinensis, Extrait de Thé, Extrait de Thea Sinensis, Green Sencha Tea, Green Tea Extract, Green Tea Polyphenolic Fraction, GTP, GTPF, Japanese Sencha Green Tea, Japanese Tea, Kunecatechins, Poly E, Polyphenon E, PTV, Té Verde, Tea Extract, Tea Green, Tea, Thé, Thé de Camillia, Thé Japonais, Thé Vert de Yame, Thé Vert, Thé Vert Sensha, Yabukita, Yame Green Tea, Yame Tea.)
237. Group Therapy (Also known as: Group Support, Support Groups, Social Support.)
238. Gso-Ba Rig-Pa (Also known as: Gsoba Rig-Pa, Gso-Ba Rigpa.)
239. Guarana (Scientific name: Paullinia cupana, synonym Paullinia sorbilis. Also known as: Brazilian Cocoa, Cacao Brésilien, Guarana Seed Extract, Guaranine, Zoom.)
240. Guggul (Scientific name: Commiphora wightii, synonyms Commiphora mukul, Balsamodendrum wightii, Balsamodendrum mukul. Also known as: Devadhupa, Gomme Guggul, Gomme-Résine de Guggul, Guggal, Guggul Gum, Guggul Gum Resin, Guggul Lipids, Guggulipid, Guggulipide, Guggulsterone, Guggulstérone, Guggulsterones, Guggulstérones, Guggulu, Guggulu Suddha, Guglipid, Gugulipid, Gum Guggal, Gum Guggulu, Indian Bdellium, Indian Bdellium-Tree, Indian Guggulipids, Koushika, Mukul Myrrh Tree, Palankasha, Yogaraj Guggul Gum Resin.)
241. Guided Imagery (Also known as: Guided Health Imagery, Guided Visualization, Imagerie Guidée, Imagery, Imaginación Guiada, Visualisation, Visualisation Guidée, Visualization, Visualization Therapy, VT.)
242. Guiling Pa'an Wan
243. Gurah
244. Hair Analysis
245. Hands-On Healing
246. Hawthorn (Scientific name: Crataegus monogyna, Crataegus laevigata, synonyms Crataegus oxyacantha, Mespilus laevigata, Crataegus cuneata, synonym Crataegus kulingensis, Crataegus pinnati da, Crataegus rhipidophylla, Crataegus pentagyna. Also known as: Aubepine, Aubépine, Aubépine Blanche, Aubépine Épineuse, Bianco Spino, Bois de Mai, Cenellier, Chinese Hawthorn, Crataegi Flos, Crataegi Folium, Crataegi Folium Cum Flore, Crataegi Fructus, Crataegus, English Hawthorn, Epine Blanche, Epine de Mai, Espino Blanco, Fructus Crataegi, Haagdorn, Hagedorn, Harthorne, Haw, Hawthorne, Hedgethorn, LI 132, LI132, May, Maybush, Maythorn, Mehlbeebaum, Meidorn, Nan Shanzha, Noble Épine, Shen Zha, Oneseed Hawthorn, Poire d'Oiseaux, Sable Épine, Shanzha, Weissdorn, Whitehorn, WS 1442, WS1442.)
247. Heliotherapy
248. Heliotrope
249. Hellerwork (Also known as: Deep Tissue Bodywork and Movement Education, Structural Integration.)
250. Helminth Therapy (Also known as: Trichuris suis ova, Trichuris trichiura ova.)
251. Herbal Tea (Also known as: Tisane.)
252. High Fiber Diet
253. Hirudotherapy (Also known as: Hirudin, Hirudoterapia, Hirudothérapie, Leech, Leech Anticoagulants, Leech Saliva, Leech Therapy, Leeches Medicinalis, Mechanical Leech Therapy, Medicinal Leech Therapy, Medicinal Leeches, Sangsue Anticoagulante, Sangsue Médicinale, Sangsue Officinale, Thérapie par les Sangsues.)
254. Holistic Health (Also known as: Holism.)
255. Holographic/Resonance Repatterning
256. Home Remedies
257. Homeopathy (Also known as: Homeopathic, Homeopathic Medicine, Homeopathic Nosodes, Homeopathic Remedies, Homeopathie, Homéopathique, Homeopatía, Homeoprophylaxis, Maladie Semblable, Médecine Homéopathique, Nosodes, Remède Homéopathique, Similar Disease.)
258. Homoharringtonine
259. Homotoxicology
260. Hoodia (Scientific name: Hoodia gordonii. Also known as: Cactus, Cactus Hoodia, Cactus du Kalahari, Extrait de Hoodia, Hoodia Gordonii Cactus, Hoodia P57, Kalahari Cactus, Kalahari Diet, P57, Xhoba.)
261. Hops (Scientific name: Humulus lupulus. Also known as: Asperge Sauvage, Common Hops, Couleuvrée, Couleuvrée Septentrionale, European Hops, Hop, Hop Strobile, Hopfenzapfen, Houblon, Lupuli Strobulus, Lupulin, Lúpulo, Pi Jiu Hua, Salsepareille Indigène, Vigne du Nord.)
262. Horse Chestnut (Scientific name: Aesculus hippocastanum. Also known as: Aescin, Buckeye, Castaño de Indias, Châtaignier de Mer, Châtaignier des Chevaux, Chestnut, Conker Tree, Escine, Faux-Châtaignier, Hippocastani Cortex, Hippocastani Flos, Hippocastani Folium, Hippocastani Semen, Hippocastanum Vulgare Gaertn, Marron Europeen, Marronnier, Marronnier Blanc, Marronnier Commun, Marronnier d'Inde, Marronnier des Chevaux, Pu, Spanish Chestnut, Venastat, Venostat, Venostasin Retard, White Chestnut.)
263. Horticultural Therapy
264. Hoxsey
265. Humor Therapy
266. Huna
267. Huperzine (Scientific name: Huperzine A. Also known as: HupA, Huperzina A, Huperzine, Huperzine-A, Selagine, Sélagine.)
268. Hydrazine Sulfate (Scientific name: Hydrazine Sulfate. Also known as: Segidrin, Sehydrin, Sulfate d'Hydrazine, Sulfato de Hidracina.)
269. Hydrogen Peroxide
270. Hydrotherapy (Also known as: Hot and Cold Water Treatment, Watsu.)
271. Hypnotherapy (Also known as: Altered States Of Consciousness, Antenatal Self-Hypnosis, Autogenic Training, Auto-Hypnosis, Cognitive Hypnotherapy, Hypnoanalgesia, Hypnobirthing, Hypnosis, Mesmerism, Post-Hypnotic Suggestion, Self-Hypnosis.)
272. Immune Boosters
273. Immunoaugmentative Therapy (Also known as: IAT, Immuno-Augmentative Therapy, Immunoaugmentation.)
274. Initiatory Medicine
275. Inositol (Scientific name: Hexahydroxycyclohexane, synonyms 1,2,3,4,5,6-Cyclohexanehexol, cis-1,2,3,5-trans-4,6-Cyclohexanehexol; D-chiro-inositol, synonym ( +)-chiroinositol, 1,2,5/3,4,6-inositol, (1S)-inositol, (1S)-1,2,4/3,5,6-inositol. Also known as: Antialopecia Factor, Cyclohexitol, Dambrose, D-Myo-Inositol, Facteur Anti-Alopécique, Inose, Inosite, Inositol Monophosphate, Lipositol, Meso-Inositol, Méso-Inositol, Monophosphate d'Inositol, Mouse Antialopecia Factor, Myo-Inositol, Vitamin B8, Vitamine B8.)
276. Integrative/Integrated Medicine (Also known as: Integrative/Integrated Therapy, Integrative/Integrated Approach, Integrative/Integrated Health.)
277. Interactive Metronome
278. Iodine (Scientific name: I, Atomic number 53. Also known as: Cadexomer Iodine, Diatomic Iodine, I2, Iode, Iode de Cadexomer, Iode Diatomique, Iode Moléculaire, Iode Mono-Atomique, Iode de Povidone, Iode de Sodium, Iodide, Iodized Salt, Iodure, Iodure de Potassium, Iodure de Potassium en Solution Saturée, Iodure de Sodium, KI, Lugol's Solution, Molecular Iodine, Monoatomic Iodine, Numéro Atomique 53, Periodate de Sodium, Potassium Iodide, Potassium Triiodide, Povidone Iodine, Saturated Solution Potassium Iodide, Sel Iodé, Sodium Iodide, Sodium Iodine, Sodium Periodate, Solution de Lugol, SSKI, Yodo.)
279. Ipriflavone (Scientific name: 7-isopropoxyisoflavone. Also known as: 7-Isopropoxy-Isoflavone, 7-Isopropoxy Isoflavone, FL-113, Ipriflavona, TC-80.)
280. Iridology (Also known as: Bilan Iridologique, Diagnostic par l'Iris, Irido-Diagnostique, Iridiologie, Iridología, Iridologist, Iridologue, Iridology Analysis, Iris, Iris Diagnosis.)
281. Iron (Scientific name: Fe, Atomic number 26. Also known as: Carbonate de Fer Anhydre, Citrate de Fer, Elemental Iron, Fer, Fer Élémentaire, Ferric Iron, Ferric Hydroxide Polymaltose, Ferric Orthophosphate, Ferric Oxide Saccharide, Ferric Sodium Citrate, Ferrous Carbonate Anhydrous, Ferrous Citrate, Ferrous Fumarate, Ferrous Gluconate, Ferrous Iron, Ferrous Pyrophosphate, Ferrous Succinate, Ferrous Sulfate, Ferrum Phosphoricum, Fumarate de Fer, Gluconate de Fer, Glycérophosphate de Fer, Heme Iron Polypeptide, Hierro, Iron Glycerophosphate, Iron Polysaccharide, Orthophosphate de Fer, Orthophosphate Ferrique, Numéro Atomique 26, Polypeptide de Fer de Heme, Pyrophosphate de Fer, Sulfate de Fer.)
282. Islamic Medicine
283. Isoflavones
284. Jaffe Mellor Techniques
285. Jamu
286. Jasmine (Scientific name: Jasminum grandiflorum, synonym Jasminum officinale. Also known as: Catalonina Jasmine, Common Jasmine, Italian Jasmine, Jasmin, Jasmin Blanc, Jasmin Commun, Jasmind'Espagne, Jasmin à Grandes Fleurs, Jasmin Officinal, Jasmin Royal, Jati, Jazmín, Jazmín Silvestre, Poet's Jessamine, Royal Jasmine, Spanish Jasmine.)
287. Jin Bu Huan
288. Jin Li Da Liquor
289. Jin Shin
290. Juice Fasting (Also known as: Juice Cleanse, Juice Detox, Juice Therapy, Juicing.)
291. Kampo Medicine (Also known as: Japanese Herbal Medicine, Japanese Medicine, JM, Médecine Japonaise, Médecine Kampo, Médecine Orientale, Médecine Traditionnelle Asiatique, Medicina Kanpo, Médecine Kampo, Oriental Medicine, TAM, TJM, Traditional Asian Medicine.)
292. Kava (Scientific name: Piper methysticum. Also known as: Ava Pepper, Ava Root, Awa, Gea, Gi, Intoxicating Long Pepper, Intoxicating Pepper, Kao, Kavain, Kavapipar, Kawa, Kawa Kawa, Kawa Pepper, Kawapfeffer, Kew, Lawena, Long Pepper, Malohu, Maluk, Maori Kava, Meruk, Milik, Poivre des Cannibales, Poivre des Papous, Rauschpfeffer, Rhizome Di Kava-Kava, Sakau, Tonga, Waka, Wurzelstock, Yagona, Yangona, Yaqona, Yaquon, Yongona.)
293. Ketogenic Diet (Also known as: Classical Ketogenic Diet, Classic Long-Chain Triglyceride Ketogenic Diet, Keto Diet, LCD, LCHPD, Low-Carb Diet, Low-Carbohydrate Diet, Low-Carbohydrate High-Protein Diet, Low Carb Diet, Low Carbohydrate Diet, Low Glycemic Index Treatment, Medium Chain Triglyceride Diet, Modied Atkin's Diet, Very Low Carbohydrate Diet, Very-Low-Carbohydrate Ketogenic Diet.)
294. Kinesiology Services
295. Kirlian Photography (Also known as: Aura Photography, Coronal Discharge Photography, Fingertip Aura, Gas Discharge Visualization (GDV), Kirlian Diagnostics, Kirlian Electrophotography, Kirlian-Graphic, Spark Electrography, Tesla Coil Kirlian Photography.)
296. Kneipp Cure
297. Kombucha (Also known as: Algue de Thé, Champagne of Life, Champignon de la Charité, Champignon des Héros, Champignon de Longue Vie, Champignon Miracle, Combucha Tea, Dr. Sklenar's Kombucha Mushroom Infusion, Fungus Japonicus, Kargasok Tea, Kombucha Tea, Kombucha Mushroom Tea, Kombucha Thé, Kwassan, Laminaire de Thé, Manchurian Fungus, Manchurian Mushroom Tea, Mushroom Infusion, Petite Mère Japonaise, Spumonto, T'Chai from the Sea, Té de Kombucha, Thé de Combucha, Thé de Kombucha, Tschambucco.)
298. Krebiozen (Also known as: Carcalon, Drug X, Substance X.)
299. La'au Lapa'au
300. Laetrile (Also known as: Amygdalin.)
301. Lam Kam San Heklin
302. L-Arginine (Scientific name: 2-Amino-5-guanidinopentanoic acid. Also known as: 2-Amino-5-(diaminomethylidene amino) pentanoic acid, (2S)-2-Amino-5-{[amino (imino) methyl]amino}pentanoic Acid, (S)-2-Amino-5- Guanidinopentanoic Acid, Acide 2-Amino-5-Guanidinopentanoïque, Arg, Arginine, Arginine Aspartate, Arginine Ethyl Ester, Arginine Ethyl Ester Dihydrochloride, Arginine Ethyl Ester HCl, Arginine HCl, Arginine Hydrochloride, Di-Arginine Malate, Di-Arginine Orotate, Di-L-Arginine-L-Malate, Dl-Arginine, L-Arginina, L-Arginine Ethyl Ester Dichloride, L-Arginine HCl, L-Arginine Hexanoate, L-Arginine Hydrochloride, L-Arginine Ketoisocaproic Acid, LArginine L-Pyroglutamate, L-Arginine Pyroglutamate, L-Arginine Taurinate, Malate de Di-Arginine, Orotate de Di-Arginine, RGene 10.)
303. Laser Therapy
304. Laughter Therapy
305. Lavender (Scientific name: Lavandula angustifolia, synonyms Lavandula officinalis, Lavandula vera, Lavandula spica, Lavandula dentata, Lavandula latifolia, Lavandula pubescens, Lavandula stoechas. Also known as: Alhucema, Common Lavender, English Lavender, French Lavender, Garden Lavender, Huile Essentielle de Lavande, Lavanda, Lavande, Lavande à Feuilles Étroites, Lavande Anglaise, Lavande Commune, Lavande des Alpes, Lavande du Jardin, Lavande Espagnole, Lavande Fine, Lavande Française, Lavande Officinale, Lavande Vraie, Lavandula, Lavender Essential Oil, Ostokhoddous, Spanish Lavender, Spike Lavender, True Lavender.)
306. Leafflower (Scientific name: Phyllanthus)
307. Lecithin (Also known as: Egg Lecithin, Lécithine, Lécithine d'œuf, Lécithine de Graine de Soya, Lécithine de Soya, Lecitina, Ovolecithin, Ovolécithine, Phospholipide de Soja, Phospholipide de Soya, Phospholipides de Soya, Soy Lecithin, Soy Phospholipid, Soy Phospholipids, Soya Bean Lecithin, Soya Lecithin, Soybean Lecithin, Vegilecithin, Vitellin, Vitelline.)
308. Lemon Balm (Scientific name: Melissa officinalis. Also known as: Balm, Balm Mint, Bálsamo de Limón, Common Balm, Cure-All, Dropsy Plant, Honey Plant, Melisa, Melissa, Melissae Folium, Mélisse, Mélisse Citronnelle, Mélisse Officinale, Melissenblatt, Monarde, Sweet Balm, Sweet Mary, Toronjil.)
309. Lentinan (Also known as: Beta-1,3-glucan Lentinan, Beta-glucan Lentinan, Lentinane, Lentinus Edodes Polysaccharide, Polysaccharide dérivé de Lentinus Edodes, Xiangguduotang, Xiangguduotangzhusheye.)
310. Licorice (Scientific name: Glycyrrhiza echinate, Glycyrrhiza glabra, synonym Glycyrrhiza glandulifera, Glycyrrhiza glabra var. glandulifera; Glycyrrhiza uralensis. Also known as: Acide Glycyrrhizique, Acide Glycyrrhizinique, Alcacuz, Alcazuz, Bois Doux, Bois Sucré, Can Cao, Chinese Licorice, Deglycyrrhized Licorice, East European Licorice, Gan Cao, Gan Zao, Glabra, Glycyrrhiza, Glycyrrhiza Radix, Glycyrrhizae, Glycyrrhizic Acid, Glycyrrhizinic Acid, Isoavone, Jethi-Madh, Kanzo, Lakritze, Liquiritiae Radix, Liquirizia, Liquorice, Mulathi, Mulethi, Orozuz, Phytoestrogen, Phyto-Œstrogène, Racine de Réglisse, Racine Douce, Radix Glycyrrhizae, Régalissse, Regaliz, Reglisse, Réglisse, Réglisse Déglycyrrhisée, Réglisse Espagnole, Réglisse Russe, Regaliz, Regliz, Russian Licorice, Spanish Licorice, Subholz, Sussholz, Sweet Root, Turkish Licorice, Ural Licorice, Yashtimadhu, Yashti-Madhu, YashtiMadhuka, Zhi Gan Cao.)
311. Lifestyle Changes
312. Light Therapy
a. Light Therapy (Also known as: Acu-Light Therapy, Balneophototherapy, Bath PUVA, Bright Light Therapy, Dead Sea Climatotherapy (DSC), Esogetic Colorpuncture, Fiber-Optic Phototherapy, Irradiation, LEDs, Light Irradiation, Light Therapy, Light Treatment, Light-Emitting Diode Phototherapy, Nontargeted Light Therapy, Ocular Light Therapy (OLT), PDT Photo Therapy, Photobiology, Photochemotherapy, Photodynamic Therapy, Photomedicine, Photophoresis, Photosensitizers, Phototherapy, Reprocessing Light Therapy, Targeted Light Therapy, Ultraviolet (UV) Therapy.)
b. Anodyne Therapy (Also known as: Light Therapy, MIRE, Monochromatic Infrared Photo Energy, Phototherapy.)
313. Lime (Scientific name: Citrus aurantifolia, synonyms Citrus medica var. acida, Citrus acida, Citrus lima, Citrus limetta var. aromatica, Limonia aurantifolia. Also known as: Dam's Apple, Bara Nimbu, Bijapura, Citron Vert, Citronnier Vert, Huile de Lime, Italian Limetta, Key Lime, Lima, Lime Oil, Limette, Limettier, Turanj.)
314. L-Isoleucine
315. Liuwei Dihuang Pills
316. Live Cell Therapy
317. Livingston Wheeler Therapy
318. L-Leucine
319. Low Carbohydrate Diet
320. Low Fat Diet
321. Low Glycemic Index Diet (Also known as: Low GI Diet.)
322. Low Protein Diet
323. L-Valine
324. Lymph Therapy
325. Lysine (Scientific name: L-2,6-diaminohexanoic acid. Also known as: Hydrochlorure de L-Lysine, Lisina, L-Lysine, L-Lysine HCl, L-Lysine Hydrochloride, L-Lysine Monohydrochloride, Lys, Lysine HCl, Lysine Hydrochloride, Lysine Monohydrochloride, Monochlohydrate de L-Lysine, Monochlohydrate de Lysine.)
326. Macrobiotic Diet (Also known as: Ma-Pi 2 Diet, Macrobiotics, Macrobiotism, Zen Macrobiotics.)
327. Maggot Therapy (Also known as: Biodebridement, Biosurgery, Biosurgical Debridement, Biosurgical Management, Biosurgical Wound Debridement, Biotherapy, Larva Therapy (LT), Larvae Therapy, Larval Debridement Therapy (LDT), Maggot Debridement Therapy (MDT).)
328. Magnesium (Scientific name: Mg, Atomic number 12. Also known as: Aspartate de Magnésium, Carbonate de Magnésium, Chelated Magnesium, Chlorure de Magnésium, Citrate de Magnésium, Dimagnesium Malate, Epsom Salts, Gluconate de Magnésium, Glycérophosphate de Magnésium, Glycinate de Magnésium, Hydroxyde de Magnésium, Lactate de Magnésium, Lait de Magnésium, Magnesia, Magnesia Carbonica, Magnesia Muriatica, Magnesia Phosphorica, Magnesia Sulfate, Magnesia Sulfurica, Magnesio, Magnésium, Magnesium Ascorbate, Magnesium Aspartate, Magnesium Carbonate, Magnésium Chelaté, Magnesium Chloride, Magnesium Citrate, Magnesium Disuccinate Hydrate, Magnesium Gluconate, Magnesium Glycerophosphate, Magnesium Glycinate, Magnesium Hydroxide, Magnesium Lactate, Magnesium Malate, Magnesium Murakab, Magnesium Orotate, Magnesium Oxide, Magnesium Phosphate, Magnesium Phosphoricum, Magnesium Sulfate, Magnesium Taurate, Magnesium Taurinate, Magnesium Trisilicate, Malate de Magnésium, Milk of Magnesia, Numéro Atomique 12, Orotate de Magnésium, Oxyde de Magnésium, Phosphate de Magnésium, Sels d'Epsom, Sulfate de Magnésium, Trisilicate de Magnésium.)
329. Magnet Therapy (Also known as: Aimant, Aimant Statique, Biomagnetism, Biomagnétisme, Bracelet Magnétique, Collier Magnétique, Electromagnetic Therapy, Magnet, Magnetic, Magnetic Bands, Magnetic Bracelet, Magnetic Mattress, Magnetic Necklace, Magnetic Stimulation, Magnetism, Magnétisme, Magnetoterapia, Magnétothérapie, Magnets, Matelas Magnétique, PEMF, PEMFT, PEMT, Pulsed Electromagnetic Therapy, Pulsed Electromagnetic Field Therapy, Repetitive Magnetic Stimulation, Static Magnet, Static Magnet Therapy, Stimulation Magnétique, Stimulation Magnétique Transcranienne, Terapia con Campos Magnéticos, Thérapie par Champ Électromagnétique, Thérapie par Champ Électromagnétique Pulsé, Thérapie Électromagnétique, Thérapie Magnétique.)
330. Maharishi Amrit Kalish (Also known as: Lalish Method.)
331. Maintenance Therapy
332. Maitake Mushroom (Scientific name: Grifola frondosa. Also known as: Champignon Dansant, Champignon des Fous Dansants, Champignon Maitake, Dancing Mushroom, Grifola, Hen of the Woods, Hongo Maitake, King Of Mushrooms, Maitake, Monkey's Bench, Mushroom, Ram's Head, Roi des Champignons, Sheep's Head, Shelf Fungi.)
333. Manganese (Scientific name: Mn, Atomic number 25. Also known as: Aminoate de Manganèse, Ascorbate de Manganèse, Chlorure de Manganèse, Citrate de Manganèse, Complexe Aspartate de Manganèse, Dioxyde de Manganèse, Gluconate de Manganèse, Glycérophosphate de Manganèse, Manganèse, Manganese Amino Acid Chelate, Manganese Aminoate, Manganese Ascorbate, Manganese Aspartate Complex, Manganese Chloride, Manganese Chloridetetrahydrate, Manganese Citrate, Manganese Dioxide, Manganese Gluconate, Manganese Glycerophosphate, Manganese Sulfate, Manganese Sulfate Monohydrate, Manganese Sulfate Tetrahydrate, Manganeso, Manganum, Monohydrate de Sulfate de Manganèse, Sulfate de Manganès.)
334. Mantras
335. Manual Lymphatic Drainage
336. Marma Therapy
337. Marshmallow (Scientific name: Althaea officinalis, synonym Althaea taurinensis. Also known as: Altea, Alteia, Althaeae Folium, Althaeae Radi, Althea, Althée, Guimauve, Guimauve Officinale, Gulkhairo, Herba Malvae, Mallards, Malvavisco, Marsh Maillo, Mauve Blanche, Mortification Root, Racine de Guimauve, Sweet Weed, Wymote.)
338. Massage (Also known as: Abdominal Massage, Abdominal Meridian Massage, Acupuncture Massage, Aromatherapy Massage, Bindegewebsmassage, Chair Massage, Classical Massage, Connective Tissue Manipulation, Connective Tissue Massage, Deep-Tissue Massage, Deep Transverse Friction Massage, Digital Massage, Effleurage Massage, Esalen Massage, Foot Massage, Foot Reflexion Massage, Hand Massage, Hot Stone Massage, Ice Massage, Infant Massage, Integrative Massage, Lomilomi Massage, Marma Massage Therapy, Myofascial Release, Neuromuscular Massage, Oil Massage, Perineal Massage, Petrissage, Prostate Massage, Qigong Massage, Roll-Stretch Massage, Rhythmical Massage, Shiatsu, Skin Rehabilitation Massage Therapy, Sports Massage, Swedish Massage, Thai Massage, Therapeutic Massage, Tibetan Massage, Trigger Point Massage, Tui Na.)
339. Medical Hydrology
340. Medicinal Fungi
341. Meditation
a. Mantra Meditation (Also known as: Concentrative Meditation, Meditación, Méditation, Méditation Concentrative, Méditation de Pleine Conscience, Méditation Transcendantale, TM, Transcendental Meditation.)
b. Mindfulness (Also known as: Mindfulness Meditation, MBRP, MBSR, Mindfulness-Based Relapse Prevention, Mindfulness-Based Stress Reduction.)
c. Yoga (Also known as: Asana, Ashtanga Yoga, Bhakti Yoga, Bikram Yoga, Creative Yoga, Exercice de Yoga, Hatha Yoga, Hot Yoga, Iyengar Yoga, Jinana Yoga, Kripalu Yoga, Kundalini Yoga, Laughter Yoga, Power Yoga, Pranayama, Kundaliniyoga, Raja Yoga, Relaxing Yoga, RY, Sahaj Yoga, Sivananda Yoga, SKY, Sudarshana Kriya Yoga, Surya Namaskara, Tantra Yoga, Tibetan Yoga, Viniyoga, Vinyasa Yoga, Yoga Bikram, Yoga Chaud, Yoga Exercise, Yoga Iyengar, Yoga Meditation, Yoga de Méditation, Yoga Practice, Yoga de Relaxation, Yoga Nidra, Yoga Tantrique, Yoga Therapy, Yoga Tibétain, Yogasana, Yogic Training.)
d. Kundalini Yoga (Also known as: Circular Yoga, Coiled Yoga, Raja Yoga, Roya Yoga, Yoga of Awareness.)
e. Yogoda (Also known as: Kriya Yoga, Meditation-Based Yoga, Raja Yoga.)
342. Mediterranean Diet (Also known as: Diet Mediterranean, MeDi, Mediterranean Eating Pattern, Mediterranean-Style, Mediterranean-Style Diet.)
343. Medium Chain Triglycerides (MCTs) (Scientific name: Medium Chain Triglycerides. Also known as: 1,2,3-Propanetriol Trioctanoate, AC-1202, Acide Caprique, Acide Caproïque, Acide Caprylique, Acide Laurique, Capric Acid, Caproic Acid, Caprylic Acid, Caprylic Triglycerides, Laurate-Rich MCTs, Lauric Acid, MCT, MCT's, MCTs, Medium-Chain Triacylglycerols, Medium-Chain Triglycerides, TCM, Triacylglycérols à Chaîne Moyenne, Tricaprylin, Triglycérides à Chaîne Moyenne, Triglycérides Capryliques, Triglicéridos de Cadena Media (TCMs), Trioctanoin.)
344. Megavitamins
345. Melatonin (Scientific name: N-Acetyl-5-Methoxytryptamine. Also known as: 5-Methoxy-N-Acetyltryptamine, MEL, Melatonina, Mélatonine, MLT, N-Acétyl-5-Méthoxytryptamine, Pineal Hormone.)
346. Mental Healing
347. Meridian System
348. Mesotherapy
349. Metamorphic Technique
350. Micronutrients
351. Milk Thistle (Scientific name: Silybum marianum, synonym Carduus marianus. Also known as: Artichaut Sauvage, Blessed Milk Thistle, Cardo Lechoso, Cardui Mariae Fructus, Cardui Mariae Herba, Carduus Marianum, Chardon Argenté, Chardon de Marie, Chardon de Notre-Dame, Chardon Marbré, Chardon-Marie, Épine Blanche, Holy Thistle, Lady's Thistle, Lait de Notre-Dame, Legalon, Marian Thistle, Mariendistel, Mary Thistle, Our Lady's Thistle, Shui Fei Ji, Silibinin, Silybe de Marie, Silybin, Silybum, Silymarin, Silymarine, St. Mary Thistle, St. Marys Thistle.)
352. Mind–Body Medicine
353. Mineral Water
354. Mint
a. Wild Mint (Scientific name: Mentha aquatica, synonym Mentha palustris. Also known as: Baume d'Eau, Baume de Rivière, Hairy Mint, Hierbabuena, Marsh Mint, Menta del Agua, Menta del Pantano, Menta Vellosa, Menthe Aquatique, Menthe à Grenouille, Menthe Rouge, Water Mint, Yerbabuena)
b. Japanese Mint (Scientific name: Mentha canadensis, synonym Mentha arvensis var. piperascens. Also known as: American Corn Mint, Bakha, Brook Mint, Canadian Mint, Chinese Mint, Chinese Mint Oil, Corn Mint, Cornmint, Cornmint Oil, Field Mint Oil, Huile de Menthe, Huile de Menthe des Champs, Japanese Oil of Peppermint, Menta Japonesa, Mentha Arvensis Aetheroleum, Menthe du Canada, Menthe des Champs, Menthe Japonaise, Mint Oil, Minzol, Poleo, Pudina, Putiha.)
c. Spearmint (Scientific name: Mentha spicata, synonyms Mentha viridis, Mentha cordifolia, Mentha crispa. Also known as: Curled Mint, Fish Mint, Garden Mint, Green Mint, Hierbabuena, Huile Essentielle de Menthe Verte, Lamb Mint, Mackerel Mint, Menta Verde, Menthe Crépue, Menthe Douce, Menthe à Épis, Menthe Frisée, Menthe des Jardins, Menthe Romaine, Menthe Verte, Native Spearmint, Oil of Spearmint, Our Lady's Mint, Pahari Pudina, Putiha, Sage of Bethlehem, Spearmint Essential Oil, Spire Mint, Yerba Buena, Yerbabuena.)
d. English Horsemint (Scientific name: Mentha longifolia, synonym Mentha sylvestris. Also known as: Biblical Mint, Menta de Caballo, Menthe Anglaise, Menthe Argentée, Menthe Blanche, Menthe Chevaline, Menthe à Longues Feuilles, Menthe Sauvage, Menthe Sylvestre, Pudina, Wild Mint.)
e. Peppermint (Scientific name: Mentha x piperita, synonym Mentha lavanduliodora. Also known as: Black Peppermint, Brandy Mint, Extract of Mentha Piperita, Extract of Peppermint, Extract of Peppermint Leaves, Extrait de Feuilles de Menthe de Poivrée, Extrait de Mentha Piperita, Extrait de Menthe Poivrée, Feuille de Menthe Poivrée, Field Mint, Herba Menthae, Huile de Mentha Piperita, Huile de Menthe Poivrée, Huile Essentielle de Menthe Poivrée, Lamb Mint, M. Balsamea, M. Balsamea Wild Extract, Menta Pepperita, Menta Piperita, Mentha Balsamea, Mentha Oil, Mentha Piperita Extract, Mentha Piperita Oil, Menthae Piperitae Aetheroleum, Menthae Piperitae Folium, Menthe, Menthe Poivrée, Menthol, Mint, Mint Balm, Oil of Peppermint, Paparaminta, Peppermint Essential Oil, Peppermint Extract, Peppermint Leaf, Peppermint Leaf Extract, Peppermint Oil, Western Peppermint.)
355. Mistletoe
a. American Mistletoe (Scientific name: Phoradendron leucarpum, synonyms Phoradendron avescens, Phoradendron serontium, Viscum leucarpum, Viscum avescens; Phoradendron macrophyllum; Phoradendron tomentosum. Also known as: Eastern Mistletoe, Gui Américain, Gui de Chêne, Mistletoe, Muérdago Americano.)
b. European Mistletoe (Scientific name: Viscum album. Also known as: All-Heal, Banda, Birdlime Mistletoe, Blandeau, Bois de Sainte-Croix, Bouchon, Devil's Fuge, Drudenfuss, Eurixor, Guérit-Tout, Gui, Gui Blanc, Gui Blanc d'Europe, Gui des Feuillus, Gui d'Europe, Gui Européen, Helixor, Herbe de Chèvre, Hexenbesen, Hurchu, Iscador, Isorel, Leimmistel, Mistlekraut, Mistletein, Mistletoe, Muérdago Europeo, Mystyldene, Nid de Sorcière, Pain de Biques, Rini, Verquet, Vert-Bois, Vert de Pommier, Visci, Vogelmistel, Vysorel.)
356. Molybdenum (Scientific name: Mo, Atomic number 42. Also known as: Ammonium Molybdate, Chélate de Molybdate, Chelated Molybdenum, Citrate de Molybdène, Etrathiomolybdate, Ionic Molybdenum, Molibdeno, Molybdate d'Ammonium, Molybdate de Sodium, Molybdene, Molybdène, Molybdenum Citrate, Molybdenum Picolinate, Sodium Molybdate.)
357. Morita Therapy
358. Movement Therapy
359. Moxibustion (Also known as: Aconite Cake-Separated Moxibustion, Acu-Moxi, Acu-Moxibustion, Angelica-Cake Moxibustion, Bird-Pecking Moxibustion, Chinetsukyu (Japanese), Cake-Separated Mild-Warm Moxibustion, Cake-Separated Moxibustion, Circling Moxibustion, Cone Moxibustion, Cotton Sheet Moxibustion, Crude Herb Moxibustion, Direct Cone Moxibustion, Dogbi (ST35) & Sulan Moxibustion, Drug-Separated Moxibustion, Du-Moxibustion, Dynamic Moxibustion, Electronic Moxibustion, Electrothermal Bian-Stone Moxibustion, Garlic Moxibustion, Ginger-Partitioned Moxibustion, Ginger-Salt-Partitioned Moxibustion, Grain-Shaped Moxibustion, Hand Moxibustion, Hanging Moxibustion, Heat-Sensitive Moxibustion, Herb-Cake Seperated Moxibustion, Herb-Partitioned Spread Moxibustion, Herbal-Moxa Moxibustion, Infrared Laser Moxibustion, Isolated Moxibustion, Isolated-Herbal Moxibustion, Kyutoshin (Japanese), Long Snake Moxibustion, Medicated Thread Moxibustion, Medicated Threads Moxibustion of Zhuang Nationality, Mild Moxibustion, Mild-Warm Moxibustion, Monkshood Cake- Separated Mild-Warm Moxibustion, Moving Moxibustion, Moxa, Okyu (Japanese), Partition-Bran Moxibustion, Partition-Herb Moxibustion, Pecking Moxibustion, Rice-Sized Direct Moxa, Snake Moxibustion, Solar-Term Moxibustion, Sparrow-Pecking Moxibustion, Substance-Partitioned Moxibustion, Suspended Moxibustion, Thin Cotton Moxibustion, Tortoise-Shell Moxibustion, Traditional Box Moxibustion, Warming Moxibustion, Warming Needle Moxibustion, Warming-Cup Moxibustion.)
360. Mud Therapy (Also known as: Peloid.)
361. Mudras
362. Mugwort (Scientific name: Artemisia vulgaris. Also known as: Altamisa, Armoise, Armoise Citronnelle, Armoise Commune, Armoise Vulgaire, Artémise, Artemisia, Artemisiae Vulgaris Herba, Artemisiae Vulgaris Radix, Carline Thistle, Felon Herb, Gemeiner Beifuss, Herbe aux Cent Goûts, Herbe de Feu, Herbe de la Saint-Jean, Herbe Royale, Hierba de San Juan, Nagadamni, Remise, Sailor's Tobacco, St. John's Plant, Tabac de Saint-Pierre, Wild Wormwood.)
363. Mulberry Leaf Extract
364. Mullein (Scientific name: Verbascum densiflorum; Verbascum phlomides; Verbascum thapiforme; Verbascum thapsus. Also known as: Aaron's Rod, Adam's Flannel, American Mullein, Beggar's Blanket, Blanket Herb, Blanket Leaf, Bouillon Blanc, Bouillon Jaune, Candleflower, Candlewick, Cierge Cotonneux, Cierge de Notre-Dame, Clot-Bur, Clown's Lungwort, Cuddy's Lungs, Duffle, European Mullein, Faux Bouillon-Blanc, Feltwort, Flannelflower, Fleur de Grand Chandelier, Fluffweed, Gidar Tamaku, Gordolobo, Hag's Taper, Hare's Beard, Hedge Taper, Herbe de Saint-Fiacre, Herbe Saint Fiacre, Higtaper, Jacob's Staff, Longwort, Molène, Molène à Grandes Fleurs, Molène Bouillon-Blanc, Molène Faux-Phlomis, Molène Thapsus, Orange Mullein, Oreille de Loup, Oreille de Saint Cloud, Our Lady's Flannel, Queue de Loup, Rag Paper, Shepherd's Club, Shepherd's Staff, Tabac du Diable, Torch Weed,Torches, Velvet Plant, Verbasci Flos, Wild Ice Leaf, Woolen, Woolly Mullein.)
365. Music Therapy (Also known as: Active Music Therapy, Calming Music Therapy, Contingent Music, Dinner Music Intervention, Evocative Music, Expressive Therapy, Group Chanting And Singing, Group Drumming, Guided Imagery and Music (GIM), Improvisational Music Therapy, Individualized Music-Focused Auditory Therapy (IMAT), Instructional Music Therapy, Interactive Music Therapy, Karaoke Therapy, Live Music Therapy, Lullaby Therapy, Lyric Analysis, Mandalas, Medical Resonance Therapy Music (MRT- Music), MT, Music And Movement, Music-Assisted Progressive Muscle Relaxation, Music-Assisted Reframing, Music-Based Imagery, Music-Based Intervention, Music Exposure Therapy, Music In Therapy, Music Intervention, Music Listening Intervention, Music Stimulation, Music Therapy, Music-Reinforced Therapy, Music-Video Therapy, Musical Games, Musical Motor Feedback (MMF), Musicokinetic Therapy, Orff-Based Music Therapy, Ragas.)
366. Myofascial Release (Also known as: MFR Therapy, Myotherapy, Soft Tissue Mobilization.)
367. Myotherapy
368. Myrrh (Scientific name: Commiphora myrrha, synonyms Commiphora molmol, Balsamodendrum myrrha; Commiphora habessinica, synonyms Commiphora abyssinica, Balsamodendrum habessinicum; Commiphora madagascariensis; Commiphora kataf, synonyms Commiphora erythraea, Amyris kataf, Hemprichia erythraea; other Commiphora species. Also known as: Abyssinian Myrrh, African Myrrh, Arabian Myrrh, Bal, Balsamodendron Myrrha, Bdellium, Bol, Bola, Commiphora, Common Myrrh, Didin, Didthin, Gomme de Myrrhe, Gum Myrrh, Heerabol, Mirra, Mirrh, Mo Yao, Murrah, Myrrh Gum, Myrrha, Myrrhe, Myrrhe Africaine, Myrrhe Amère, Myrrhe d'Arabie, Myrrhe Bisabol, Myrrhe Douce, Myrrhe de Somalie, Myrrhe du Yémen, Opopanax, Resina Commiphorae, Somalien Myrrh, Yemen Myrrh.)
369. N-Acetylcysteine
370. Nambudripad's Allergy Elimination Therapy
371. Napratherapy
372. Nasal Irrigation (Also known as: Hypertonic Saline Rinse, Irrigación Nasal, Irrigation Nasale, Jala Neti, Lavage Nasal, Nasal Rinsing, Nasal Saline Irrigation, Neti Pot, Nose Bidet, Pot de Neti, Saline Irrigation, Saline Nasal Irrigation, Sinus Flush, Sinus Rinse, Sinus Rinsing, SNI.)
373. Natural Medicine
374. Nature Therapy
375. Naturopathy (Also known as: Médecin Naturopathe, Médecine Naturopathique, Naturopath, Naturopathe, Naturopathic, Naturopathic Doctor, Naturopathic Medical Doctor, Naturopathic Medicine, Naturopathic Physician, Naturopathie, Naturopathique, Naturopatía, ND.)
376. Nettle
a. a. Stinging Nettle (Scientific name: Urtica dioica, Urtica urens. Also known as: Bichu, Common Nettle, Feuille d'Ortie, Graine d'Ortie, Grande Ortie, Great Stinging Nettle, Nettle, Nettle Leaf, Nettle Seed, Nettle Worth, Nettles, Ortie, Ortie Brûlante, Ortie des Jardins, Ortie Dioïque, Ortie Méchante, Ortiga, Small Nettle, Stinging Nettles, Urtica, Urticae Herba et Folium, Urticae Radix.)
b. b. White Dead Nettle Flower (Scientific name: Lamium album. Also known as: Archangel, Archangélique, Bee Nettle, Blind Nettle, Deaf Nettle, Dumb Nettle, Herbe Archangélique, Lamier Blanc, Lamii Albi Flos, Ortie Blanche, Ortie Folle, Ortie Molle, Ortie Morte, Ortiga Blanca, Ortiga Muerta, Stingless Nettle, White Archangel, White Nettle.)
377. Neural Therapy (Also known as: Interference Eld, Interference Zone.)
378. Neuromuscular Therapy
379. New Age Therapy
380. Noni (Scientific name: Morinda citrifolia. Also known as: Ba Ji Tian, Bois Douleur, Canarywood, Cheese Fruit, Hai Ba Ji, Hawaiian Noni, Hog Apple, Indian Mulberry, Indian Noni, Jus de Noni, Luoling, Mengkudu, Menkoedoe, Mora de la India, Morinda, Mulberry, Mûre Indienne, Nhau, Noni Juice, Nono, Nonu, Pau-Azeitona, Rotten Cheese Fruit, Ruibarbo Caribe, Tahitian Noni Juice, Ura, Wild Pine, Wu Ning, Yor.)
381. Non-Mainstream Medicine
382. Non-Orthodox Practice
383. Non-Pharmacological Intervention
384. Non-Traditional Medicine
385. Nuad Bo Rarn
386. Nutraceuticals
387. Nutrition Therapy
388. Oatmeal Bath
389. Ojeok-San
390. Oleander (Scientific name: Nerium oleander, synonyms Nerium indicum, Nerium odorum, Thevetia peruviana, synonyms Cascabela thevetia, Cerbera thevetia, Thevetia neriifolia. Also known as: Adelfa, Baladre, Common Oleander, Exile Tree, Huang Hua Jia, Jia Zhu Tao, Kaner, Karvir, Karvira, Laurel Rosa, Laurier-Rose, Laurier Rose, Laurose, Lorier Bol, Nérier à Feuilles de Laurier, Nérion, Oleanderblatter, Oléandre, Oleandri Folium, Rose Bay, Rose Laurel, Soland, Sweet Scented Oleander, Yellow Oleander.)
391. Olive (Scientific name: Olea europaea. Also known as: Acide Gras Insaturé, Acide Gras Mono-Insaturé, Acide Gras n-9, Acide Gras Oméga 9, Common Olive, Extra Virgin Olive Oil, Feuille d'Olivier, Green Olive, Huile d'Assaisonnement, Huile d'Olive, Huile d'Olive Extra Vierge, Huile d'Olive Vierge, Jaitun, Manzanilla Olive Fruit, Monounsaturated Fatty Acid, N-9 Fatty Acid, Oleae Folium, Olivae Oleum, Olive Fruit, Olive Fruit Pulp, Olive Leaf, Olive Oil, Olive Pulp, Olives, Olivo, Omega-9 Fatty Acids, Pulpe d'Olive, Salad Oil, Sweet Oil, Unsaturated Fatty Acid, Virgin Olive Oil.)
392. Oregon Grape (Scientific name: Mahonia aquifolium, synonyms Berberis aquifolium, Berberis diversifolia, Mahonia diversifolia; Mahonia nervosa, synonym Berberis nervosa; Mahonia repens, synonyms Berberis repens, Berberis sonnei, Mahonia sonnei. Also known as: Barberry, Berberis, Blue Barberry, Creeping Barberry, Holly Barberry, Holly Mahonia, Holly-Leaved Berberis, Mahonia, Mahonia Faux Houx, Mahonia à Feuilles de Houx, Mahonie, Mountain-Grape, Oregon Barberry, Oregon Grape-Holly, Scraperoot, Trailing Mahonia, Uva de Oregon, Vigne de l'Oregon, Water-Holly.)
393. Organic Food (Also known as: Alimentation Biologique, Alimentos Orgánicos, Green Labels, National Organic Program, Natural Food, NOP, OFPA, Organic Farming, Organic Foods Production Act, USDA Organic.)
394. Organotherapy
395. Ortho-Molecular Medicine
396. Osteopathic Medicine (Also known as: Osteopathic Manipulative Treatment, Osteopathy, Joint Manipulation, Musculoskeletal Manipulation.)
397. Otikon Otic
398. Oxygen Therapy (Also known as: HBOT, Hyperbaric Oxygen, Hyperbaric Oxygen Therapy, Medical Oxygen Therapy, Oxymedicine, Hyperbaric Oxygen, Hyperoxigenation Therapy, Bio-Oxidative Therapy)
399. Ozone Therapy (Also known as: Cure d'Ozone, Medical Ozone, Ozonated Autohemotherapy, Ozone, Ozone Médical, Ozone Therapists, Ozone Treatment, Ozonetherapy, Ozonoterapia, Ozonothérapie, Thérapeute par l'Ozone, Therapeutic Ozone, Thérapie à l'Ozone, Thérapie par l'Ozone.)
400. Paleo Diet (Also known as: Caveman Diet, Hunter-Gatherer Diet, Paleo Diet, Paleolithic Diet, Stone Age Diet.)
401. Palmistry
402. Palo Santo (Scientific name: Bursera graveolens.)
403. Panchakarma
404. Pancreatic Extract
405. Passion Flower (Scientific name: Passiflora incarnata. Also known as: Apricot Vine, Burucuya, Corona de Cristo, Fleischfarbige, Fleur de la Passion, Fleur de Passiflore, Flor de Passion, Granadilla, Grandilla, Grenadille, Madre Selva, Maracuja, Maracuya, Maypop, Maypop Passion Flower, Pasiflora, Pasionari, Pasionaria, Passiflora, Passiflorae Herba, Passiflore, Passiflore Aubépine, Passiflore, Passiflore Officinale, Passiflore Purpurine, Passiflore Rouge, Passiflorina, Passion Vine, Passionaria, Passionblume, Passionflower, Passionflower Herb, Passionsblomma, Passionsblumenkraut, Purple Passion Flower, Water Lemon, Wild Passion Flower.)
406. Passion Fruit (Scientific name: Passiflora edulis.)
407. Past Life Therapy
408. Pastoral Care
409. Pau d'Arco (Scientific name: Tabebuia impetiginosa, synonyms Handroanthus impetiginosus, Tabebuia avellanedae, Tabebuia heptaphylla, Tabebuia palmeri, Tecoma impetiginosa. Also known as: Bénier de Guyane, Ébène Vert, Ipe, Ipe Roxo, Ipes, Lapacho, Lapacho Colorado, Lapacho Morado, Lébène, Pink Trumpet Tree, Purple Lapacho, Quebracho, Red Lapacho, Taheebo, Taheebo Tea, Thé Taheebo, Trumpet Bush.)
410. PC-SPES
411. Pectin (Scientific name: Pectin. Also known as: Acide Pectinique, Acide Pectique, Apple Pectin, Citrus Pectin, Fractionated Pectin, Fruit Pectin, Grapefruit Pectin, Lemon Pectin, MCP, Modified Citrus Pectin, Pectina, Pectine, Pectine d'Agrume, Pectine d'Agrume Modifiée, Pectine de Citron, Pectine de Fruit, Pectine de Pamplemousse, Pectine de Pomme, Pectinic Acid.)
412. Pennyroyal (Scientific name: Mentha pulegium, synonym Pulegium vulgare; Hedeoma pulegioides, synonym Melissa pulegioides. Also known as: American Pennyroyal, Dictame de Virginie, European Pennyroyal, Feuille de Menthe Pouliot, Frétillet, Herbe aux Puces, Herbe de Saint-Laurent, Huile de Menthe Pouliot, Lurk-In-The-Ditch, Menthe Pouliot, Menthe Pouliote, Mosquito Plant, Piliolerial, Poleo, Pouliot, Pouliot Royal, Pudding Grass, Pulegium, Run-By-The-Ground, Squaw Balm, Squawmint, Stinking Balm, Tickweed.)
413. Pennywort
a. Gotu Kola (Scientific name: Centella asiatica, synonym Hydrocotyle asiatica; Centella coriacea. Also known as: Asiatic Pennywort, Brahma-Buti, Brahma-Manduki, Brahmi, Bua-Bok, Centella, Centella Asiática, Centella Asiatique, Centellase, Divya, Hydrocotyle, Hydrocotyle Asiatique, Hydrocotyle Indien, Indischer Wassernabel, Idrocotyle, Indian Pennywort, Indian Water Navelwort, Ji Xue Cao, Khulakhudi, Luei Gong Gen, Luo De Da, Madecassol, Mandukaparni, Manduk Parani, Mandukig, Marsh Penny, TECA, TTFCA, Talepetrako, Thick-Leaved Pennywort, Tiger Grass, Tsubo-kusa, Tungchian, White Rot.)
b. Bacopa (Scientific name: Bacopa monnieri, synonym Bacopa monniera, Herpestis monniera, Moniera cuneifolia. Also known as: Andri, Bacopa, Brahmi, Herb of Grace, Herpestis Herb, Hysope d'Eau, Indian Pennywort, Jalanimba, Jal-Brahmi, Jalnaveri, Nira-Brahmi, Sambrani Chettu, Thyme-Leaved Gratiola, Water Hyssop.)
414. Peony (Scientific name: Paeonia lactiflora, synonym Paeonia albiflora, Paeonia mascula, synonyms Paeonia arietina, Paeonia caucasica, Paeonia corallina, Paeonia coriacea, Paeonia daurica, Paeonia kavachensis, Paeonia triternata, Paeonia obovata, synonyms Paeonia japonica, Paeonia obovata, Paeonia willmottiae, Paeonia officinalis, synonyms Paeonia microcarpa, Paeonia paradoxa, Paeonia suffruticosa, synonyms Paeonia arborea, Paeonia moutan, Paeonia anomala, synonyms Paeonia veitchii, Paeonia beresowskii, Paeonia woodwardii. Also known as: Bai Shao, Chi Shao, Chinese Peony, Common Peony, Coral Peony, Cortex Moutan, European Peony, Jiu Chao Bai Shao, Moutan, Mu Dan Pi, Paeoniae Alba, Paeoniae Flos, Paeoniae Radix, Peonía, Peony Root, Piney, Pivoine, Pivoine Arbustive, Pivoine Blanche, Pivoine Commune, Pivoine de Chine, Pivoine des Jardins, Pivoine en Arbre, Pivoine Moutan, Pivoine Officinale, Pivoine Rouge, Racine de Pivoine, Radix Paeoniae, Radix Paeoniae Rubra, Radix Peony, Red Peony, Shakuyaku, Shao Yao, Tree Peony, Ud Saleeb, Udsalam, Udsalap, White Peony.)
415. Phenylalanine (Scientific name: 2-amino-3-phenyl-propanoic acid. Also known as: Acide Alpha-aminohydrocinnamique, Acide Isovalérique de Phénylalanine, Alpha aminohydrocinnamic Acid, Beta-phenyl-alanine, Bêta-phenyl-alanine, DLPA, D-Phenylalanine, D Phénylalanine, DL-Phenylalanine, DL-Phénylalanine, D,LPhenylalanine, D,L-Phénylalanine, Fenilalanina, L-Phenylalanine, L-Phénylalanine, Phenylalanine Ethyl Ester HCl, Phenylalanine Isovaleric Acid, Phenylalanine Methyl Ester HCl.)
416. Phosphatidyl Choline
417. Phosphatidylserine (Also known as: BC-PS, Bovine Cortex Phosphatidylserine, Fosfatidilserina, LECI-PS, Lecithin Phosphatidylserine, Phosphatidylsérine, Phosphatidylsérine Bovine, Phosphatidylsérine de Soya, Phosphatidyl Serine, PS, PtdSer, Soy-PS, Soy Phosphatidylserine.)
418. Physical Therapy (Also known as: Physical Manipulation, Physical Medicine, Manupulative Therapy, Soft Tissue Manipulation.)
419. Phytotherapy (Also known as: Phytomedicine, Phytoestrogen, Phytoestrol, Phytonutrient, Phytopharma, Phytosterol.)
420. Pilates (Also known as: Contrologie, Contrology, Méthode Pilates.)
421. Plant-Based Medicines (Also known as: Floral Therapy, Pine-Bark Extract, Placebo Plant, Plant Extracts, Plant Oils, Plant-Based Medications.)
422. Play Therapy
423. Polarity Therapy (Also known as: Energy Medicine, Energy Therapy, Energy Work, Médecine Énergétique, Polarité, Polarity, Polarity Balancing, Polarity Therapist, Polarity Treatment, Terapia de Polaridad, Thérapeute en Polarité, Thérapie Énergétique, Thérapie de l'Énergie, Thérapie de Polarité, Thérapie par la Polarité.)
424. Pomegranate (Scientific name: Punica granatum. Also known as: Anardana, Dadim, Dadima, Delima, Extrait de Feuille de Grenade, Extrait de Grenade, Extrait de Polyphénol de Grenade, Feuille de Grenade, Fleur de Grenade, Fruit du Grenadier, Fruit of the Dead, Gangsalan, Granaatappel, Granad, Granada, Granado, Granatapfel, Grenade, Grenadier, Limoni, Melogranato, Melograno Granato, PE, PLE, Pomegranate Extract, Pomegranate Flower, Pomegranate Fruit, Pomegranate Leaf, Pomegranate Leaf Extract, Pomegranate Polyphenol Extract, Pomme Grenade, Pomo Granato, Pomo Punico, PPE, Roma, Romazeira, Romeira, Shi Liu Gen Pi, Shi Liu Pi, Tab Tim.)
425. Positive Intention Practice
426. Postural Realignment
427. Potassium (Scientific name: K, Atomic number 19. Also known as: Acétate de Potassium, Bicarbonate de Potassium, Chlorure de Potassium, Citrate de Potassium, Gluconate de Potassium, Glycérophosphate de Potassium, Numéro Atomique 19, Orotate de Potassium, Phosphate de Potassium, Potasio, Potassium Acetate, Potassium Ascorbate, Potassium Bicarbonate, Potassium Chloride, Potassium Citrate, Potassium Gluconate, Potassium Glycerophosphate, Potassium Hydroxide, Potassium Orotate, Potassium Phosphate, Potassium Sulfate, Sulfate de Potassium.)
428. Pranic Healing
429. Prayer, Distant Healing (Also known as: Faith Healing, Spiritual Therapy, Christian Science, Mysticism, Absent Healing, Attitudinal Healing, Centering Prayer, Compassion And Healing, Compassionate Intention, Distant Healing, Divining, External Qigong, Faith Healing, Intentionality, Intercessory Prayer (IP), Kahuna Healing, Native American Faith Healing, Noetic Therapy, Psychic Healing, Quantum Healing, Reiki, Religion, Remote Healing, Remote Prayer, Spiritual Healing, Sufi Healing.)
430. Prebiotics
431. Preventative Medicine
432. Primitive Medicine
433. Pritkin Diet
434. Probiotics
435. Progressive Muscular Relaxation (Also known as: Progressive Relaxation.)
436. Prolotherapy (Also known as: Nonsurgical Reconstructive Therapy, Proliferative Therapy, Reconstructive Therapy.)
437. Propolis (Scientific name: Propolis. Also known as: Acide de Cire d'Abeille, Baume de Propolis, Bee Glue, Bee Propolis, Beeswax Acid, Brazilian Green Propolis, Brazilian Propolis, Brown Propolis, Cire d'Abeille Synthétique, Cire de Propolis, Colle d'Abeille, Green Propolis, Hive Dross, Pénicilline Russe, Propóleos, Propolis Balsam, Propolis Cera, Propolis d'Abeille, Propolis Resin, Propolis Wax, Red Propolis, Résine de Propolis, Russian Penicillin, Synthetic Beeswax, Yellow Propoli.)
438. Protein Diet
439. Protein Restricted Diet
440. Psychic Medicine (Also known as: Psychic Healing.)
441. Psychoneuroimmunology
442. Psychotherapy (Also known as: Psychodrama.)
443. Psyllium
a. Black Psyllium (Scientific name: Plantago arenaria, synonym Plantago psyllium, Plantago afra, Plantago indica, Psyllium arenarium, Psyllium indica. Also known as: frican Plantain, Brown Psyllium, Dietary Fiber, Erva-das-Pulgas, Fibre Alimentaire, Fleaseed, Fleawort, Flohkraut, Flohsamen, French Psyllium, Glandular Plantain, Graine de Psyllium, Herbes aux Puces, Œil-de-Chien, Pilicaire, Plantain, Plantain Pucier, Psyllii Semen, Psyllion, Psyllios, Psyllium, Psyllium Brun, Psyllium d'Espagne, Psyllium Noir, Psyllium Seed, Pucière, Pucilaire, Scharzer Flohsame, Spanish Psyllium, Zaragatona.)
b. Blonde Psyllium (Scientific name: Plantago ovata, synonyms Plantago fastigiata, Plantago insularis, Plantago ispaghula, Plantago decumbens. Also known as: Balle de Psyllium, Blond Plantago, Blonde Psyllium, Che Qian Zi, Dietary Fiber, Englishman's Foot, Fibre Alimentaire, Indian Plantago, Ipágula, Isabgola, Isabgul, Ispaghul, Ispaghula, Ispagol, Pale Psyllium, Plantaginis Ovatae Semen, Plantaginis Ovatae Testa, Psilio, Psillium Blond, Psyllium, Psyllium Blond, Psyllium Husk, Sand Plantain, Spogel.)
444. Puerarin
445. Pulsed Therapy
446. Pumpkin Seed
447. Purple Sweet Potato (Also known as: Purple Yam.)
448. Pygeum (Scientific name: Prunus africana, synonym Pygeum africanum. Also known as: African Plum Tree, African Prune, African Pygeum, Amande Amère, Ciruelo Africano, Prunier d'Afrique, Pygeum Africanus.)
449. Pyrrolizidine Alkaloid
450. Qi Gong (Also known as: Ba Duan Jin, Baduanjin Qi Gong, Biyun Method, Biyun Qi Gong, Chan Mi Gong, Chi'I Kung, Chi Gung, Chi Kung, Dantian, Daoyin Qi Gong, Dongeui Qi Gong, Eight-Section Brocades Qi Gong, Energy Healing, Energy Health, Energy Medicine, Energy Therapy, Energy Work, EQT, External Qi Gong Therapy, Five Animals Qi Gong, Guolin, Hua Gong, Medical Qi Gong, Médecine Énergétique, QG, QI, Qigong, Qigong Healer, Qigong Therapy, Qi Gong Therapy, Qi Gong Yangsheng, Qigongology, Santé Énergétique, Thérapie Énergétique, Wuqinxi, Yi Jia Gong.)
451. Quantum Healing (Also known as: Quantum Medicine.)
452. Radiance Technique
453. Radiation Therapy
454. Radiesthesia
455. Radisthesis
456. Rapid Eye Technology
457. Rebirthing
458. Red Clover (Scientific name: Trifolium pratense. Also known as: Beebread, Clovone, Cow Clover, Daidzein, Genistein, Isoflavone, Meadow Clover, Miel des Prés, Phytoestrogen, Purple Clover, Trebol Rojo, Trèfle Commun, Trèfle des Prés, Trèfle Pourpre, Trèfle Rouge, Trèfle Rougeâtre, Trèfle Violet, Trefoil, Trifolium, Wild Clover.)
459. Red Yeast Rice (Scientific name: Monascus purpureus; other Monascus species. Also known as: Arroz de Levadura Roja, Cholestin, Hong Qu, Hongqu, Koji Rouge, Levure de Riz Rouge, Mevinolin, Monacolin K, Monascus, Monascus Purpureus Went, Red Koji, Red Rice, Red Rice Yeast, Red Yeast Rice, Red Yeast Rice Extract, Riz Rouge, Rotschimmelreis, XueZhiKang, Xue Zhi Kang, XZK, Zhibituo, Zhitai, Zhi Tai.)
460. Reflex Locomotion
461. Reflexology (Also known as: Bodywork Therapy, Ear Reflexology, Energy Health, Energy Medicine, Energy Work, Foot Reflexology, Foot Therapy, Hand Reflexology, Lymphatic Reflexology, Massage Réflexologique, Médecine Énergétique, Reflexología, Réflexologie, Réflexologie Auriculaire, Réflexologie Plantaire, Reflexologist, Réflexologue, Reflexotherapy, Réflexothérapie, Santé Énergétique, Thérapie de Zone, Zone Therapy.)
462. Regression Therapy
463. Reichian
464. Reiki Therapy (Also known as: Bioenergy Therapy, Biofield Energy Therapy, Buddhist Reiki, Énergie Universelle de Vie, Energy Health, Energy Medicine, Energy Work, Healing Touch, Japanese Reiki, Médecine Énergétique, Ray-kee, Reiki, Reiki Japonais, Reiki Therapie, Reiki Touch Therapy, Terapia Reiki, Therapeutic Touch, Thérapie Manuelle Énergétique, Thérapie par le Toucher, Touch Therapy, Toucher Guérisseur, Toucher Thérapeutique, Universal Life Energy.)
465. Reishi Mushroom (Scientific name: Ganoderma lucidum. Also known as: Basidiomycetes Mushroom, Champignon Basidiomycète, Champignon d'Immortalité, Champignon Reishi, Champignons Reishi, Ganoderma, Hongo Reishi, Ling Chih, Ling Zhi, Mannentake, Mushroom, Mushroom of Immortality, Mushroom of Spiritual Potency, Red Reishi, Reishi, Reishi Antler Mushroom, Reishi Rouge, Rei-Shi, Spirit Plant.)
466. Relaxation Therapy (Also known as: Abbreviated Muscle Relaxation Therapy, Applied Relaxation, Relaxation Appliquée, Relaxation Training, Relaxation Treatment, RT, Terapia de Relajación, Therapeutic Relaxation, Thérapie de Relaxation, Traitement de Relaxation, Relaxation Classes, Relaxation Response, Relaxation Response Therapy, Relaxation Tapes, Relaxation Techniques, Guided Relaxation.)
467. Religious Therapy (Also known as: Religion and Medicine, Religious Healing.)
468. Restricted Environmental Stimulation Therapy
469. Resveratrol (Scientific name: 3,4',5-stilbenetriol, 3,5,4'-trihydroxystilbene, 3,4',5-trihydroxystilbene, 3,5,4'-trihydroxy-trans-stilbene. Also known as: 3,5,4' TriHydroxy-Transstibene, (E)-5-(4-hydroxystyryl)benzene-1,3-diol, Cis-Resveratrol, Extrait de Vin, Extrait de Vin Rouge, Kojo-Kon, Phytoalexin, Phytoalexine, Phytoestrogen, Phyto-œstrogène, Pilule de Vin, Protykin, Red Wine Extract, Resvératrol, Resveratrols, Resvératrols, RSV, RSVL, Stilbene Phytoalexin, Trans-Resveratrol, Trans-Resvératrol, Wine Extract, Wine Pill)
470. Revici Method
471. Rhodiola (Scientific name: Rhodiola rosea, synonyms Sedum rhodiola, Sedum rosea. Also known as: Arctic Root, Extrait de Rhodiole, Golden Root, Hongjingtian, Hong Jing Tian, King's Crown, Lignum Rhodium, Orpin Rose, Racine d'Or, Racine Dorée, Racine de Rhadiola, Rhodiole, Rhodiole Rougeâtre, Rodia Riza, Rose Root, Rose Root Extract, Rosenroot, Roseroot, Rosewort, Siberian Golden Root, Siberian Rhodiola Rosea, Snowdown Rose.)
472. Rice Bran (Scientific name: Oryza sativa. Also known as: Brown Rice Bran, Cereal Fiber, Dietary Fiber, Fibre Alimentaire, Fibre Céréalière, Huile de Son de Riz, Rice Bran Oil, Ricebran Oil, Riz de Son, Salvado de Arroz, Son de Riz, Stabilized Rice Bran.)
473. Rolfing (Also known as: Intégration Structurale par le Rolfing, Intégration Structurelle, Manipulative Therapy, Médecine Physique, Méthode Rolfing, Physical Medicine, Rolfer, Rolfing Method, Rolfing Structural Integration, Rolfing Therapy, Thérapie Manuelle.)
474. Rongoa
475. Rosen Method
476. Rubenfeld Synergy
477. Sacred Healing
478. Safflower Yellow Injection
479. Saffron (Scientific name: Crocus sativus. Also known as: Autumn Crocus, Azafrán, Azafron, Croci Stigma, Crocus Cultivé, Indian Saffron, Kashmira, Kesar, Kumkuma, Saffron Crocus, Safran, Safran Cultivé, Safran Espagnol, Safran des Indes, Safran Véritable, Spanish Saffron, True Saffron, Zafran.)
480. Salacia (Scientific name: Salacia oblonga; Salacia reticulata. Also known as: Chundan, Kothala Himbutu, Ponkoranti, SO, S. oblonga.)
481. Salvia divinorum (Also known as: Divine Mexican Mint, Diviner's Mint, Diviners Sage, Divinorin, Divinorin A, Feuilles de la Bergère, Feuilles de la Vierge, Herb-of-the-Virgin, Herb of Mary, Herba de María, Hierba de la Virgen, Hierba Maria, Hojas de la Pastora, Hojas de Maria, La Hembra, Leaf of Mary, Leaves of the Virgin Shepherdess, Magic Mint, Menthe Magique, Mexican Mint, Mexican Sage, Mexican Sage Incense, Pipiltzintzintli, Sadi, Sally-D, Salvia, Salvinorin, Salvinorin A, Sage of the Seers, Sauge des Devins, Sauge Divinatoire, Shepherdess, Ska Maria, Ska Maria Pastora, Ska Pastora, Yerba de Maria, Yerba Maria.)
482. Samadhi
483. SAMe (Scientific name: S-adenosyl-L-methionine. Also known as: Ademetionine, Adenosylmethionine, Adénosylméthionine, S-Adenosyl Methionine, S-Adénosyl Méthionine, S-Adenosyl-L-Methionine, S-Adénosyl-L-Méthionine, S-Adenosylmethionine, S-Adénosylméthionine, S-Adenosylmethionine Butanedisulfonate, S-Adenosylmethionine Tosylate, S-Adenosylmethionine Tosylate Disulfate, SAM, SAM-e, Sammy, Samyr.)
484. Samyama
485. Sanchi Preparation
486. Sand Bath
487. Sauna
488. Saw Palmetto (Scientific name: Serenoa repens, synonyms Serenoa serrulata, Sabal serrulata. Also known as: American Dwarf Palm Tree, Baies du Chou Palmiste, Baies du Palmier Scie, Cabbage Palm, Chou Palmiste, Ju-Zhong, Palma Enana Americana, Palmier de Floride, Palmier Nain, Palmier Nain Américain, Palmier Scie, Sabal, Sabal Fructus, Saw Palmetto Berry.)
489. Scraping (Also known as: Coining, Gua Sha, Kerokan Spooning.)
490. Seaweed
491. Sectarian Medicine
492. Selenium (Scientific name: Se, Atomic number 34. Also known as: Dioxyde de Sélénium, Ebselen, L-Selenomethionine, L-Sélénométhionine, Levure Sélénisée, Numéro Atomique 34, Selenio, Selenite, Sélénite de Sodium, Sélénium, Selenium Ascorbate, Selenium Dioxide, Selenized Yeast, Selenomethionine, Sélénométhionine, Sodium Selenite.)
493. Self Help (Also known as: Self-Awareness, Self-Care, Self-Healing Abilities, Self-Help, Self-Massage, Self-Medication.)
494. Senna (Scientific name: Senna alexandrina, synonyms Cassia acutifolia, Cassia angustifolia, Cassia senna, Cassia lanceolata. Also known as: Alexandrian Senna, Alexandrinische Senna, Casse, Fan Xie Ye, Indian Senna, Khartoum Senna, Khatoum Senna, Sen, Sena Alejandrina, Séné, Séné d'Alexandrie, Séné d'Egypte, Séne d'Inde, Séné de Tinnevelly, Sennae Folium, Sennae Fructus, Sennosides, Tinnevelly Senna, True Senna.)
495. Sensory Therapy
496. Shamanism (Also known as: Shamanic Healing.)
497. Shengmai (Also known as: Shenmai.)
498. Shenqi Fuzheng
499. Shensu (Also known as: Shenfu.)
500. Shexiang
501. Shiatsu (Also known as: Asian Bodywork, Asian Bodywork Therapy, Bodywork Therapy, Energy Health, Energy Medicine, Energy Work, Finger Pressure, Japanese Shiatsu, Médecine Énergétique, Meridian Shiatsu, Movement Shiatsu, Namikoshi Shiatsu, Ohashiatsu, Quantum Shiatsu, Santé Énergétique, Shi-astsu, Shiatsu Japonais, Shiatsu Massage, Shiatsu des Méridiens, Shiatsu Ryoho, Shiatsu Therapy, Shiatsupractor, Tao Shiatsu, Thérapeute en Shiatsu, Tsubo Shiatsu, Zen Shiatsu.)
502. Shitake Mushroom (Scientific name: Lentinus edodes, synonyms Lenticus edodes, Lentinan edodes, Lentinula edodes, Tricholomopsis edodes. Also known as: Champignon Noir, Champignon Parfumé, Champignon Shiitake, Champignons Shiitake, Forest Mushroom, Hongos Shiitake, Hua Gu, Lentin, Lentin des Chênes, Lentin du Chêne, Lentinula, Mushroom, Pasania Fungus, Shiitake, Shitake, Snake Butter, Xiang-Gu.)
503. Shuanghuanglian
504. Siddha
505. Silicon (Scientific name: Si, Atomic number 14. Also known as: Acide Orthosilicique, Dioxyde de Silicium, Numéro Atomique 14, Orthosilicic Acid, Phytolithic Silica, Polysilicone-11, Silica, Silica Hydride, Silice Hydride, Silicea, Silicio, Silicium, Silicium de Sodium, Silicon Dioxide, Sodium Silicate.)
506. Sintergetic Medicine
507. Snake Venom
508. Snoezelen (Also known as: Controlled Multisensory Environment.)
509. Social Thermalism
510. Sodium Restricted Diet
511. Somatherapy
512. Sophrology
513. Soul Retrieval
514. Sound Healing (Also known as: Vibration Healing.)
515. Soy (Scientific name: Glycine max, synonyms Glycine soja, Dolichos soja, Glycine gracilis, Glycine hispida, Phaseolus max, Soja hispida, Soja max. Also known as: Cosse de Soja, Cosse de Soya, Daidzein, Daidzéine, Edamame, Estrogène Végétal, Fermented Soy, Fève de Soja, Fève de Soya, Fibre de Soja, Fibre de Soya, Frijol de Soya, Genistein, Génistéine, Haba Soya, Haricot de Soja, Haricot de Soya, Hydrolyzed Soy Protein, Isoflavone, Isoflavone de Soja, Isoflavone de Soya, Isoflavones, Isolated Soy Protein, Isolated Soybean Protein, Lait de Soja, Lait de Soya, Legume, Miso, Natto, Phytoestrogen, Phyto-œstrogène, Plant Estrogen, Protéine de Haricot de Soja Isolée, Protéine de Haricot de Soya Isolée, Protéine de Soja, Protéine de Soya, Protéine de Soja Isolée, Protéine de Soya Isolée, Protéine de Soya Isolée, Shoyu, Soja, Sojabohne, Soy Bean, Soy Fiber, Soy Germ, Soy Isoflavone, Soy Isoflavones, Soy Milk, Soy Polysaccharide, Soy Protein, Soy Protein Isolate, Soya, Soya Bean, Soja Fermenté, Soya Fermenté, Soybean, Soybean Curd, Soybean Isoflavone, Soybean Isoflavones, Tempeh, Texturized Vegetable Protein, Tofu, Touchi.)
516. Spa
517. Speleotherapy
518. Spinal Manipulation
519. Spot Therapy
520. St. John's Wort (Scientific name: Hypericum perforatum. Also known as: Amber, Amber Touch-and-Heal, Barbe de Saint-Jean, Chasse-Diable, Demon Chaser, Fuga Daemonum, Goatweed, Hardhay, Herbe à la Brûlure, Herbe à Mille Trous, Herbe Aux Fées, Herbe Aux Mille Vertus, Herbe Aux Piqûres, Herbe de Saint Éloi, Herbe de la Saint-Jean, Herbe du Charpentier, Herbe Percée, Hierba de San Juan, Hypereikon, Hyperici Herba, Hypericum, Klamath Weed, Klamathaweed, Millepertuis, Millepertuis Perforé, Perforate St. John's Wort, Racecourse Weed, Rosin Rose, Saynt Johannes Wort, SJW, Tipton Weed.)
521. Stanol Therapy
a. Sitostanol (Scientific name: 3-beta,5-alpha-stigmastan-3-ol. Also known as: 24-alpha-ethylcholestanol, Beta-sitostanol, Bêta-sitostanol, Dihydro-beta-sitosterol, Ester de Stanol Végétal, Fucostanol, Phytostanol, Plant Stanol, Plant Stanol Esters, Stanol Végétal, Stigmastanol.)
b. Plant Sterol (Also known as: (24S)-5,22-Stigmastadien-3-beta-ol, 3-beta-stigmast-5-en-3-ol, 22,23-dihydrostigmasterol, 24-beta-ethyl-delta-5-cholesten-3-beta-ol, 24-ethyl-cholesterol, Avenasterol, B-sitosterol 3-B-D-glucoside, B-Sitosterolin, B-Sitosterols, Beta Sitosterin, Bêta-sitostérine, Beta Sitosterol, Bêta-Sitostérol, Beta-sitosterol glucoside, Beta-sitosterol glycoside, Betasitosterol, Brassicasterol, Campest-5-en-3beta-ol, Campesterol, Campestérol, Cinchol, Cupreol, Dihydro-beta-sitosterol, Ester de Stérol Végétal, Esters de Phytostérol, Esters de Stérol Dérivés d'huile Végétale, Glucoside de Bêta-Sitostérol, Phytosterol, Phytostérol, Phytosterol Esters, Phytosterols, Phytostérols, Plant Phytosterols, Plant Sterol Esters, Plant Sterolins, Quebrachol, Rhamnol, Sitosterin, Sitosterol, Sitosterolins, Sitosterols, Sterinol, Stérolines, Stérolines Végétales, Sterolins, Stérols Végétaux, Stigmasterin, Stigmasterol, Stigmastérol, Vegetable Oil Sterol Esters, Vegetable Sterol Esters.)
522. Staphage Lysate
523. Stem Cell Therapy (Also known as: Adipose Derived Stem Cell Therapy, Commercial Stem Cell Therapy, Commercial Stem Cell Transplant, Commercial Stem Cell Transplantation, Mesenchymal Stem Cell Therapy, Mesenchymal Stromal Cell Therapy, Stem Cell-Based Interventions, Stem Cell Clinics, Stem Cell Tourism, Stem Cell Treatments, SCBI.)
524. Stevia (Scientific name: Stevia rebaudiana, synonym Eupatorium rebaudianum, Stevia eupatoria, synonyms Mustelia eupatoria, Stevia purpurea. Also known as: Azucacaa, Caa-He-É, Caa'Inhem, Ca-A-Jhei, Ca-A-Yupi, Capim Doce, Chanvre d'Eau, Eira-Caa, Erva Doce, Estevia, Green Stevia, Kaa Jhee, Paraguayan Stevioside, Plante Sucrée, Reb A, Rebaudioside A, Rébaudioside A, Rebiana, Stévia, Stevia Plant, Stevioside, Sweet Herb of Paraguay, Sweet Herb, Sweet Leaf of Paraguay, Sweetleaf, Yerba Dulce.)
525. Strength Training
526. Stress Reduction
527. Stretch (Also known as: Hold Relax.)
528. Sulfinic Acid
529. Suxiao Jiuxin Wan
530. Symbolic Action (Also known as: Symbolic Chant, Symbolic Movement.)
531. Tabebuia
532. Tactile Therapy
533. Tai Chi (Also known as: Art Martial Interne, Internal Martial Art, Méditation en Mouvement, Moving Meditation, Tai-Chi, Tai Chi Chih, Tai Chi Chuan, Tai Chi Martial Arts, Taichi Quan, Tai Ji Quan, Taiji, Taijiquan, Tie Chee.)
534. Tao
535. Taurine (Scientific name: 2-Aminoethane Sulfonic Acid. Also known as: 2-Aminoethylsulfonic Acid, Acide Aminoéthylsulfonique, Acide Kétoisocaproïque de Taurine, Acid Aminoethanesulfonate, Aminoethanesulfonate, Aminoéthylsulfonique, Dibicor, Éthyl Ester de Taurine, L-Taurine, Taurina, Taurine Ethyl Ester, Taurine Ketoisocaproic Acid.)
536. Tea Tree Oil (Scientific name: Melaleuca alternifolia. Also known as: Aceite del Árbol de Té, Australian Tea Tree Oil, Huile de Melaleuca, Huile de Théier, Huile de Théier Australien, Huile Essentielle de Théier, Melaleuca Oil, Oil of Melaleuca, Oleum Melaleucae, Tea Tree, Tea Tree Essential Oil, Ti Tree Oil.)
537. Transcutaenous Electrical Nerve Stimulation (TENS) Therapy
538. Testosterone Enhancement
539. Thalassotherapy (Also known as: Hydrotherapy, Helium Thalassotherapy.)
540. Therapeutic Counsel
541. Therapeutic Crisis Intervention
542. Thermotherapy (Also known as: Heat Therapy, Therapeutic/Induced Hypothermia/Hyperthermia.)
543. Thomsonianism
544. Thought Field Therapy (Also known as: Callahan Techniques Thought Field Therapy (CTTFT), Mental Field Therapy (MFT).)
545. Thought Therapy
546. Threonine (Scientific name: L-Threonine. Also known as: L-Thréonine, Thréonine, Treonina.)
547. Thunder God Vine (Scientific name: Tripterygium wilfordii. Also known as: Huang-T'eng Ken, Lei Gong Teng, Lei-Kung T'eng, Seven Step Vine, Taso-Ho-Hua, Threewingnut, Tonnerre de la Vigne de Dieu, Tripterigium Wilfordii, Vigne du Tonnerre Divin, Yellow Vine.)
548. Thyme
a. Thyme (Scientific name: Thymus vulgaris, Thymus zygis. Also known as: Common Thyme, Farigoule, Farigoulette, French Thyme, Frigoule, Garden Thyme, Huile Essentielle de Thym, Huile de Thym, Huile de Thym Blanc, Huile de Thym Rouge, Mignotise des Genevois, Oil of Thyme, Pote, Red Thyme Oil, Rubbed Thyme, Serpolet, Spanish Thyme, Thym, Thym Citron, Thym Commun, Thym des Jardins, Thym Maraîcher, Thym Vrai, Thym Vulgaire, Thyme Aetheroleum, Thyme Essential Oil, Thyme Oil, Thymi herba, Tomillo, Van Ajwayan, Vanya Yavani, White Thyme Oil.)
b. Wild Thyme (Scientific name: Thymus serpyllum. Also known as: Breckland Thyme, Creeping Thyme, Garden Thyme, Iper, Mother of Thyme, Serpolet, Serpyllum, Shepherd's Thyme, Thym des Jardins, Thym de Bergère, Thym à Feuilles Étroites, Thym Sauvage, Thym Serpolet, Tomillo Silvestre.)
549. Thymus Extract (Also known as: Complexe de Peptides Thymiques, Extracto de Timo, Extrait de Thymus, Extrait Thymique, Polypeptides Dérivés de Thymus, Predigested Thymus Extract, Protéine Thymique, Pure Thymic Extract, Thymic Extract, Thymic Peptide, Thymic Protein, Thymic Protein A, Thymomodulin, Thymosin, Thymosine, Thymostimulin, Thymostimuline, Thymus, Thymus Acid Lysate Derivative, Thymus Complex, Thymus Concentrate, Thymus-Derived Polypeptides, Thymus Factors, Thymus Polypeptides, Thymus Substance.)
550. Tianmadingxian Capsule
551. Tinospora cordifolia (Also known as: Ambervel, Amrita, Amritha, Gilo, Giloe, Giloy, Glunchanb, Guduchi, Gulancha Tinospora, Guduchi, Gulvel, Gurcha, Heart-Leaved Moonseed, Heavenly Elixir, Indian Tinospora, Jetwatika, Mehahara, Mehaghna, Moonseed, Pramehahara, Pramehaghna, Sindal, Sittamrytu, Somida, Tinospora, Tinospora Indien, Tinosporia Cordifolus.)
552. Tissue Therapy
553. Topical Therapy
554. Touch Therapy
a. Healing Touch (Also known as: Bioenergy Healing, Biofield Therapy, Energy Healing, Energy Work, HT, Spiritual Healing, Therapeutic Touch, Touch Therapy.)
b. Therapeutic Touch (Also known as: Energy Healing, Energy Medicine, Energy Work, Médecine Énergétique, Toque Terapéutico, Touch Therapy, Toucher Thérapeutique, Toucher Guérisseur, TT.)
555. Traditional Medicine (Also known as: First Nation Tradition, Indigenous Medicine, Native American Medicine, Prophetic Medicine, Traditional Ancestral Medicine, Traditional Herbal Medicine, Traditional Asian Healing, Traditional Asian Medicine, Traditional Bhutanese Medicine, Traditional Birth Attendants, Traditional Cautery, Traditional Chinese Herbal Remedies, Traditional Chinese Medicine, Traditional Eastern And Western Medicine, Traditional European Healing Methods, Traditional European Medicine, Traditional Healer, Traditional Healing Practices, Traditional Indian Medicine, Traditional Korean Medicine, Traditional Malay Medicine, Traditional Maori Healing, Traditional Medication, Traditional Midwivery, Traditional Mongolian Medicine, Traditional Oriental Medicine, Traditional Persian Medicine, Traditional South American Medicine, Traditional Therapeutic Exercises, Traditional Tongan Medicine.)
556. Trager Therapy (Also known as: Approche Pyscho-Corporelle, Approche Trager, Manual Therapy, Massage Trager, Mentastics, Méthode d'Intégration Psyochophysique Trager, Méthode Trager, Terapia Trager, Thérapie Corporelle, Thérapie Manuelle, Thérapie de la Méthode Trager, Thérapie Trager, Trager, Trager Approach, Trager Bodywork, Trager Manipulation, Trager Mentastics, Trager Movement Therapy, Trager Psychophysical Integration Therapy, Trager Re-Education Therapy, Trager Technique, Trager Therapeutics.)
557. Transcendental Medicine (Also known as: Transcendental Meditation.)
558. Transcranial Magnetic Stimulation (Also known as: Repetitive Transcranial Magnetic Stimulation.)
559. Transpersonal Psychology
560. Trigger Point Therapy (Also known as: Deep Dry Needling, Dry Needling, Myofascial Trigger Point Therapy, Neuromuscular Therapy, NMT, Positional Release Therapy, PRT, TPT, Trigger Point Injection, Trigger Point Management.)
561. Turmeric
a. Turmeric (Scientific name: Curcuma longa, synonym Curcuma domestica; Curcuma aromatica. Also known as: Curcuma, Curcumae Longa, Curcumae Longae Rhizoma, Curcumin, Curcumine, Curcuminoid, Curcuminoïde, Curcuminoïdes, Curcuminoids, Halada, Haldi, Haridra, Indian Saffron, Nisha, Pian Jiang Huang, Racine de Curcuma, Radix Curcumae, Rajani, Rhizoma Cucurmae Longae, Safran Bourbon, Safran de Batallita, Safran des Indes, Turmeric Root, Yu Jin.)
b. Javanese turmeric (Scientific name: Curcuma xanthorrhiza. Also known as: Also known as: Curcuma, Curcuma de Java, Curcuma Javanais, Cúrcuma Javanesa, Curcumae Xanthorrhizae Rhizoma, Java Turmeric, Safran des Indes, Témoé-Lawacq, Témoé-Lawaq, Temu Lawak, Temu Lawas, Tewon Lawa.)
c. Tree Turmeric (Scientific name: Berberis aristata, synonyms Berberis chitria, Berberis coriaria. Also known as: Bérbero Indio, Chitra, Citra, Darhahed, Darhald, Daruhaldi, Daruharidra, Darurajani, Darvi, Épine-Vinette Aristée, Hint Amberparisi, Indian Barberry, Indian Berberry, Indian Lycium, Indian Ophthalmic Barberry, Nepal Barberry, Nepalese Barberry, Ophthalmic Barberry, Pisse Vinaigre, Vinettier Aristé.)
562. Tyrosine (Scientific name: Tyrosine. Also known as: 2-Acetylamino-3-(4-Hydroxyphenyl)-Propanoic Acid, 2-Amino-3-(4-Hydroxyphenyl)-propionic acid, Acetyl-L-Tyrosine, Acétyl-L-Tyrosine, L-Tyrosine, N-Acetyl L-Tyrosine, N-Acetyl-L-Tyrosine, N-Acétyl L-Tyrosine, N-Acetyl-Tyrosine, N-Acétyl-Tyrosine, Tirosina, Tyr, Tyrosinum.)
563. Unani Medicine (Also known as: Graeco-Arabic Medicine, Médecine Gréco-Arabe, Médecine Unani, Médecine Yunâni, Medicina Unani, Système Médical Unani, Unaani, Unani, Unani Healing, Unani Médecine, Unani Therapy, Unani Tibb, Yunâni.)
564. Unconventional Medicine (Also known as: Unconventional Therapy, Unconventional Approach, Unconventional Health.)
565. Unorthodox Practice
566. Unsaturated Fatty Acids
567. Urine Therapy (Also known as: Amaroli, Auto-Urine Therapy, Auto-Urotherapy, Mutra Paribhasa, Mutra Varga, Naramutra, Shivambu, Uro-Therapy, Urotherapy.)
568. Valerian
a. Valerian (Scientific name: Valeriana officinalis, Valeriana edulis, Valeriana angustifolia, Valeriana jatamansii, synonym Valeriana wallichii, Valeriana sitchensis, Valeriana fauriei. Also known as: All-Heal, Amantilla, Baldrian, Baldrianwurzel, Belgium Valerian, Common Valerian, Fragrant Valerian, Garden Heliotrope, Garden Valerian, Grande Valériane, Guérit Tout, Herbe à la Femme Meurtrie, Herbe aux Chats, Herbe aux Coupures, Herbe de Notre-Dame, Herbe de Saint-Georges, Herbe du Loup, Indian Valerian, Mexican Valerian, Pacific Valerian, Rhizome de Valériane, Tagar, Tagar-Ganthoda, Tagara, Valeriana, Valeriana Pseudofficinalis, Valeriana Rhizome, Valerianae Radix, Valeriane, Valériane, Valériane à Petites Feuilles, Valériane Africaine, Valériane Celtique, Valériane Commune, Valériane de Belgique, Valériane des Collines, Valériane Dioïque, Valériane du Jardin, Valériane Indienne, Valériane Mexicaine, Valériane Officinale, Valériane Sauvage.)
b. Red-Spur Valerian (Scientific name: Centranthus ruber, synonym Valeriana rubra. Also known as: Alfeñique, Barbe de Jupiter, Bouncing Bess, Bovis and Soldier, Centranthe Rouge, Centranto, Delicate Bess, Drunken Sailor, Fox's-Brush, Jupiter's Beard, Lilas d'Espagne, Milamores, Pretty Betsy, Red Spur Valerian, Red Valerian, Valeriana Roja, Valériane des Jardins, Valériane Rouge.)
569. Vanadium (Scientific name: V, Atomic number 23. Also known as: Metavanadate, Métavanadate, Orthovanadate, Pentoxyde de Vanadium, Sulfate de Vanadyl, Vanadate, Vanadio, Vanadium Pentoxide, Vanadyl, Vanadyl Nicotinate, Vanadyl Sulfate, Vanadyl Sulphate.)
570. Vanilla (Scientific name: Vanilla planifolia, synonyms Vanilla fragrans, Myrobroma fragrans; Vanilla tahitensis. Also known as: Bourbon Vanilla, Common Vanilla, Madagascar Vanilla, Mexican Vanilla, Réunion Vanilla, Tahiti Vanilla, Tahitian Vanilla, Vainilla, Vanille, Vanille Bourbon, Vanille de Bourbon, Vanille de Madagascar, Vanille du Mexique, Vanille de Tahiti.)
571. Vegan Diet
572. Vegetarian Diet (Also known as: Flexitarian Diet, Fruitarianism Diet, Lacto-Vegetarian Diet, Ovo-Vegetarian Diet, Pescotarian Diet, Plant-Based Diet, Semi-vegetarian Diet, Vegan Diet, Vegetarianism, Whole Food Plant-Based Diet.)
573. Vitamin A (Also known as: 3-Dehydroretinol, 3-Déhydrorétinol, Acétate de Rétinol, Antixerophthalmic Vitamin, Axerophtholum, Dehydroretinol, Déhydrorétinol, Fat-Soluble Vitamin, Oleovitamin A, Palmitate de Rétinol, Retinoid, Retinoids, Rétinoïdes, Retinol, Rétinol, Retinol Acetate, Retinol Palmitate, Retinyl Acetate, Rétinyl Acétate, Retinyl Palmitate, Rétinyl Palmitate, Vitamin A Acetate, Vitamin A Palmitate, Vitamin A1, Vitamin A2, Vitamina A, Vitamine A, Vitamine A1, Vitamine A2, Vitamine Liposoluble, Vitaminum A.)
574. Vitamin B12 (Scientific name: Vitamin B12, Cyanocobalamin, Hydroxocobalamin, Methylcobalamin. Also known as: B-12, B12, B Complex, B Complex Vitamin, Bedumil, Cobalamin, Cobalamine, Cobamin, Cobamine, Complexe Vitaminique B, Cyanocobalamine, Cycobemin, Hydroxycobalamine, Hydroxocobalaminum, Hydroxocobemine, Hydroxocobémine, Idrossocobalamina, Méthylcobalamine, Vitadurin, Vitadurine, Vitamina B12, Vitamine B12.)
575. Vitamin B2 (Scientific name: Riboflavin. Also known as: B Complex Vitamin, Complexe de Vitamines B, Flavin, Flavine, Lactoflavin, Lactoflavine, Riboflavin 5' Phosphate, Riboflavin Tetrabutyrate, Riboflavina, Riboflavine, Vitamin B2, Vitamin G, Vitamina B2, Vitamine B2, Vitamine G.)
576. Vitamin B3
a. Niacin (Scientific name: 3-Pyridinecarboxylic Acid, Niacin, Nicotinic acid, Vitamin B3. Also known as: 3-Pyridinecarboxylic Acid, Acide Nicotinique, Acide Pyridine-Carboxylique-3, Anti-Blacktongue Factor, Antipellagra Factor, B Complex Vitamin, Complexe de Vitamines B, Facteur Anti-Pellagre, Niacina, Niacine, Nicosedine, Pellagra Preventing Factor, Vitamin PP, Vitamina B3, Vitamine B3, Vitamine PP.)
b. Niacinamide (Scientific name: Pyridine-3-carboxamide. Also known as: 3-Pyridine Carboxamide, 3-Pyridinecarboxamide, Amide de l'Acide Nicotinique, B Complex Vitamin, Complexe de Vitamines B, Niacinamida, Nicamid, Nicosedine, Nicotinamide, Nicotinic Acid Amide, Nicotylamidum, Vitamin B3, Vitamin B3a, Vitamina B3, Vitamine B3.)
577. Vitamin B5 (Scientific name: D-Pantothenic Acid; Pantothenic Acid. Also known as: Acide D-Pantothénique, Acide Pantothénique, Ácido Pantoténico, Alcool Pantothénylique, B Complex Vitamin, Calcii Pantothenas, Calcium D-Pantothenate, Calcium Pantothenate, Complexe de Vitamines B, D-Calcium Pantothenate, D-Panthenol, D-Panthénol, D-Pantothénate de Calcium, D-Pantothenyl Alcohol, Dexpanthenol, Dexpanthénol, Dexpanthenolum, Pantéthine, Panthenol, Panthénol, Pantothenate, Pantothénate, Pantothénate de Calcium, Pantothenol, Pantothenylol, Vitamin B-5, Vitamin B5, Vitamina B5, Vitamine B5.)
578. Vitamin B6 (Scientific name: Pyridoxine, Pyridoxal, Pyridoxamine, Pyridoxine-5'-Phosphate, Pyridoxal-5'-Phosphate, Pyridoxamine-5'-Phosphate. Also known as: Adermine Chlorhydrate, Adermine Hydrochloride, B Complex Vitamin, B6, Chlorhydrate de Pyridoxine, Complexe de Vitamines B, Phosphate de Pyridoxal, Phosphate de Pyridoxamine, Piridoxina, Pyridoxal Phosphate, Pyridoxal 5 Phosphate, Pyridoxal-5-Phosphate, Pyridoxamine Hydrochloride, Pyridoxamine Phosphate, Pyridoxine HCl, Pyridoxine Hydrochloride, Pyridoxine Phosphoserinate, Pyridoxine-5-Phosphate, P5P, P-5-P, Vitamin B-6, Vitamina B6, Vitamine B6.)
579. Vitamin B7 (Scientific name: Cis-hexahydro-2-oxo-1H-thieno[3,4-d]-imidazole-4-valeric acid. Also known as: Biotin, Biotina, Biotine, Biotine-D, Coenzyme R, D-Biotin, Vitamin B7, Vitamin H, Vitamine B7, Vitamine H, W Factor.)
580. Vitamin C (Scientific name: Ascorbic Acid. Also known as: Acide Ascorbique, Acide Cévitamique, Acide Iso-Ascorbique, Acide L-Ascorbique, Acido Ascorbico, Antiscorbutic Vitamin, Ascorbate, Ascorbate de Calcium, Ascorbate de Sodium, Ascorbic Acid, Ascorbyl Palmitate, Calcium Ascorbate, Cevitamic Acid, Iso-Ascorbic Acid, L-Ascorbic Acid, Magnesium Ascorbate, Palmitate d'Ascorbyl, Selenium Ascorbate, Sodium Ascorbate, Vitamina C, Vitamine Antiscorbutique, Vitamine C.)
581. Vitamin D
a. Vitamin D (Scientific name: 1, 25-Dihydroxycholecalciferol, 25-Hydroxycholecalciferol, Alfacalcidol, Calcifediol, Calcipotriene, Calcitriol, Cholecalciferol, Dihydrotachysterol, Ergocalciferol, Paricalcitol. Also known as: Alfacalcidol: 1-Alpha-Hydroxycholecalciferol, 1-Alpha-Hydroxycholécalciférol, 1alpha(OH)D3, Vitamina D, Vitamine D.)
b. Calcifediol (Also known as: 25-HCC, 25-hydroxycholecalciferol, 25-hydroxycholécalciferol, 25-hydroxyvitamin D3, 25-hydroxyvitamine D3, 25-OHCC, 25-OHD3, Calcifédiol.)
c. Calcipotriene (Also known as: Calcipotriène, Calcipotriol.)
d. Calcitriol (Also known as: 1,25-DHCC, 1,25-dihydroxycholecalciferol, 1,25-dihydroxycholécalciférol, 1,25-dihydroxyvitamin D3, 1,25-dihydroxyvitamine D3, 1,25-diOHC, 1,25(OH)2D3.)
e. Cholecalciferol (Also known as: 7-déhydrocholestérol Activé, Activated 7-dehydrocholesterol, Cholécalciférol, Colecalciferol, Colécalciférol, Vitamin D3.)
f. Dihydrotachysterol (Also known as: DHT, Dihydrotachystérol, dihydrotachysterol 2, dichysterol, Vitamine D3.)
g. Ergocalciferol (Also known as: Activated Ergosterol, Calciferol, Ergocalciférol, Ergocalciferolum, Ergostérol Activé, Irradiated Ergosterol, Ergostérol Irradié, Viosterol, Viostérol, Vitamin D2, Vitamine D2.)
h. Paricalcitol (Also known as: 19-nor-1,25-dihydroxyvitamin D2, 19-nor-1,25-dihydroxyvitamine D2, Paracalcin.)
582. Vitamin E (Scientific name: Alpha-Tocopherol, Alpha Tocotrienol, Beta-Tocopherol, Beta Tocotrienol, Delta-Tocopherol, Delta Tocotrienol, Gamma-Tocopherol, Gamma Tocotrienol. Also known as: Acétate d'Alpha Tocophérol, Acétate d'Alpha Tocophéryl, Acétate de D-Alpha-Tocophéryl, Acétate de DL-Alpha-Tocophéryl, Acétate de Tocophérol, Acétate de Tocophéryl, Acétate de Vitamine E, All-Rac-Alpha-Tocophérol, All Rac-Alpha-Tocopherol, Alpha-Tocophérol, Alpha Tocopherol Acetate, Alpha Tocopheryl Acetate, Alpha Tocotriénol, Bêta-Tocotriénol, Bêta-Tocophérol, Concentré de Tocotriénol, D-Alpha Tocopherol, D-Alpha Tocophérol, D-Alpha Tocopheryl Acetate, D-Alpha-Tocopherol, D-Alpha-Tocophérol, D-Alpha-Tocopheryl, D-Alpha-Tocopheryl Acetate, D-Alpha Tocopheryl Acetate, D-Alpha-Tocophéryl, D-Alpha-Tocopheryl Acid Succinate, D-Alpha-Tocopheryl Succinate, D-Alpha Tocotrienol, D-Alpha Tocotriénol, DL-Alpha-Tocopherol, DL-Alpha-Tocopheryl, DL-Alpha-Tocopheryl Acetate, D-Tocopherol, D-Tocopheryl Acetate, DL-Tocopherol, D-Beta-Tocopherol, D-Bêta-Tocophérol, D-Delta-Tocopherol, D-Delta-Tocophérol, Delta-Tocotriénol, Delta-Tocophérol, D-Gamma-Tocopherol, D-Gamma Tocotrienol, D-Gamma-Tocotriénol, D-Gamma-Tocophérol, DL-Alpha-Tocophérol, DL-Alpha-Tocophéryl, DL-Tocophérol, D-Tocophérol, Fat-Soluble Vitamin, Gamma-Tocotriénol, Gamma-Tocophérol, Mixed Tocopherols, Mixed Tocotrienols, Palm Tocotrienols, Rice Tocotrienols, RRR-Alpha-Tocopherol, RRR-Alpha-Tocophérol, Succinate Acide de D-Alpha-Tocophéryl, Succinate Acide de Tocophéryl, Succinate de D-Alpha-Tocophéryl, Succinate de Tocophéryl, Succinate de Vitamine E, Tocopherol, Tocopherol Acetate, Tocophérols Mixtes, Tocopheryl Acetate, Tocopheryl Acid Succinate, Tocopheryl Succinate, Tocotrienol, Tocotriénol, Tocotrienol Concentrate, Tocotriénols, Tocotrienols, Tocotriénols de Palme, Tocotriénols de Riz, Tocotriénols Mixtes, Vitamin E Acetate, Vitamin E Succinate, Vitamina E, Vitamine E, Vitamine Liposoluble, Vitamine Soluble dans les Graisses.)
583. Vitamin K
a. Vitamin K (Scientific name: Phytonadione (K1), Menaquinone (K2), Menadione (K3), Menadiol (K4), 4-amino-2-methyl-1-naphthol (K5). Also known as: Vitamin K1: Methylphytyl Naphthoquinone, Phylloquinone, Phytomenadione, Phytonadione, 2-Methyl-3-Phytyl-1,4-Naphthoquinone, Fat-Soluble Vitamin, Vitamina K, Vitamine K, Vitamine Liposoluble, Vitamine Soluble dans les Graisses.)
b. Vitamin K2 (Also known as: Menaquinone, Ménaquinone, Menatetrenone, Menatétrenone, MK-1, MK-2, MK-4, MK-5, MK-6, MK-7, MK-8, MK-9, MK-10, MK-11, MK-12, MK-13.)
c. Vitamin K3 (Also known as: Menadione, Ménadione, Menadione Sodium Bisulfite, 2-Methyl-1,4-Naphthoquinone.)
d. Vitamin K4 (Also known as: Menadiol, Menadiol Acetate, Menadiol Diacetate, Menadiol Sodium Diphosphate, Menadiol Sodium Phosphate, Menadiolum Solubile Methylnaphthohydroquinone.)
e. Vitamin K5 (Also known as: 4-Amino-2-Methyl-1-Naphthol.)
584. Vitamins
585. Vitex agnus-castus (Scientific name: Vitex agnus-castus, Vitex trifolia, Vitex rotundifolia. Also known as: Agneau du Moine, Agneau-chaste, Agni Casti, Agnocasto, Agnolyt, Agnus-Castus, Arbre au Poivre, Chaste Berry, Chaste Tree, Chaste Tree Berry, Chasteberry, Chastetree, Chinese Vitex, Fructus Agni Casti, Gattilier, Hemp Tree, Herbe au Poivre, Mang Jing Zi, Monk's Pepper, Panj-Angosht, Petit Poivre, Pimiento del Monje, Poivre de Moine, Poivre Sauvage, Vitex, Vitex Agnus Castus, Viticis Fructus.)
586. Vojta Method
587. Voodoo
588. Water Aerobics
589. Western Herbal Medicine (Also known as: Native American Herbs, Traditional Western Herbal Medicine, Western Herbalism, Western Herbs.)
590. White Willow (Scientific name: Salix alba. Also known as: European Willow.)
591. Whole Diet
592. Whole Medical Systems (Also known as: Whole Systems, Whole Practices, Whole-Body Approach.)
593. Whole-Body Vibration Therapy
594. Wigmore Diet
595. Wild Medicine
596. Witch Hazel (Scientific name: Hamamelis virginiana. Also known as: Avellano de Bruja, Café du Diable, Hamamelis, Hamamélis, Hamamélis de Virginie, Hazel, Noisetier des Sorcières, Snapping Tobacco Wood, Spotted Elder, Virginian Witch Hazel, Winter Bloom.)
597. Witchcraft Therapy (Also known as: Occult Therapy.)
598. Xiaxingci Granule
599. Yarrow (Scientific name: Achillea millefolium, synonyms Achillea borealis, Achillea lanulosa, Achillea magna. Also known as: Achilee, Achillea, Achillée, Achillée Boréale, Achillée Laineuse, Achillée Millefeuille, Acuilee, Band Man's Plaything, Bauchweh, Birangasifa, Birangasipha, Biranjasipha, Bloodwort, Bumadaran, Carpenter's Weed, Civan Percemi, Common Yarrow, Devil's Nettle, Devil's Plaything, Erba Da Cartentieri, Erba Da Falegname, Gandana, Gemeine Schafgarbe, Green Arrow, Herbe à la Coupure, Herbe à Dindes, Herbe aux Charpentiers, Herbe Militaire, Huile Essentielle d'Achillée, Katzenkrat, Little Feather, Milefolio, Milenrama, Milfoil, Millefeuille, Millefolii Flos, Millefolii Herba, Millefolium, Millegoglie, Noble Yarrow, Nosebleed, Old Man's Pepper, Plumajillo, Rajmari, Roga Mari, Sanguinary, Soldier's Wound Wort, Sourcil de Vénus, Staunchweed, Tausendaugbram, Thousand-Leaf, Wound Wort, Yarrow Essential Oil.)
600. Yohimbe (Scientific name: Pausinystalia yohimbe, synonyms Pausinystalia johimbe, Corynanthe johimbi, Corynanthe yohimbi. Also known as: 11-Hydroxy Yohimbine, Coryanthe Yohimbe, Corynanthe Johimbe, Johimbi, Yohimbehe, Yohimbehe Cortex, Yohimbine, Yohimbine HCl, Yohimbinum Muriaticum)
601. Zero Balancing (Also known as: Core Zero Balancing, ZB.)
602. Zhixian I Pill
603. Zinc (Scientific name: Zn, Atomic number 30. Also known as: Acétate de Zinc, Acexamate de Zinc, Aspartate de Zinc, Chelated Zinc, Chlorure de Zinc, Citrate de Zinc, Gluconate de Zinc, Méthionine de Zinc, Monométhionine de Zinc, Numéro Atomique 30, Orotate de Zinc, Oxyde de Zinc, Picolinate de Zinc, Polaprezinc, Pyrithione de Zinc, Sulfate de Zinc, Zinc Acetate, Zinc Acetylmethionate, Zinc Acexamate, Zinc Ascorbate, Zinc Aspartate, Zinc carbobenzoxy-beta-alanyltaurinate, Zinc Chelate, Zinc Chloride, Zinc Citrate, Zinc Difumarate Hydrate, Zinc Gluconate, Zinc Glycinate, Zinc Methionate, Zinc Methionine, Zinc Monomethionine, Zinc Murakab, Zinc Nicotinate, Zinc Orotate, Zinc Oxide, Zinc Picolinate, Zinc Pyrithione, Zinc Sulfate, Zinc Sulphate, Zincum Aceticum, Zincum Gluconicum, Zincum Metallicum, Zincum Valerianicum.)
604. Zishen Tongli Jianonang

## Discussion

In the present study, we created an operational definition of CAIM informed by a systematic search of four quality-assessed media sources. Prior to the creation of this operational definition, only one study conducted by Wieland et al.’s (2011) had developed an operational definition of CAM (excluding the term “integrative”) containing 70 therapies. This number was later expanded through addition of further examples to 259 therapies which are listed on the Cochrane Complementary Medicine website [39]. The present study’s operational definition contains 1561 unique terms which comprise 604 CAIM therapies, in which all 259 aforementioned therapies are included. This updated definition more than doubles the list of CAIM therapies and provides researchers with a considerably more comprehensive list of therapies to improve the conduct of future CAIM research involving systematic searches, such as reviews and bibliometric analyses.

### Perceived utility of an updated operational definition for future CAIM research

#### Systematic and scoping reviews

Systematic and scoping reviews are research methodologies which use repeatable analytical methods to search for, gather, and summarize literature on a given topic in order to fill a knowledge gap [40–45]. Standardized academic search strategies are of great benefit to researchers seeking to conduct these types of studies, as this yields more consistent search results; this in turn, can maximize the opportunity of capturing as much of the relevant available peer-reviewed literature as possible, identify gaps in research, and inform potential future directions [46–50]. This is no exception with respect to CAIM-specific research, however, conducting systematic searches within this particular field comes with its own particular challenges [51] due to the fact that prior to this study, there was no standardized list of terms used to inform a search. To date, the search strategies used to identify general CAIM-related literature appear to differ from researcher to researcher, undoubtedly resulting in selection biases with respect to therapies included [51]. Furthermore, indexed headings relating to CAIM are not standardized across different databases, nor is any single indexed term (or group of indexed terms) comprehensive enough to capture the entirety of the CAIM literature [51–54]. The standardized search strategies provided in this study can, therefore, mitigate this challenge by establishing a replicable collection of literature pertinent to the topic of CAIM. It can be anticipated that should future systematic and scoping reviews employ the search strategies informed by this operational definition, they will yield more comprehensive results, which will consequently serve to more comprehensively capture eligible articles regardless of the defined inclusion criteria.

#### Bibliometric analyses

A bibliometric analysis is a research methodology that involves the statistical assessment of scientific articles or books, to identify the characteristics and determine the impact of the literature published in a specific academic discipline [55–59]. Unlike the systematic or scoping review, however, no reporting guideline nor standard checklist for the conduct of a bibliometric analysis exists to date [60]; despite this, all bibliometric analyses typically share common similarities such as the assessing of one or more of the following characteristics: number of publications (in total and per year); open access status; articles per journals; journal names and impact factors; language of article; document type; publication country; author affiliations; funding sponsors; most highly published authors; and highest-cited articles [46, 61, 62]. Unlike the literature on other academic topics which may be captured by relatively short and simple search strategies, the conduct of a comprehensive bibliometric analysis specific to CAIM in general needed to include a defined list of CAIM therapies for the purpose of constructing the search strategy employed on academic databases. Hence, published bibliometric analyses on CAIM have been largely relegated to those assessing research published in CAIM journals [63–65], based on certain research methodologies (i.e. trials [66, 67]), or those specific to certain categories of CAIM (i.e. acupuncture [68, 69], apitherapy [70], yoga [71, 72] and homeopathy [73], among others). As with this operational definition’s utility to reviews, these standardized search strategies provided in this study can also mitigate these challenges by establishing a replicable collection of literature pertinent to the topic of CAIM [27]. It can be anticipated that should future bibliometric analyses employ search strategies informed by this operational definition, they will yield more comprehensive results, which will serve to better monitor the development, growth, and characteristics of the field of CAIM in general.

### Existing challenges and limitations of developing an operational definition of CAIM

While we anticipate that this operational definition of CAIM will provide much value to the future of CAIM research, it must be acknowledged that it is not without limitations, many of which are a result of a number of existing challenges that remain with respect to conducting research in this field.

#### Underinclusiveness and overinclusiveness: different countries, cultures, systems of traditional medicine and schools of thought

Firstly, it must be re-stated that CAIM therapies are and will likely forever be dynamic in nature, which makes it complex to define operationally. Even though our operational definition was informed by systematic searches, because there is no general standard nor agreement as to what constitutes a CAIM therapy, even among experts [27, 51], this list was therefore, constructed based on media sources largely authored by this group of individuals. It must also be acknowledged that certain types of CAIMs are inherently challenging to define. For example, certain CAIM therapies or systems of CAIM are difficult to categorize for multiple reasons; they originate from different regions of the world, cultures, systems of traditional medicine, and schools of thought [4, 74]. For example, CAIM therapies rooted in Buddhist practices originate from Eastern cultures such as Cambodia, Thailand, Myanmar, Bhutan, and Sri Lanka where they may be considered part of conventional care, however, their usage is considered to be CAIM across many European and North American countries [75, 76]. This challenge is further compounded across certain categories of CAIM. Herbal therapies, for example are referred to in multiple different ways, which may include scientific names and multiple common names, the latter of which may be derived from multiple languages, and not necessarily just English [77]. For other therapies, whether they are defined as CAIM or not are situational, even if considered within the context of conventional medical care. One example of this includes chelation therapy, which is the primary treatment method of heavy metal poisoning, and in this context it is considered as conventional care. However, when this same therapy is used for the treatment of atherosclerosis, it is considered CAIM [39]. Another example of this includes vitamins; in the context of treating diseases resulting from vitamin deficiency, these therapies are considered part of conventional care, however, the use of large doses of vitamins, often many times greater than the recommended dietary allowance (known as megavitamin therapy or orthomolecular medicine) is typically categorized as CAIM [39, 78]. Thus, even the most well-constructed operational definition of CAIM will suffer from both underinclusiveness and overinclusiveness; certainly a deeper discussion of the contexts and settings whereby each of the therapies found on our list constitute CAIM is of value, however, this is beyond the scope of the present study. Despite this, the value added by this operational definition as a result of being informed by a systematic search strategy, includes the fact that it has largely captured the most common terms used to refer to the most commonly-researched CAIMs.

#### English-language bias

Apart from the aforementioned challenges in defining CAIM, the very fact that this operational definition was searched for and constructed based on only the English language and literature serves as a limitation. Language biases are an additional and significant obstacle to CAIM research, and no single language or group of languages can sufficiently capture the entirety of the CAIM literature. It can reasonably be inferenced that research conducted on CAIM therapies originating from a certain region or culture are commonly published in the local or national language(s). The majority of CAIM studies, however, are published by English-language non-CAIM journals and by Chinese-language CAIM journals [67]. It has also been found that while the English journals publish a higher frequency of studies reporting negative results associated with CAIM therapies, Chinese, Japanese, and Russian journals have published more positive results [79, 80]. Although little is known about CAIM research published in other languages, such stark differences in findings only based on the English versus Chinese, Japanese and Russian languages, likely mean that large degrees of variance in findings likely exist between other languages too [81, 82].

Our operational definition is, therefore, limited by an English-language and Western bias, as the umbrella term of CAIM itself is largely a Western concept [83]. As mentioned earlier, many traditional medicines comprise a part of conventional care in non-Western countries, and are sometimes regarded as safe and effective (if not safer and more effective) in comparison to conventional Western medicine [80, 84]. One example includes Kampo, a Japanese traditional medicine system which incorporates herbal medicine and acupuncture therapies, which is commonly practiced in tandem with Western medicine, and is partly covered by public health insurance in Japan [85–88]; such therapies arguably would not fall under a Japanese definition of CAIM. Another example includes traditional Chinese medicine (TCM), which like Kampo, is used in conjunction with Western medicine in China. Although it has existed for many thousands of years, in recent years the study of TCM has been studied, produced, and dosed based on modern research technologies [89, 90]. Furthermore, TCM has been standardized by the government of China in a gradual process, with complete diagnostic and treatment guidelines and basic principles that act as the basis of scientific research and education on TCM [89–91]. From a Chinese cultural perspective, therefore, TCM would by definition not be considered CAIM either. Similar challenges in Western countries to the CAIM status of some therapies originating from early professional or quasi-professional practitioners of Western medicine can also be observed. Among CAIM professions, disputes among practitioners can lead to divisions in how a given therapy is practiced and perceived. The chiropractic profession is one example with a turbulent history; while some practitioners sought to align themselves more closely with conventional medical practitioners, others acted to reject this notion [92, 93]. Today, the profession in North America still remains divided, however, a significant number of members have succeeded in advancing the profession to a point whereby certain jurisdictions regard chiropractic care as conventional medicine [94–96].

#### Lack of CAIM therapy categorization

Following the development of the operational definition, we opted not to categorize the list of CAIM therapies, and instead present it alphabetically. While this may be perceived as a limitation, this decision was made based on the fact that no international standard for categorizing CAIM therapies exist [27]. While it could be argued that the CAIM therapies could have been categorized based on an arbitrarily-selected categorization system (i.e. natural products, mind and body practices [1]), a subsequent challenge included the fact that no consensus exists regarding which CAIM therapies fall into which categories. While management of an additional layer of classifications may be an onerous task to conduct in this already ambitious present study, its operationalization may support further work in identifying categories of CAIM therapies. Further research seeking to develop consensus around both CAIM therapy categories, and the categories under which each CAIM therapy falls into, may be of value in the future, however, the achievement of this represents both a time-consuming and resource-intensive venture that is beyond the scope of our present study.

### Plans to update the operational definition

We propose a two-stage approach with respect to updating this operational definition of CAIM. This group of authors will convene annually to discuss the definition and the emergence of new publications that may influence the need to update. The group will decide via consensus on the need to update (yes/no). In the event of “no”, the group will meet the following year. In the event of a “yes” the group will post their decision either through social media or formal correspondence to the publishing journal. This will be followed by a formal update.

## Conclusion

The present study involved a systematic search of four quality-assessed information resource types which was used to create an operational definition of CAIM. While this operational definition is not without its limitations, it represents a highly comprehensive list of therapies. This operational definition can be used to improve the conduct of future CAIM research involving systematic searches, thereby supporting the harmonization of CAIM-related research through the provision of a standard of classification, as well as support improved collaboration between different research groups with a vested interest in this topic area.

## Supplementary Information

**Additional file 1.**

## Data Availability

All relevant data are included in this manuscript.

## Abbreviations

CAIM: Complementary alternative and integrative medicine
CAM: Complementary and alternative medicine
HONcode: Health On the Net Code of Conduct
MeSH: Medical Subject Heading
NCCIH: National Centre for Complimentary and Integrative Health
NCCAM: National Center for Complementary and Alternative Medicine
PRISMA: Preferred Reporting Items for Systematic Reviews and Meta-Analyses
PROSPERO: Prospective Register of Systematic Reviews
TCM: Traditional Chinese medicine
