# Supplementary material for: Operational definition of complementary, alternative, and integrative medicine derived from a systematic search
Source: BMC Complement Med Ther. 2022 Apr 12;22:104. doi: 10.1186/s12906-022-03556-7 (PMC9006507; doi:10.1186/s12906-022-03556-7)
Supplement: Supplementary file 1 — Additional file 1. [file 12906_2022_3556_MOESM1_ESM.docx]

**Supplementary File 1: Bibliography of Resources Used to Develop the Operational Definition of CAIM**

**Peer-Reviewed Articles**

1. Jones CH. The spectrum of therapeutic influences and integrative health care: classifying health care practices by mode of therapeutic action. J Altern Complement Med. 2005;11(5):937-44. doi: 10.1089/acm.2005.11.937.
2. Dunne R, Watkins J. Complementary medicine - some definitions. R Soc Health J. 1997;117(5):287-91. doi: 10.1177/146642409711700505.
3. Fennell D, Liberato AS, Zsembik B. Definitions and patterns of CAM use by the lay public. Complement Ther Med. 2009;17:71-7. doi: [10.1016/j.ctim.2008.09.002](http://dx.doi.org/10.1016/j.ctim.2008.09.002).
4. Hawks JH, Moyad MA. CAM: Definition and classification overview. Urol Nurs. 2003 Jun;23(3):221-3. Available from: https://pubmed.ncbi.nlm.nih.gov/12861741/
5. Institute of Medicine. Appendix A CAM therapies, practices, and systems. Washington (DC): National Academy of Sciences; 2005. 11. Available from: https://www.ncbi.nlm.nih.gov/books/NBK83796/?report=printable
6. Kisling LA, Stiegmann RA. Alternative Medicine.StatPearls Publishing. 2020 Jan; PMID: 30860755.
7. Koithan M. Introducing Complementary and Alternative Therapies. J Nurse Pract. 2009 Jan 1; 5(1):18-20.
8. Liem A, Rahmawati KD. The meaning of complementary, alternative, and traditional medicine among the Indonesian psychology community: a pilot study. Journal of Integrative Medicine. 2017 July; 15(4): 288 - 294.
9. McDonald K, Slavin S. My body, my life, my choice: practices and meanings of complementary and alternative medicine among a sample of Australian people living with HIV/AIDS and their practitioners. AIDS Care. 2010 Jul 16; 22(10): 1229-1235.
10. Ng JY, Boon HS, Thompson AK, Whitehead CR. Making sense of “alternative”, “complementary”, “unconventional”, and “integrative” medicine: exploring the terms and meanings through a textual analysis. BMC Complementary and Alternative Medicine. 2016; 16(134) 1-18.
11. Pavek RR. New MeSH terms add accessibility to alternative medicine literature. Altern Ther Health Med. 1996 Mar; 2(2):25-8..
12. Rankin-Box D. Complementary therapies: integration and interpretation. Br. J. 1997; 2(1).
13. Strauss JL. Coeytaux R. McDuffie J. Efficacy of complementary and alternative medicine therapies for posttraumatic stress disorder. J Evid Based Complementary Altern Med [Internet]. 2011 Aug; 19(3)
14. Tabish SA. Complementary and alternative healthcare: is it evidence-based? Int. J. Health Sci. Educ. 2008 Jan; 2(1).
15. Tataryn DJ. Paradigms of health and disease: A framework for classifying and understanding complementary and alternative medicine. J Altern Complement Med. 2002; 8(6).
16. Uehleke B, Saller R. Towards a European term for complementary and alternative medicine (CAM): Complementary European medicine (CEM). Forsch Komplementmed. 2011; 18(2):66-67. doi: [10.1159/000328034](https://doi.org/10.1159/000328034)
17. Wieland LS, Manheimer E, Berman BM. Development and classification of an operational definition of complementary and alternative medicine for the cochrane collaboration. Altern Ther Health Med. 2011; 17(2):50. PMID: 21717826
18. Williams JW, Gierisch JM, McDuffie J, Strauss JL, Nagi A. An overview of complementary and alternative medicine therapies for anxiety and depressive disorders: supplement to efficacy of complementary and alternative medicine therapies for posttraumatic stress disorder [Internet]. Washington DC: Department of Veterans Affairs (US); 2011 [cited 2021 Mar 15]. Appendix A: Search Strategies. Available from: https://www.ncbi.nlm.nih.gov/books/NBK82792/
19. Zollman C, Vickers A. What is complementary medicine? BMJ. 1999; 319:693-696. doi: [10.1136/bmj.319.7211.693](https://doi.org/10.1136/bmj.319.7211.693)

**“Aims and Scope” Webpages of Peer-Reviewed CAIM Journals**

1. BMC Complementary Medicine and Therapies [Internet]. Springer Nature. About; [cited 2021 Mar 15]. Available from: https://bmccomplementmedtherapies.biomedcentral.com/about
2. Journal of Integrative Medicine [Internet]. Netherlands: Elsevier. About the journal; [cited 2021 Mar 15]. Available from: https://www.sciencedirect.com/journal/journal-of-integrative-medicine/about/aims-and-scope
3. Integrative Cancer Therapies [Internet]. US: SAGE Journals. Aims and scope; [cited 2021 Mar 15]. Available from: <https://journals.sagepub.com/aims-scope/ICT>
4. Integrative Medicine Research [Internet]. Netherlands: Elsevier. About the journal; [cited 2021 Mar 15]. Available from: <https://www.sciencedirect.com/journal/integrative-medicine-research/about/aims-and-scope>
5. The Journal of Alternative and Complementary Medicine [Internet]. US: Mary Ann Liebert. About this publication; [cited 2021 Mar 15]. Available from: https://home.liebertpub.com/publications/journal-of-alternative-and-complementary-medicine-the/26/overview
6. Complementary Therapies in Medicine [Internet]. Netherlands: Elsevier. About the journal; [cited 2021 Mar 15]. Available from: <https://www.sciencedirect.com/journal/complementary-therapies-in-medicine/about/aims-and-scope>
7. [Evidence-Based Complementary and Alternative Medicine](https://www.hindawi.com/journals/ecam/) [Internet]. London, England: Hindawi. About this journal; [cited 2021 Mar 15]. Available from: <https://www.hindawi.com/journals/ecam/about/#contact>
8. Complementary Therapies in Clinical Practice [Internet]. Netherlands: Elsevier. About the journal; [cited 2021 Mar 15]. Available from: <https://www.sciencedirect.com/journal/complementary-therapies-in-clinical-practice/about/aims-and-scope>
9. Chinese Journal of Integrative Medicine [Internet]. Switzerland: Springer. Aims and scope; [cited 2021 Mar 15]. Available from: <https://www.springer.com/journal/11655/aims-and-scope>
10. Complementary Medicine Research [Internet]. Basel, Switzerland: Karger. About this journal; [cited 2021 Mar 15]. Available from:

<https://www.karger.com/Journal/Details/224242>

1. European Journal of Integrative Medicine [Internet]. Netherlands: Elsevier. About the journal; [cited 2021 Mar 15]. Available from: https://www.sciencedirect.com/journal/european-journal-of-integrative-medicine/about/aims-and-scope
2. Alternative Therapies in Health and Medicine [Internet]. About us; [cited 2021 Mar 15]. Available from: <http://www.alternative-therapies.com/index.cfm/fuseaction/Content.Main/id/42>
3. Journal of Traditional and Complementary Medicine [Internet]. Netherlands: Elsevier. About the journal; [cited 2021 Mar 15]. Available from: <https://www.sciencedirect.com/journal/journal-of-traditional-and-complementary-medicine/about/aims-and-scope>
4. Integrative Cancer Therapies [Internet]. US: SAGE Journals. About this journal; [cited 2021 Mar 15]. Available from: <https://journals.sagepub.com/home/ict>
5. Journal of Evidence-Based Integrative Medicine [Internet]. US: SAGE Journals. Aims and scope; [cited 2021 Mar 15]. Available from: <https://journals.sagepub.com/aims-scope/CHP>
6. Journal of Complementary and Integrative Medicine [Internet]. Berlin: De Gruyter. About this journal; [cited 2021 Mar 15]. Available from: <https://www.degruyter.com/journal/key/jcim/html>
7. Journal of Ayurveda and Integrative Medicine [Internet]. Netherlands: Elsevier. About the journal; [cited 2021 Mar 15]. Available from: <https://www.sciencedirect.com/journal/journal-of-ayurveda-and-integrative-medicine/about/aims-and-scope>
8. African Journal of Traditional, Complementary and Alternative Medicines [Internet]. Canada: Bioline International. African Journal of Traditional, Complementary and Alternative Medicines; [cited 2021 Mar 15]. Available from: <http://www.bioline.org.br/tc#:~:text=The%20African%20Journal%20of%20Traditional,food%20and%20agricultural%20technologies%2C%20and>
9. Advances in Integrative Medicine [Internet]. Netherlands: Elsevier. About the journal; [cited 2021 Mar 15]. Available from: <https://www.sciencedirect.com/journal/advances-in-integrative-medicine/about/aims-and-scope>
10. Integrative Medicine: A Clinician’s Journal [Internet]. US. About us; [cited 2021 Mar 15]. Available from: <http://www.imjournal.com/index.cfm/fuseaction/Content.Main/id/42>
11. Alternative and Complementary Therapies [Internet]. US: Mary Ann Liebert. About this publication; [cited 2021 Mar 15]. Available from: <https://home.liebertpub.com/publications/alternative-and-complementary-therapies/3/overview#:~:text=Alternative%20and%20Complementary%20Therapies%20is,or%20hospital%20integrative%20medicine%20programs>.
12. Focus on Alternative and Complementary Therapies [Internet]. US: Wiley. Overview; [cited 2021 Mar 15]. Available from: <https://onlinelibrary.wiley.com/page/journal/20427166/homepage/productinformation.html>

**Entries Containing CAIM Therapies in Highly-Accessed Online Encyclopaedias**

1. Britannica. Complementary and alternative medicine [Internet]. Chicago IL: Encyclopedia Britannica Inc.; 2018 Jan 3 [cited 2021 Mar 15]. Available from:<https://www.britannica.com/science/complementary-and-alternative-medicine>
2. HowStuffWorks. Integrative medicine: Taking alternatives mainstream [Internet]. HowStuffWorks; [cited 2021 Mar 15]. Available from:<https://health.howstuffworks.com/wellness/natural-medicine/alternative/alternative-medicine-goes-mainstream.htm>
3. HowStuffWorks. Alternative medicine goes mainstream [Internet]. HowStuffWorks; [cited 2021 Mar 15]. Available from:<https://health.howstuffworks.com/wellness/natural-medicine/alternative/alternative-medicine-goes-mainstream.htm>
4. Encyclopedia.com. Complementary and alternative medicine [Internet]. Cengage; [cited 2021 Mar 15]. Available from:<https://www.encyclopedia.com/medicine/educational-magazines/complementary-and-alternative-medicine>
5. Encyclopedia.com. Trends in alternative medicine [Internet]. Cengage; [cited 2021 Mar 15]. Available from:<https://www.encyclopedia.com/science/encyclopedias-almanacs-transcripts-and-maps/trends-alternative-medicine>
6. Encyclopedia.com. Alternative, complementary, and integrative medicine [Internet]. Cengage; [cited 2021 Mar 15]. Available from:<https://www.encyclopedia.com/education/encyclopedias-almanacs-transcripts-and-maps/alternative-complementary-and-integrative-medicine>
7. Encyclopedia.com. Alternative medicine [Internet]. Cengage; [cited 2021 Mar 15]. Available from:<https://www.encyclopedia.com/medicine/divisions-diagnostics-and-procedures/medicine/alternative-medicine>
8. Encyclopedia.com. Alternative and complementary treatments [Internet]. Cengage; [cited 2021 Mar 15]. Available from:<https://www.encyclopedia.com/science/medical-magazines/alternative-and-complementary-treatments>
9. Encyclopedia.com. Alternative treatments [Internet]. Cengage; [cited 2021 Mar 15]. Available from:<https://www.encyclopedia.com/science/medical-magazines/alternative-treatments>
10. Infoplease. Alternative medicine [Internet]. Sandbox Network Inc.; [cited 2021 Mar 15]. Available from:<https://www.infoplease.com/encyclopedia/medicine/general/terms/alternative-medicine>
11. Paranormal-Encyclopedia.com. Alternative medicine [Internet]. Paranormal Encyclopedia.com; [cited 2021 Mar 15]. Available from:<https://www.paranormal-encyclopedia.com/a/alternative-medicine/>

**Highly Ranked Websites Resulting From Health On the Net Code Of Conduct (Honcode) Searches**

1. Research Council for Complementary Medicine. What is CAM [Internet]. London, EN: [cited 2021 Mar 15]. Available from: <https://www.rccm.org.uk/what-is-cam/>.
2. National Heart, Lung, and Blood Institute (NHLBI). Asthma guidelines – EPR3 literature review search strategy progress summary. NHLBI. 1.
3. National Library of Medicine. Pubmed subject filters [Internet]. Bethesda, MD: National Library of Medicine (US); 2020 Feb 26 [cited 2021 Mar 15]. Available from: https://www.nlm.nih.gov/bsd/pubmed_subsets.html
4. National Library of Medicine. Search strategy used to create the PubMed complementary medicine filter [Internet]. Bethesda, MD: National Library of Medicine (US); 2019 Feb 20 [cited 2021 Mar 15]. Available from:<https://www.nlm.nih.gov/bsd/pubmed_subsets/comp_med_strategy.html>
5. National Library of Medicine. Complementary medicine - new PubMed subset [Internet]. Bethesda, MD: National Library of Medicine (US); 2001 Feb 5 [cited 2021 Mar 15]. Available from: <https://www.nlm.nih.gov/pubs/techbull/jf01/jf01_cam.html>
6. World Health Organization. Traditional, complementary and integrative medicine [Internet]. World Health Organization; [cited 2021 Mar 15]. Available from: <https://www.who.int/health-topics/traditional-complementary-and-integrative-medicine#tab=tab_1>
7. National Library of Medicine. Topic-specific PubMed queries [Internet]. Bethesda, MD: National Library of Medicine (US); 2021 May 10 [cited 2021 Mar 15]. Available from:<https://www.nlm.nih.gov/psd/special_queries.html>
8. World Health Organization. WHO global report on traditional and complementary medicine. Geneva, CH: World Health Organization; 2019. 226p.
9. Better Health Channel. Complementary therapies [Internet]. Victoria, AU: Victoria State Government; 2021 Jul 29 [cited 2021 Mar 15]. Available from:<https://www.betterhealth.vic.gov.au/health/conditionsandtreatments/complementary-therapies>
10. Drugs.com. Alternative medicines [Internet]. Drugs.com; [cited 2021 Mar 15]. Available from:<https://www.drugs.com/drug-class/alternative-medicines.html>
11. MedicineNet. Medical definition of alternative medicine [Internet]. MedicineNet; 2021 Mar 29 [cited 2021 Mar 15]. Available from:<https://www.medicinenet.com/alternative_medicine/definition.htm>
12. Merck Manuals Consumer Version. Types of complementary and alternative medicine [Internet]. Kenilworth, NJ: Merck Sharp & Dohme Corp; 2019 Feb [cited 2021 Mar 15]. Available from:<https://www.merckmanuals.com/home/special-subjects/integrative-complementary-and-alternative-medicine/types-of-complementary-and-alternative-medicine>
13. Food and Drug Administration. Complementary and alternative medicine products and their regulation by the food and drug administration. Rockville, MD: Food and Drug Administration; 2007 Feb. 14.
14. Mayo Clinic. Integrative medicine: overview [Internet]. Mayo Foundation for Medical Education and Research; 2020 Jun 19 [cited 2021 Mar 15]. Available from: https://www.mayoclinic.org/tests-procedures/complementary-alternative-medicine/about/pac-20393581
15. World Health Organization. WHO global report on traditional and complementary medicine 2019. Geneva, CH: World Health Organization; 2019 Jun 4. 226.
16. Healthline. What does a holistic doctor do? [Internet]. Healthline Media; 2020 Jun 24 [cited 2021 Mar 15]. Available from:<https://www.healthline.com/health/holistic-doctor#summary>
17. Healthline. The best holistic health blogs of 2020 [Internet]. Healthline Media; 2020 Jul 15 [cited 2021 Mar 15]. Available from:<https://www.healthline.com/health/holistic-health-best-blogs-of-the-year#1>
18. Healthline. The best alternative medicine apps of the year [Internet]. Healthline Media; 2019 Apr 16 [cited 2021 Mar 15]. Available from:<https://www.healthline.com/health/alternative-medicine-apps-iphone-android>
19. Healthline. The best alternative medicine blogs of the year [Internet]. Healthline Media; 2017 Jul 11 [cited 2021 Mar 15]. Available from:<https://www.healthline.com/health/alternative-medicine-best-blogs-of-the-year>
20. Mayo Clinic. Integrative medicine: departments and specialties [Internet]. Mayo Foundation for Medical Education and Research; 2020 Jun 19 [cited 2021 Mar 15]. Available from:<https://www.mayoclinic.org/tests-procedures/complementary-alternative-medicine/doctors-departments/pdc-2039358>
21. Cleveland Clinic. Center for Functional Medicine [Internet]. Cleveland, OH: Cleveland Clinic; [cited 2021 Mar 15]. Available from: <https://my.clevelandclinic.org/departments/functional-medicine>
22. Cleveland Clinic. Center for Functional Medicine [Internet]. Cleveland, OH: Cleveland Clinic; [cited 2021 Mar 15]. Available from: <https://my.clevelandclinic.org/departments/functional-medicine/about>
23. Interstitial Cystitis Association. Holistic Medicine vs. Homeopathy...Is there a Difference? [Internet]. McLean, VA: Interstitial Cystitis Association; [cited 2021 Mar 15]. Available from: <https://www.ichelp.org/holistic-medicine-vs-homeopathyis-there-a-difference/>
24. Waddington C, Egger D. Integrated Health Services—What and Why? [Internet]. World Health Organization; [cited 2021 Mar 15]. Available from: <https://www.who.int/healthsystems/service_delivery_techbrief1.pdf>
25. Waddington C, Egger D. Integrated Health Services—What and Why? [Internet]. World Health Organization; [cited 2021 Mar 15]. Available from: <https://www.who.int/healthsystems/technical_brief_final.pdf>?
26. Whitmer M. Understanding Complementary and Alternative Medicine. [Internet]. The Mesothelioma Center; 2021 July 23 [cited 2021 Mar 15]. Available from: <https://www.asbestos.com/treatment/alternative/>
27. Breastcancer.org. Types of Complementary Techniques [Internet]. Ardmore, PA: 2020 Nov 23 [cited 2021 Mar 15]. Available from: <https://www.breastcancer.org/treatment/comp_med/types>
28. Simon S. The Truth About Alternative Medical Treatments. [Internet]. American Cancer Society; 2019 Jan 30 [cited 2021 Mar 15]. Available from: <https://www.cancer.org/latest-news/the-truth-about-alternative-medical-treatments.html>
29. American Cancer Society. Marijuana and Cancer [Internet]. American Cancer Society; 2022 Aug 4 [cited 2021 Mar 15]. Available from: <https://www.cancer.org/treatment/treatments-and-side-effects/complementary-and-alternative-medicine/marijuana-and-cancer.html>
30. Wong C. 12 Most Common Questions About Insurance and Alternative Medicine [Internet]. Dotdash; revised 2021 Sept 17 [cited 2021 Mar 15]. Available from: <https://www.verywellhealth.com/types-of-complementary-and-alternative-medicine-88741>
31. WebMD. Complementary vs. Alternative Medicine: What’s the Difference? [Internet]. WebMD LLC; 2021 April 26 [cited 2021 Mar 15]. Available from: <https://www.webmd.com/balance/guide/complementary-vs-alternative-medicine#1>
32. Griffin RM. Alternative Treatments for Chronic Pain. [Internet]. Reviewed 2009 Mar 9 [cited 2021 Mar 15]. Available from: <https://www.webmd.com/pain-management/features/alternative-treatments#1>
33. MedlinePlus. Complementary and Integrative Medicine [Internet]. Bethesda, MD: National Library of Medicine (US); reviewed 2020 May 21 [cited 2021 Mar 15]. Available from: <https://medlineplus.gov/complementaryandintegrativemedicine.html>
34. Canadian Cancer Society. Complementary Therapies [Internet]. Toronto, ON: Canadian Cancer Society; [cited 2-21 Mar 15]. Available from: <https://www.cancer.ca/en/cancer-information/diagnosis-and-treatment/complementary-therapies/?region=on>
35. MacMillan Cancer Support. About Complementary Therapies [Internet]. London: MacMillan Cancer Support; [cited 2021 Mar 15]. Available from: <https://www.macmillan.org.uk/cancer-information-and-support/treatment/coping-with-treatment/complementary-therapies/about-complementary-therapies>
36. MedicineNet. Complementary and Alternative Medicine (CAM) [Internet]. MedicineNet; [date unknown] [cited 2021 Mar 15]. Available from: <https://www.medicinenet.com/alternative_medicine/article.htm>
37. Millstine D. Types of Complementary and Alternative Medicine [Internet]. Kenilworth, NJ: Merck Manual; 2018 Sept [cited 2021 Mar 15]. Available from: <https://www.merckmanuals.com/professional/special-subjects/integrative-complementary-and-alternative-medicine/types-of-complementary-and-alternative-medicine>
38. WebMD. Complementary Medicine: What are my choices? [Internet]. WebMD LLC; [date unknown] [cited 2021 Mar 15]. Available from: <https://www.webmd.com/cancer/holistic-treatment-17/complementary-medicine-cancer-chart>
39. MedlinePlus. Integrative medicine for cancer treatment [Internet]. Bethesda, MD: U.S. National Library of Medicine; updated 2021 Sept 1 [cited 2021 Mat 15]. Available from: <https://medlineplus.gov/ency/patientinstructions/000932.htm>
40. Breastcancer.org. Complementary and Holistic Medicine [Internet]. Ardmore, PA. Revised 2019 Jun 22 [cited 2021 Mar 15]. Available from: <https://www.breastcancer.org/treatment/comp_med>
41. Millstine D. Overview of Integrative, Complementary, and Alternative Medicine. [Internet]. Kenilworth, NJ: Merck Manual; 2018 Sept [cited 2021 Mar 15]. Available from: <https://www.merckmanuals.com/professional/special-subjects/integrative-complementary-and-alternative-medicine/overview-of-integrative-complementary-and-alternative-medicine>
42. University of Minnesota. What are Holistic Practices? [Internet]. Minnesota, USA: University of Minnesota; [date unknown] [cited 2021 Mar 15]. Available from: <https://www.takingcharge.csh.umn.edu/what-are-holistic-practices>
43. Cleveland Clinic. What is reiki, and does it really work? [Internet]. Cleveland OH: Cleveland Clinic; 2020 Aug 30 [cited 2021 Mar 15]. Available from:<https://my.clevelandclinic.org/ccf/media/files/Wellness/reiki-factsheet.pdf?fbclid=IwAR0UQ9iaXl36W8YL42p92ml02kuNwiN54vUEpu8q0Q4I65sFoP9OefXEVsc>
44. Cancer.Net. Integrative medicine [Internet]. American Society of Clinical Oncology (ASCO); [cited 2021 Mar 15]. Available from:<https://www.cancer.net/navigating-cancer-care/how-cancer-treated/integrative-medicine>
45. Cancer.Net. Types of complementary therapies [Internet]. American Society of Clinical Oncology (ASCO); 2021 Mar [cited 2021 Mar 15]. Available from:<https://www.cancer.net/navigating-cancer-care/how-cancer-treated/integrative-medicine/types-complementary-therapies>
46. Medical Home Portal. Integrative medicine for children and youth with special health care needs (CYSHCN) [Internet]. Salt Lake City UT: Department of Pediatrics University of Utah; 2013 Jan [cited 2021 Mar 15]. Available from:<https://www.medicalhomeportal.org/clinical-practice/common-issues-for-cyshcn/integrative-medicine-for-cyshcn>
47. WebMD. Whole medical systems: An overview [Internet]. WebMD LLC.; 2020 Feb 20 [cited 2021 Mar 15]. Available from:<https://www.webmd.com/balance/guide/understanding-alternative-medicine#1>
48. WebMD. What is holistic medicine? [Internet]. WebMD LLC.; 2020 Mar 18 [cited 2021 Mar 15]. Available from:<https://www.webmd.com/balance/guide/what-is-holistic-medicine#1>
49. WebMD. What is integrative medicine? [Internet]. WebMD LLC.; [cited 2021 Mar 15]. Available from:<https://www.webmd.com/cancer/holistic-treatment-17/integrative-medicine>
